# Chemical synthesis of perfectly isotactic and high melting bacterial poly(3-hydroxybutyrate) from bio-sourced racemic cyclic diolide

**DOI:** 10.1038/s41467-018-04734-3

**Published:** 2018-06-11

**Authors:** Xiaoyan Tang, Eugene Y.-X. Chen

**Affiliations:** Department of Chemistry, Colorado State University, Fort Collins, CO 80523-1872 USA

## Abstract

Bacterial poly(3-hydroxybutyrate) (P3HB) is a perfectly isotactic, crystalline material possessing properties suitable for substituting petroleum plastics, but high costs and low volumes of its production are impractical for commodity applications. The chemical synthesis of P3HB via ring-opening polymerization (ROP) of racemic *β*-butyrolactone has attracted intensive efforts since the 1960s, but not yet produced P3HB with high isotacticity and molecular weight. Here, we report a route utilizing racemic cyclic diolide (*rac*-DL) derived from bio-sourced succinate. With stereoselective racemic catalysts, the ROP of *rac*-DL under ambient conditions produces rapidly P3HB with perfect isotacticity ([*mm*] > 99%), high melting temperature (*T*_m_ = 171 °C), and high molecular weight (*M*_n_ = 1.54 × 10^5^ g mol^−1^, *Đ* = 1.01). With enantiomeric catalysts, kinetic resolution polymerizations of *rac*-DL automatically stops at 50% conversion and yields enantiopure (*R,R*)-DL and (*S,S*)-DL with >99% *e.e*. and the corresponding poly[(*S*)-3HB] and poly[(*R*)-3HB] with high *T*_m_ = 175 °C.

## Abstract (web summary)

Bacterial poly(3-hydroxybutyrate) possesses physical and mechanical properties suitable for substituting high-performance petroleum plastics but current production is costly and slow. Here the authors produce poly(3-hydroxybutyrate) with similar properties via ring-opening polymerization of bio-derived racemic cyclic diolide.

## Introduction

Poly(hydroxyalkanoate)s (PHAs)^1–12^, naturally produced by bacteria and other living microorganisms from biorenewable resources such as carbohydrates and fats, are an important class of commercially implemented aliphatic polyesters as biodegradable and/or biocompatible materials for biomedical, pharmaceutical, and packaging applications^13–20^. The most prominent, thus most extensively studied, PHA is poly(3-hydroxybutyrate) (P3HB), in which bacterial poly[(*R*)-3-hydroxybutyrate], P[(*R*)-3HB], is a perfectly stereoregular, pure isotactic crystalline thermoplastic material^1–12^. Thanks to its comparable thermal and mechanical properties to those of high-performance isotactic polypropylene (*it*-PP), highly isotactic P3HB is being considered as an attractive biodegradable alternative to petroleum-based plastics, especially *it*-PP. However, high current production costs and limited production volumes of bacterial/microbial PHAs, including P3HB, render them impractical in many applications in areas such as biorenewable and biodegradable green commodity thermoplastics.

The chemical synthesis via ring-opening polymerization (ROP) of cyclic esters (lactones or lactides), a process typically catalyzed by a metal-based or organic catalyst, offers an alternative route to such technologically important biodegradable aliphatic polyesters^21–28^, which is often advantageous, thanks to its fast kinetics of the polymerization, scalability of the polymer production, and tunability of catalysts and co-monomers. In this context, the ROP of *β*-butyrolactone (*β*-BL) has been developed for the chemical synthesis of P3HB^29–33^, while the ROP of *γ*-butyrolactone (*γ*-BL) for the chemical synthesis of poly(4-hydroxybutyrate) (P4HB)^34, 35^ has also been realized recently^36^. In the case of the ROP of *β*-BL, the cost-effective chemical synthesis of P3HB calls for the use of the *racemic* monomer, *rac*-*β*-BL, rather than the more expensive enantiopure (*R*)-*β*-BL, which requires a stereoselective ROP process to render the formation of isotactic P3HB. However, despite extensive research efforts since the 1960s, the chemical synthesis of P3HB with isotacticity *P*_m_ (defined as the probability of *meso* linkages between monomer units) >0.85 from the ROP of *rac*-*β*-BL has proven elusive. For example, the ROP of *rac*-*β*-BL by alkyl aluminoxanes produced a mixture of iso-enriched P3HB products as a whole (*P*_m_ = 0.62), which were fractionated into atactic and isotactic fractions with the highest *P*_m_ of 0.85 reported for the acetone-insoluble fraction^37–39^. A chiral initiator system consisting of ZnEt_2_/(*R*)(−)-3,3-dimethyl-1,2-butanediol also yielded a mixture of P3HB products that were fractionated in methanol into soluble atactic and insoluble (~25%) isotactic (*P*_m_ ~ 0.80) fractions^40^, and a chiral Co(salen)/Et_3_Al complex afforded P3HB of low crystallinity (no tacticity data)^41^. A discrete *β*-diiminate zinc alkoxide initiator promoted a controlled ROP of *rac*-*β*-BL with high polymerization rates, but the resulting P3HB is atactic^42^. Cr(III) salophen complexes converted *rac*-*β*-BL into iso-enriched P3HB with *P*_m_ = 0.66, medium number-average molecular weight (*M*_n_ = 4.81 × 10^4^ g mol^−1^), high dispersity (*Đ* = 5.2), and modest melting-transition temperature (*T*_m_ = 116, 142 °C)^43^. Lanthanide (La, Nd) borohydrides supported on silica polymerized *rac*-*β*-BL into P3HB with *P*_m_ = 0.85 and *M*_n_ = 1.15 × 10^4^ g mol^−1^^44^. It is worth noting that syndiotactic P3HB materials from being modestly syndiotactic (*P*_r_ ~ 0.70)^45, 46^ to highly syndiotactic (*P*_r_ up to 0.95)^47–49^, have been achieved through the ROP of *rac*-*β*-BL using alkyltinoxides and discrete yttrium complexes^50^ supported by tetradentate, dianionic alkoxy-amino-bis(phenolate) [*O*^−^,*N*,*O*,*O*^−^] ligands, respectively. This Y catalyst system has also been utilized for the ROP of functional *β*-lactones, such as 4-allyloxymethylene-*β*-propiolactone, to afford either highly isotactic (*P*_m_ = 0.93, *M*_n_ = 9.20 × 10^3^ g mol^−1^, *Đ* = 1.46) or syndiotactic (*P*_r_ = 0.91) polymer, depending on the substituents on the ligand^51^. In the context of creating a synthetic equivalent of bacterial P3HB, both high isotacticity (*P*_m_ > 0.95) and molecular weight (*M*_n_ > 10^5^ g mol^−1^) are typically required for practical use.

**Fig. 1:**
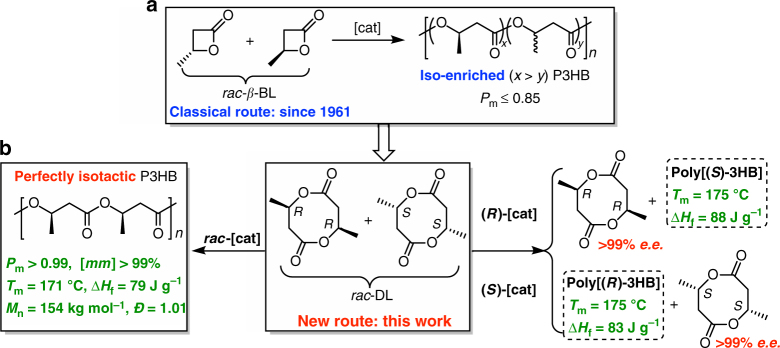
Chemical synthesis routes to P3HB. **a** Classical route via the ROP of *rac*-*β*-BL to iso-enriched P3HB. **b** The new route via the ROP of *rac*-DL to perfectly isotactic P3HB. (*P*_m_ is the probability of meso linkages between HB units, and *mm* is isotactic triad made up of two adjacent *meso* diads)

It is informative to point out that the ingenious utilization of a cyclic *dimer* of l-lactic acid (l-LA) rendered the commercial production of high-molecular-weight, highly isotactic polylactide (PLA)^52^, but the efforts in attempting the chemical synthesis of high-molecular-weight, highly isotactic P3HB have so far been strictly limited to the use of the cyclic *monomer* of 3-hydroxybutyric acid (3HB), namely *β*-BL. *β*-Lactones are known to be carcinogenic (alkylating DNA)^53^, and the competing ring-opening mechanisms (O-acyl vs. O-alkyl cleavage) were reported for the ROP of such highly strained monomers^54–56^. As the current approach via the ROP of *rac*-*β*-BL has yet so far produced P3HB with high molecular weight and high isotacticity (vide supra), we formulated the following three working hypotheses to address this long-standing challenge. First, by analogy of the PLA production, the chemical synthesis of P3HB could utilize a cyclic *dimer* of 3HB, namely eight-membered cyclic diolide (DL, Fig. 1), which is benign and can be readily derived from bio-sourced dimethyl succinate^57^. Worth noting here is that the enantiopure cyclic trimer of (*R*)-3HB, obtained in ~50% yield from the depolymerization of bacterial P[(*R*)-3HB]^58^, was attempted to polymerize back to P3HB but only low molecular weight (*M*_n_ ~ 5000 g mol^−1^) oligomers were obtained^59, 60^. Second, as DL possesses two stereogenic centers, the ROP of its *rac* and *meso* diastereomers could lead to various stereoregular (isotactic, syndiotactic, stereoblock, etc.) P3HB materials, depending on the stereoselectivity of the catalyzed ROP process. Third, considering significantly increased sterics present in DL relative to *β*-BL, we should anticipate a higher degree of stereochemical control in the catalyzed ROP of DL, thereby potentially generating highly stereoregular P3HB materials.

Guided by the above three hypotheses, we have investigated this proposed DL route to P3HB and hereby disclose our discovery that the catalyzed ROP of *rac*-DL (Fig. 1) enables the highly efficient synthesis of P3HB with perfect isotacticity, high crystallinity, high melting temperature, and high molecular weight.

## Results

### Stereoselective ROP of *rac*-DL

**Fig. 2:**
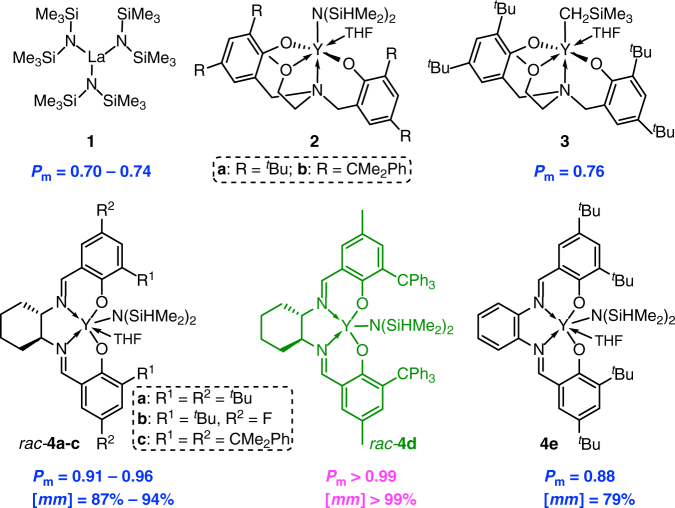
Chemical structures of the catalysts. Structures of catalysts employed in this study for the ROP of *rac*-DL and the isotacticity of the resulting P3HB materials they produced. (*P*_m_ is the probability of meso linkages between HB units, and *mm* is isotactic triad made up of two adjacent *meso* diads)

Pure *rac*-DL can be synthesized in a multi-gram scale from bio-sourced dimethyl succinate (Supplementary Figs. 2–6; see Supplementary Information for details.). At the outset, the ROP of *rac*-DL was screened with La[N(SiMe_3_)_2_]_3_ (**1**), a lanthanum complex that has been shown to be an effective catalyst for the ring-opening (co)polymerization of *γ*-BL and *α*-methylene-*γ*-butyrolactone (MBL)^36, 61, 62^. In combination with an alcohol initiator, the **1**/*x*BnOH (*x* = 2, 3, BnOH = benzyl alcohol) system was effective for the ROP of *rac*-DL (20 equiv) in dichloromethane (DCM) at room temperature (RT), achieving 100% conversion (*x* = 2) in 4 h or 98% (*x* = 3) conversion in 8 h, but the resulting P3HB exhibited a low molecular weight (*M*_n_ = 2.43 × 10^3^ g mol^−1^ and *Đ* = 1.09 for *x* = 3, Supplementary Table 1). Furthermore, both ^1^H and ^13^C NMR spectra (Supplementary Figs. 32, 33) showed the formation of an iso-biased amorphous material (*P*_m_ = 0.74, *x* = 2; *P*_m_ = 0.70, *x* = 3), which was confirmed by observation of only glass-transition temperature (*T*_g_) on its differential scanning calorimetry (DSC) curves (Supplementary Fig. 34). Next, we turned to a “privileged class of catalysts” for the ROP of cyclic esters^21^, discrete yttrium amido complexes **2** (**a**, R = ^*t*^Bu; **b**, R = CMe_2_Ph) and alkyl complex **3** supported by the tetradentate [*O*^−^,*N*,*O*,*O*^−^] ligand (Fig. 2), which were previously shown to be superior catalysts for the ROP of *γ*-BL and MBL^36, 61, 62^ as well as highly active catalysts for the syndiospecific ROP of *rac*-*β*-BL^47–49^. With a high precatalyst loading of 5 mol% and in combination with 1 equiv of BnOH initiator that undergoes instantaneous alcoholysis of the yttrium complex to generate the corresponding yttrium alkoxide catalyst^47–50^, all three yttrium complexes were active for the ROP of *rac*-DL, with yttrium alkyl **3** being the most active but still rather sluggish (76% conversion after 32 h). Moreover, these catalysts incorporating the tripodal alkoxy-amino-bis(phenolate) ligand afforded only low-molecular-weight (*M*_n_ = 2.70 × 10^3^ g mol^−1^ and *Đ* = 1.08 for catalyst **3**), iso-biased amorphous material (*P*_m_ = 0.76, Supplementary Table 1, Supplementary Figs. 35, 36). For catalysts **2**, only low conversions were achieved even after 48 h (44% by **2a**; 17% by **2b**), and the P3HB products were not isolated but the estimated *P*_m_ values by in situ NMR spectra were similar to that by catalyst **3**.

**Fig. 3:**
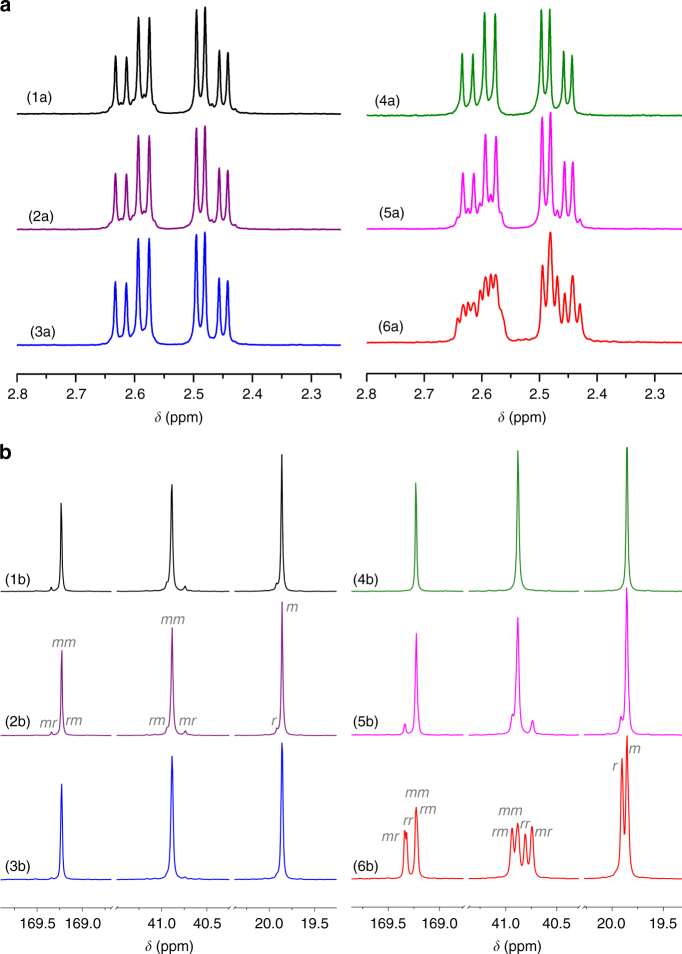
Stereomicrostructures (tacticities) of P3HB. **a** ^1^H NMR spectra (CDCl_3_) in the methylene region. **b** ^13^C NMR spectra (CDCl_3_) in the carbonyl, methylene, and methyl regions. P3HB produced by (1a–b) [*rac*-DL]/[**4a**] = 200/1; (2a–b) [*rac*-DL]/[**4b**] = 200/1; (3a–b) [*rac*-DL]/[**4c**] = 200/1; (4a–b) [*rac*-DL]/[**4d**] = 200/1; (5a–b) [*rac*-DL]/[**4e**] = 200/1; and (6a–b) comparative example of [*rac*-*β*-BL]/[**4d**] = 100/1

Considering the steric hindrance of *rac*-DL monomer with the eight-membered-ring framework and the low activity and isoselectivity of also sterically encumbered catalysts **1**–**3** screened, we arrived at sterically more open yttrium *racemic* salen complexes **4a**–**e** (Fig. 2). Yttrium silylamido complexes **4a**–**d** supported by *N,N′*-bis(salicylidene)cyclohexanediimine (salcy) ligands and complex **4e** supported by the analogous salph ligand were readily synthesized in good yields (58–83%, see Supplementary Information for details, Supplementary Figs. 13–22) according to the procedures established for the synthesis of known complex **4a**^63, 64^ and known salen ligands (Supplementary Figs. 7–13)^65–71^. Complex **4a** with the classic salen ligand bearing the 3, 5-di-*tert*-butyl substituents was first examined for its activity and stereoselectivity towards the ROP of *rac*-DL. Excitingly, this complex, when combined with 1 equiv of BnOH initiator, rapidly polymerized 20–200 equiv of *rac*-DL to completion within 20 min at RT. The molecular weight of the resulting P3HB increased from low *M*_n_ = 4.77 × 10^3^ g mol^−1^ (*Đ* = 1.17) to medium *M*_n_ = 3.20 × 10^4^ g mol^−1^ (*Đ* = 1.03) with increasing the [*rac*-DL]/[**4a**] ratio from 20/1 to 200/1, and the calculated initiation efficiency ranged from 74 to 108% (runs 1–4, Table 1), all indicating a controlled polymerization. More importantly, complex **4a** now yielded isotactic P3HB with *P*_m_ ~ 0.91–0.94 and isotactic [*mm*] triad ~ 87–89% based on ^1^H and ^13^C NMR analysis (Fig. 3). Accordingly, the resulting P3HB material became crystalline, exhibiting a *T*_m_ ~ 136–146 °C, depending on the polymer molecular weight (runs 1–4, Table 1). Replacing the initiator BnOH with ^*i*^PrOH lowered the polymerization activity by threefold, now requiring 60 min to achieve complete monomer (200 equiv) conversion. Furthermore, the molecular weight (*M*_n_ = 6.07 × 10^4^ g mol^−1^) of the resulting P3HB was considerably higher than the theoretical value, thus giving rise to a low initiation efficiency of only 57%, although the P3HB tacticity remained the same. Other alcohols such as Ph_2_CHCH_2_OH were also found less effective than BnOH. Therefore, all the subsequent polymerization studies with other yttrium complexes (**4b**–**e**) employed exclusively BnOH as the more effective initiator.

**Table 1:** Results of *rac*-DL polymerization by yttrium catalysts **4a–e** and BnOH initiator

Run	Catalyst	[*rac*-DL]/[**4**]	Time (min)	Conv.^a^ (%)	*M*_n_^b^ (kg mol^−1^)	*Ð*^b^ (*M*_w_/*M*_n_)	I^* c^ (%)	*P*_m_^d^	[*mm*]^d^ (%)	*T*_m_^e^ (°C)
1	**4a**	20/1	20	100	4.77	1.17	74	0.91	87	128/136
2	**4a**	50/1	20	100	10.9	1.05	80	0.93	87	133/143
3	**4a**	100/1	20	100	23.0	1.04	75	0.94	89	136/145
4	**4a**	200/1	20	100	32.0	1.03	108	0.93	89	146
5	**4b**	100/1	20	100	25.1	1.03	69	0.95	89	147
6	**4b**	200/1	20	100	37.3	1.01	93	0.95	88	147
7	**4c**	100/1	20	100	25.7	1.11	67	0.96	93	153/157
8	**4c**	200/1	20	100	52.7	1.14	66	0.96	94	156
9	**4d**	100/1	20	100	20.1	1.07	86	0.99	98	161
10	**4d**	200/1	20	100	37.4	1.07	92	>0.99	>99	164
11	**4d**	400/1	20	100	64.3	1.02	107	>0.99	>99	169
12	**4d**	800/1	60	98	119	1.03	113	>0.99	>99	170
13	**4d**	1200/1	30	71	154	1.01	95	>0.99	>99	171
14	**4e**	100/1	20	100	23.7	1.03	73	0.88	79	121
15	**4e**	200/1	20	100	43.6	1.24	79	0.89	79	122

Conditions: *rac*-DL = 0.138 g (0.80 mmol), [*rac*-DL] = 1.0 M; DCM as the solvent, *V*_solvent_ = 0.8 mL (except for run 13 where *V*_solvent_ = 0.4 mL); room temperature; yttrium catalyst **4** to BnOH initiator ratio fixed at 1/1, and the amount varied according to the [*rac*-DL]/[**4**] ratio

^a^Conversions of monomers measured by ^1^H NMR spectra of the quenched solution in benzoic acid/chloroform

^b^Weight-average molecular weights (*M*_w_), number-average molecular weights (*M*_n_), and dispersity indices (*Ð* = *M*_w_/*M*_n_) determined by GPC coupled with an 18-angle light scattering detector at 40 °C in chloroform

^c^The initiation efficiency I* = *M*_n_(calcd)/*M*_n_(exptl), where *M*_n_(calcd) = MW(*rac*-DL) × [*rac*-DL]/[**4**] × conv (%) + MW of chain-end groups (BnOH) = 172.18 × [*rac*-DL]/[**4**] × conv (%) + 108.14

^d^*P*_m_ is the probability of *meso* linkages between HB units, and *mm* is isotactic triad made up of two adjacent *meso* diads, determined by ^13^C{^1^H} NMR spectroscopy

^e^*T*_m_ measured by DSC with the cooling and second heating rate of 10 °C min^−1^ for samples produced by **4c**–**d**, 5 °C min^−1^ for samples produced by **4a**–**b**, or 2 °C min^−1^ for samples produced by **4e**

The above exciting results brought about by yttrium salen complex **4a** prompted us to investigate possible effects of the salen ligand framework’s electronics, sterics, and geometry of the backbone diimine linker on the *rac*-DL polymerization activity and stereoselectivity. In the context of electronic effects, electron withdrawing substituents (e.g., F) introduced to the 5-positions of the salicylidene framework were found to generate a more redox stable and active (salcy)Co(III) catalyst for the copolymerization of epoxide and anhydride monomer pairs^65^. In the present polymerization of *rac*-DL by (salcy)yttrium catalyst **4b** with the F groups substituted at the 5-positions of the salcy ligand, only the isotacticity of the resulting P3HB was improved slightly to now *P*_m_ = 0.95 and *T*_m_ = 147 °C (runs 5 and 6, Table 1), as compared with the parent *tert*-butyl substituted catalyst **4a**. Turning to the steric perturbation of the catalyst, the more bulky cumyl-substituted complex **4c** produced isotactic P3HB with a noticeably higher isotactic [*mm*] triad of 94% (Fig. 3) and *T*_m_ of 156 °C (run 8, Table 1), relative to the [*mm*] of 89% and *T*_m_ of 146 °C for the P3HB produced by catalyst **4a** under identical conditions (run 4 vs. 8, Table 1). These results showed much more pronounced effects of the ligand sterics than electronics on the polymerization stereoselectivity. Hence, the even more bulky trityl (Ph_3_C) groups were substituted at the 3-positions of the salcy ligand framework to generate catalyst racemic **4d**. Remarkably, catalyst **4d** produced essentially stereo-perfect, pure isotactic P3HB with *P*_m_ > 0.99 and [*mm*] > 99% (run 10, Table 1), while maintaining high polymerization activity (100% monomer conversion in 20 min). Consistent with this NMR-derived stereo-microstructure (Fig. 3), the resulting crystalline isotactic P3HB exhibited a high *T*_m_ of 164 °C. Lastly, to probe the possible effects of the geometry of the backbone diimine linker, we examined the performance of the salph-based complex **4e** for the *rac*-DL polymerization and found that this catalyst afforded P3HB with a considerably lower isotacticity (*P*_m_ ~ 0.88–0.89, [*mm*] = 79%, runs 14 and 15), as compared with the salcy-based analogue complex **4a** under identical conditions (*P*_m_ ~ 0.93–0.94, [*mm*] = 89%, runs 3 and 4). It is worth noting here that the high stereoselectivity of catalysts **4** observed for the ROP of *rac*-DL does not apply to the ROP of *rac*-*β*-BL; for example, the ROP of *rac*-*β*-BL by the best catalyst of the series, **4d**, was not only sluggish (even with a high catalyst loading of  1.0 mol% the reaction required 8 h to achieve 96% monomer conversion) but also non-stereoselective, producing atactic P3HB (Fig. 3, Supplementary Fig. 40). These results further highlight the importance of the steric interplay and matching between the catalyst and monomer structures to achieve a highly stereoselective ROP of such racemic lactone and diolide monomers.

**Fig. 4:**
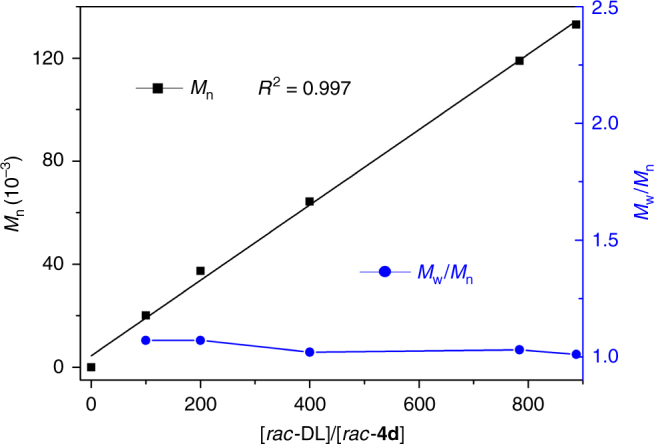
Degree of control over the molecular weight and dispersity in the ROP of *rac*-DL. Plots of *M*_n_ and *Đ* values of isotactic P3HB produced by racemic catalyst **4d** at varied [*rac*-DL]/[*rac*-**4d**] ratios. The runs at 800/1 and 1200/1 ratios under the same conditions (0.8 mL DCM, 60 min) did not achieve quantitative conversions; thus, the ratios used for the plot were adjusted by their conversions (98% and 74%, respectively)

Having identified catalyst **4d** being the best catalyst of this series, we further examined the ability of this catalyst to control the molecular weight so that practically useful high-molecular-weight isotactic P3HB with *M*_n_ > 10^5^ g mol^−1^ could be synthesized. To this end, we varied the [*rac*-DL]/[**4d**] feed ratio from 100/1 to 1200/1 and found that the molecular weight of the resulting P3HB increased linearly (*R*^2^ = 0.997, Fig. 4) from medium *M*_n_ = 2.01 × 10^4^ g mol^−1^ (*Đ* = 1.07) to high *M*_n_ = 1.54 × 10^5^ g mol^−1^ (*Đ* = 1.01), while all the *Đ* values remained low in a narrow range from 1.01 to 1.07 (runs 9–13, Table 1). These observations, coupled with the calculated high initiation efficiencies of >86% for all the polymerization runs by catalyst **4d**, pointed to a well-controlled ROP of *rac*-DL.

### Thermal properties

**Fig. 5:**
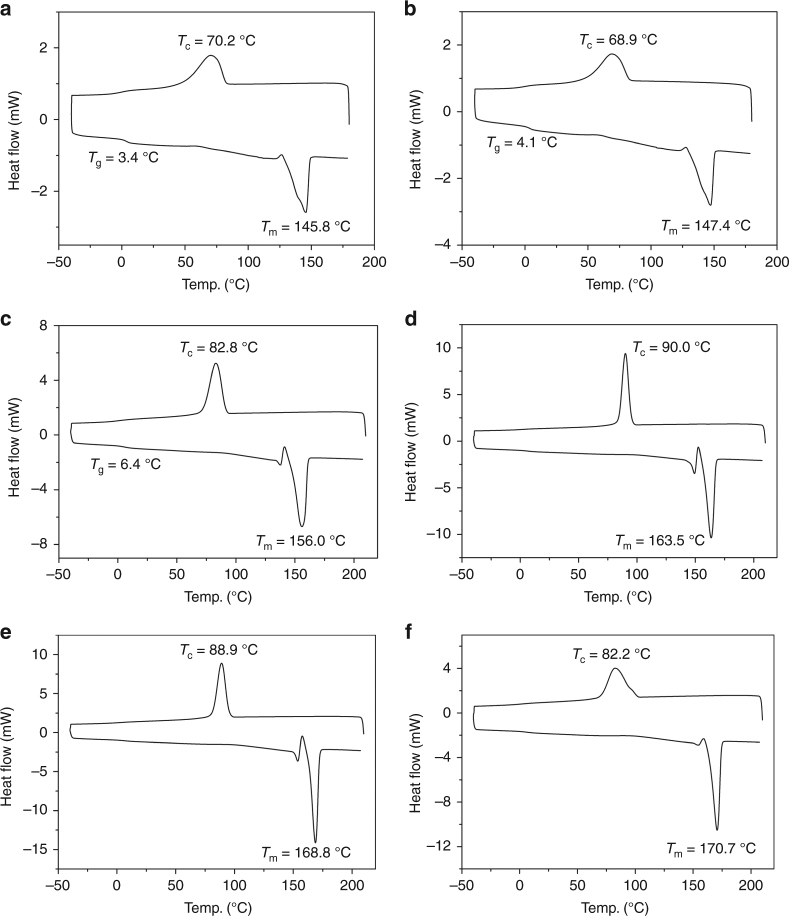
Thermal properties of P3HB derived from the ROP of *rac*-DL. **a** [*rac*-DL]/[**4a**] = 200/1 (∆*H*_f_ = 40.9 J g^−1^). **b** [*rac*-DL]/[**4b**] = 200/1 (∆*H*_f_ = 47.0 J g^−1^). **c** [*rac*-DL]/[**4c**] = 200/1 (∆*H*_f_ = 56.5 J g^−1^). **d** [*rac*-DL]/[**4d**] = 200/1 (∆*H*_f_ = 79.3 J g^−1^). **e** [*rac*-DL]/[**4d**] = 400/1 (∆*H*_f_ = 80.4 J g^−1^). **f** [*rac*-DL]/[**4d**] = 1200/1 (∆*H*_f_ = 78.6 J g^−1^). Crystallization temperature (*T*_c_) and melting-transition temperature (*T*_m_) taken from the cooling and second heating scans, respectively

DSC curves of the isotactic P3HB materials produced by the ROP of *rac*-DL with catalysts **4a**–**4d** under identical conditions ([*rac*-DL]/[**4**] = 200/1, DCM, RT, 20 min, 100% conversion) are compared in Fig. 5. Consistent with the gradual increase of the isotacticity of P3HB on going from **4a** to **4d**, the *T*_m_ value was observed to increase from 146 (**4a**) to 147 (**4b**), 156 (**4c**), and 164 °C (**4d**). Noteworthy also is the steadily enhanced heat of fusion (∆*H*_f_) from **4a** to **4d**: 40.9, 47.0, 56.5, and 79.3 J g^−1^, which corresponds to an increase in the P3HB crystallinity from 28 to 32, 39, and 54%, relative to the estimated ∆*H*_f_^0^ value (146 J g^−1^) for an infinite crystal of 100% crystalline P3HB (Supplementary Note 1)^72^. Furthermore, the *T*_m_ value of the perfectly isotactic P3HB produced by catalyst **4d** depends somewhat on the polymer *M*_n_: when *M*_n_ was enhanced from 2.01 × 10^4^ to 3.74 × 10^4^, 6.43 × 10^4^, 1.19 × 10^5^, and 1.54 × 10^5^ g mol^−1^ (runs 9–13, Table 1), *T*_m_ increased accordingly from 161 to 164, 169, 170, and 171 °C. These are perfectly isotactic, highly crystalline materials, with high ∆*H*_f_ values of ~ 80 J g^−1^ (Fig. 5, Supplementary Fig. 40).

**Fig. 6:**
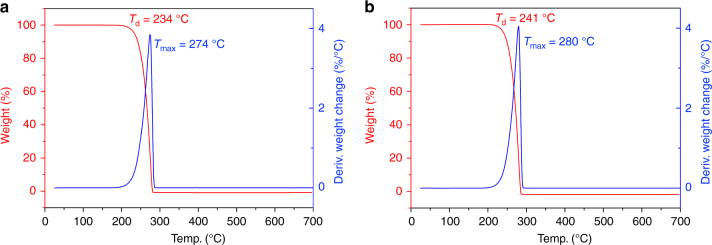
Thermal stability of P3HB derived from the ROP of *rac*-DL. **a** TGA and DTG of P3HB produced by catalyst **4a**, *M*_n_ = 3.20 × 10^4^ g mol^−1^, *Đ* = 1.03, [*mm*] = 89%. **b** TGA and DTG of P3HB produced by catalyst **4d**, *M*_n_ = 1.19 × 10^5^ g mol^−1^, *Đ* = 1.03, [*mm*] > 99%

Thermal degradation profiles of the selected isotactic P3HB samples derived from the ROP of *rac*-DL by racemic  catalysts **4a** and **4d** were examined by thermal gravimetric analysis (TGA). As can be seen from the TGA and derivative thermogravimetry (DTG) curves (Fig. 6), the P3HB produced by **4a** (*M*_n_ = 3.20 × 10^4^ g mol^−1^, *Đ* = 1.03, [*mm*] = 89%) exhibited a decomposition temperature (*T*_d_) (defined by the temperature of 5% weight loss in the TGA curve) of 234 °C and a maximum rate decomposition temperature (*T*_max_) of 274 °C, which put the *T*_d_ ~ 90 °C above its *T*_m_, proving a window for melt processing. A P3HB sample produced by **4d** with a similar *M*_n_ (3.74 × 10^4^ g mol^−1^) but a much higher isotacticity of [*mm*] > 99% exhibited a somewhat higher *T*_d_ of 239 °C and *T*_max_ of 281 °C (Supplementary Fig. 41). Keeping the tacticity the same ([*mm*] > 99%) while increasing the molecular weight to *M*_n_ = 1.19 × 10^5^ g mol^−1^ enhanced the *T*_d_ only slightly to 241 °C (Fig. 6).

### Kinetic resolution and mechanistic considerations

The above results demonstrated that the polymerization of *rac*-DL by *rac*-**4d** produced P3HB with essentially perfect isotacticity. However, these exciting results also brought about three important fundamental questions: (1) Can enantiomeric catalysts (*R,R*)-**4d** and (*S,S*)-**4d** kinetically resolve racemic monomer *rac*-DL? (2) What is the stereocontrol mechanism—enantiomorphic-site control or chain-end control? (3) What is the isotactic polymer stereomicrostructure—1:1 mixture of poly[(*R,R*)-DL] and poly[(*S,S*)-DL], stereoblock of poly(*rac*-DL), or tapered block copolymer poly[(*R,R*)-DL]-*co*-[(*S,S*)-DL]?

To address these questions, the corresponding enantiomeric catalysts (*R,R*)-**4d** and (*S,S*)-**4d** have been successfully synthesized and subsequently employed to perform kinetic resolution polymerization of *rac*-DL with both enantiomeric catalysts (See Supplementary Information for details, Supplementary Figs. 23–29), the results of which are summarized in Table 2. In sharp contrast to the polymerization by *rac*-**4d** with a [*rac*-DL]/[*rac*-**4d**] ratio of 400 or 800 that achieved a quantitative monomer conversion after 20 min, the same polymerization by (*R,R*)-**4d** or (*S,S*)-**4d** achieved a conversion of ~50% (runs 1–2 and 4–5, Table 2), after which no further monomer conversion can be achieved, even after extended times (6 h), indicative of exclusive catalyst site selectivity for the ROP of one particular enantiomer of the monomer. Characterizations of the ~50% unreacted, pure monomer showed to exhibit identical NMR spectra to that of *rac*-DL (Supplementary Fig. 42); the specific rotation of the unreacted monomer produced by (*R,R*)-**4d** was measured to be from −94.8° to −94.5° (runs 1–2, Table 2), which agrees well with the reported value of (*R,R*)-DL^57^, while the specific rotation of the unreacted monomer by (*S,S*)-**4d** was from +93.7 to +94.6 (runs 4–5, Table 2), which is assigned to be (*S,S*)-DL accordingly. The enantiomeric excess (*e.e*.) of the unreacted monomer was determined by chiral HPLC coupled with a Chiralcel OD-H column to be >99% *e.e*. (Supplementary Figs. 55, 56 and 58, 59) for all the runs with conversion at ~50% (runs 1–2 and 4–5, Table 2). It is noted here that the [*rac*-DL]/[**4d**] = 1600 runs achieved 43–44% conversion after 60 min (runs 3 and 6); accordingly, the *e.e*.’s of the unreacted monomer was only 80–85%, respectively (Supplementary Figs. 57 and 60). Overall, these results indicate that (*R,R*)-**4d** exhibits exclusive selectivity for the ROP of (*S,S*)-DL, while (*S,S*)-**4d** polymerizes (*R,R*)-DL exclusively. Based on the quantitative *e.e*.’s (>99%) for the optically resolved, unreacted monomer, the stereoselectivity factor *s* for both enantiomeric catalysts was calculated to be >10^3^. These profound results also indicated an enantiomorphic-site control mechanism for the stereoselective ROP process.

**Table 2:** Results of *rac*-DL polymerization by yttrium catalysts (*R,R*)- and (*S,S*)-**4d**/BnOH

Run	Catalyst	[*rac*-DL]/[**4d**]	Time (min)	Conv. (%)	*M*_n_ (kg mol^−1^)	*Ð* (*M*_w_/*M*_n_)	[*mm*] (%)	*T*_m_^a^ (°C)	[*α*]_D_^23^ (°)^b^	*e.e*.^c^ (%)
1	(*R,R*)-**4d**	400/1	20	~50	43.0	1.07	>99	172	−94.8	>99
2	(*R,R*)-**4d**	800/1	20	~50	74.8	1.05	>99	172	−94.5	>99
3	(*R,R*)-**4d**	1600/1	60	43	118	1.09	>99	175	−73.6	80
4	(*S,S*)-**4d**	400/1	20	~50	42.9	1.08	>99	172	+93.7	>99
5	(*S,S*)-**4d**	800/1	20	~50	72.1	1.04	>99	172	+94.6	>99
6	(*S,S*)-**4d**	1600/1	60	44	113	1.07	>99	175	+76.1	85

Conditions: *rac*-DL = 0.241 g (1.40 mmol), [*rac*-DL] = 1.0 M in DCM (1.4 mL); room temperature. See footnotes in Table 1 for other explanations

^a^*T*_m_ measured by DSC with the cooling and second heating rate of 10 °C min^−1^

^b^Specific rotation ([*α*]_D_^23^) of the unreacted monomer (DL) in chloroform

^c^Enantiomeric excess (*e.e*.) determined by chiral HPLC

**Fig. 7:**
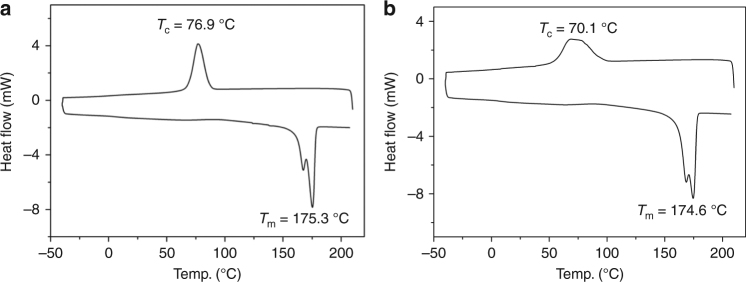
DSC curves of poly[(*R*)-3HB] materials. **a** Produced via chemical ROP of *rac*-DL by (*S,S*)-[**4d**]. **b** Commercial natural poly[(*R*)-3-hydroxybutyric acid] purchased from Sigma-Aldrich. *T*_c_ and *T*_m_ values taken from the cooling and second heating scans, respectively

This essentially perfect stereoselectivity enabled the synthesis of enantiomeric poly[(*R,R*)-DL] and poly[(*S,S*)-DL] using (*S,S*)-**4d** and (*R,R*)-**4d**, respectively. The *T*_m_ values (Table 2, Supplementary Fig. 43) of these enantiopure polymers are somewhat higher (by 3–5 °C) than those of isotactic poly(*rac*-DL) with similar molecular weights, prepared by *rac*-**4d**, and also higher (by 5–7 °C) than that of 1:1 mixture of poly[(*R,R*)-DL] and poly[(*S,S*)-DL] (Supplementary Figs. 44–46), indicating no stereocomplexation between two enantiomeric P3HB chains. These enantiopure polymers also exhibit high ∆*H*_f_ values in the range of 79–88 J g^−1^, comparable to that of natural poly[(*R*)-3HB]. The highest *T*_m_ of 175 °C was achieved by the enantiomeric polymers with *M*_n_ = 113–118 kg mol^−1^ (runs 3 and 6, Table 2), which, along with the heat of fusion value, is essentially identical to that of the commercial natural poly[(*R*)-3HB] (Fig. 7).

**Fig. 8:**
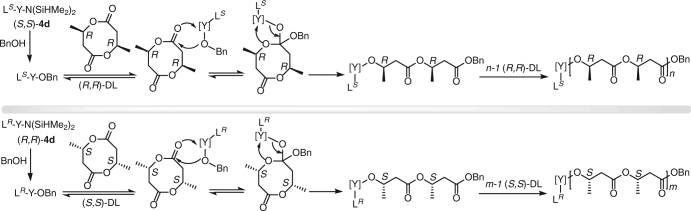
Proposed mechanism. Chemical scheme of the proposed catalyst-site controlled coordination-insertion mechanism for the ROP of *rac*-DL by *rac*-**4d**

To gain further insight into the stereomicrostructure of the highly isotactic P3HB produced by *rac*-**4d**, the polymers produced at different times and [*rac*-DL]/[*rac*-**4d**] ratios were analyzed by matrix-assisted laser desorption/ionization time-of-flight mass spectroscopy (MALDI-TOF MS). When a low ratio of 20/1 was used with a longer reaction time (20 min), the mass spectra showed the pronounced transesterification side reaction, as evidenced by the appearances of molecular ion peaks with the spacing between the neighboring peaks being that of the half molar mass of the repeat unit, DL (*m*/*z* = 172.07) (Supplementary Fig. 47), which also resulted in a higher dispersity (*Ð* = 1.20) and lower isotacticity (*P*_m_ = 0.96). Under these conditions, the microstructure of the resultant isotactic P3HB is a stereoblock polymer, poly[(*R*)-3HB]-*b*-poly[(*S*)-3HB], as a result of transesterification after the reaction reached full conversion (Supplementary Fig. 50). Consistent with this reasoning, when the polymerization was quenched after 30 s (at which time the full conversion was also achieved), the dispersity of the resulting polymer decreased to *Ð* = 1.09, and its mass spectrum displayed nearly exclusive molecular ion peaks with the spacing between the neighboring peaks being that of *rac*-DL, with just minor peaks with extremely low intensity for the odd 3-HB units (Supplementary Fig. 51). These results indicate that the transesterification side reaction occurred when the reaction reached full conversion and thus can be essentially shut down by reducing the polymerization time. In this case, the resulting polymer is predominately a mixture of poly[(*R*)-3HB] and poly[(*S*)-3HB], with only a trace amount of stereoblock polymer, poly[(*R*)-3HB]-*b*-poly[(*S*)-3HB]. Obviously, the transesterification side reaction can also be shut down by reducing the catalyst amount in feed with the same reaction time, giving the polymers with very low dispersity indices (*Ð* = 1.01–1.03) at higher [*rac*-DL]/[*rac*-**4d**] feeding ratios. Furthermore, this transesterification side reaction can be shut down by the use of enantiomeric pure catalysts (Supplementary Figs. 52, 53). Overall, the polymerization of *rac*-DL by *rac*-**4d**, when stopped at full conversion (i.e., no transesterification side reactions), produces a mixture of poly[(*R*)-3HB] and poly[(*S*)-3HB] (Fig. 8), due to its exclusive (*R,R*)-**4d**/(*S,S*)-DL and (*S,S*)-**4d**/(*R,R*)-DL catalyst/monomer selectivity as demonstrated by the kinetic resolution results.

## Discussion

The approach via the ROP of the bio-sourced *rac*-DL has successfully addressed the 50+-year challenge in the chemical synthesis of bacterial P3HB. This success was enabled by the specifically designed stereoselective molecular catalyst and its steric interplay with the monomer structure, thereby producing the desired P3HB material with perfect isotacticity ([*mm*] > 99%), high crystallinity, and melting temperature (*T*_m_ = 171 °C), as well as high molecular weight and low dispersity (*M*_n_ = 154 kg mol^−1^, *Đ* = 1.01). This novel ROP of *rac*-DL also exhibits a high polymerization rate and efficiency. The resulting highly isotactic P3HB by *rac*-**4d** is shown to be the mixture of poly[(*R*)-3HB] and poly[(*S*)-3HB] when the polymerization is stopped at full conversion. Thanks to the exclusive (*R,R*)-**4d**/(*S,S*)-DL and (*S,S*)-**4d**/(*R,R*)-DL catalyst/monomer selectivity, kinetic resolution polymerization of *rac*-DL with enantiomeric catalysts automatically ceases at 50% conversion and yields enantiopure (*R,R*)-DL and (*S,S*)-DL with >99% *e.e*. and the corresponding poly[(*S*)-3HB] and poly[(*R*)-3HB] with high *T*_m_ = 175 °C and crystallinity, which is essentially identical to that of the commercial natural poly[(*R*)-3HB].

The tunability of the catalyst structure allows a rapid entry to P3HB materials with various tacticities (thus tunable thermal and mechanical properties) and predicted molecular weights with low dispersity indices (vs. a typical *Đ* value of ~2.0 for bacterial P3HB). In addition, the molecular catalysts should allow copolymerization of *rac*-DL with other monomers to produce 3HB-based copolymers. Altogether, this ROP of DL represents a paradigm shift in the chemical synthesis of P3HB and opens up a plethora of opportunities for discovering new catalysts, materials, and processes in the ROP of *rac*-DL and other diastereomers of 3HB cyclic dimers or trimers, and so on.

## Methods

### Materials and instruments

All syntheses and manipulations of air- and moisture-sensitive chemicals and materials were carried out in flamed Schlenk-type glassware on a dual-manifold Schlenk line or in an inert gas (Ar or N_2_)-filled glovebox. NMR-scale reactions were conducted in Teflon-valve-sealed J. Young-type NMR tubes. HPLC-grade organic solvents were first sparged extensively with nitrogen during filling 20 L solvent reservoirs and then dried by passage through activated alumina (for DCM) followed by passage through Q-5 supported copper catalyst (for toluene and hexanes) stainless steel columns. Benzene-*d*_6_ was dried over sodium/potassium alloy and filtered, whereas CD_2_Cl_2_ and CDCl_3_ were dried over CaH_2_, vacuum-distilled and stored over activated Davison 4 Å molecular sieves. HPLC chromatograms were obtained on an Agilent 1100 series system using a Chiralcel OD-H column with hexanes/isopropanol (80/20) as the eluent at a flow rate of 1.0 mL min^−1^. Optical rotations were measured with an Autopol-III automatic polarimeter. NMR spectra were recorded on a Varian Inova or Bruker AV-III 400 MHz spectrometer (400 MHz, ^1^H; 100 MHz, ^13^C). Chemical shifts for ^1^H and ^13^C spectra were referenced to internal solvent resonances and are reported as parts per million relative to SiMe_4_. Elemental analyses were performed by Robertson Microlit Laboratories, Madison, NJ.

Tri[*N*,*N*-bis(trimethylsilyl)amide] lanthanum(III) La[N(SiMe_3_)_2_]_3_ (**1**), yttrium chloride YCl_3_, and (trimethylsilyl)methyllithium (SiMe_3_)_2_CH_2_Li solution in pentane were purchased from Sigma-Aldrich Chemical Co. and used as received. 2,2-Diphenylethanol was purchased from Aldrich Chemical Co., which were purified by sublimation twice. Isopropanol and benzyl alcohol were purchased from Fisher Scientific Co. and Alfa Aesar Chemical Co., respectively, which were purified by distillation over CaH_2_ and stored over activated Davison 4 Å molecular sieves. Dimethyl succinate, sodium methoxide, and 3-chloroperoxybenzoic acid (mCPBA, 70–75%) were purchased from Fisher Scientific Co. and used as received. Iodomethane was purchased from Alfa Aesar Chemical Co. and used as received.

### General polymerization procedures

Polymerizations were performed in 5.5 mL glass reactors inside the inert glovebox at RT. The reactor was charged with a predetermined amount of catalyst and/or initiator and solvent (as specified in the polymerization tables) in a glovebox. The mixture was stirred at RT for 10 min, and the polymerization was initiated by rapid addition of *rac*-DL solution. After a desired time period, the polymerization was immediately quenched by addition of 0.5 mL of benzoic acid/chloroform (10 mg mL^−1^) and a 0.02 mL of aliquot was taken from the reaction mixture and prepared for ^1^H NMR analysis to obtain the percent monomer conversion data. The quenched mixture was then precipitated into 50 mL of cold methanol while stirring, filtered, washed with cold methanol to remove any unreacted monomer, and dried in a vacuum oven at RT overnight to a constant weight.

### Procedures for kinetic resolution of *rac*-DL by enantiomeric Y complexes (*R,R*)-**4d** and (*S,S*)-**4d**

*rac*-DL was polymerized by (*R,R*)-**4d** or (*S,S*)-**4d** according to the above general procedure. The conversion of monomer was ~50% with the [*rac*-DL]/[(*R,R*)-**4d**] molar ratio of 400 or 800 after 20 min, under which the conversion was 100% with *rac*-**4d** as the catalyst. The polymerization was then quenched with benzoic acid/DCM (caution: avoid using nucleophiles such as methanol or acidified methanol as they react with the unreacted monomer, see Supplementary Figs. 30, 31). The solvent was evaporated, and the solid residue was sublimated at 40–50 °C under vacuum to recover the pure unreacted monomer. The residue in the sublimator was dissolved in about 2 mL DCM, precipitated into 60 mL MeOH, filtrated, washed, and dried in vacuum to recover the polymer. The recovered unreacted monomer was analyzed by a chiral HPLC system to measure *e.e*. values, and the stereoselectivity factor *s* was calculated from Eq. (1):

1 $$S = \frac{{\ln \left[ {\left( {1 - c} \right)\left( {1 - ee_m} \right)} \right]}}{{\ln \left[ {\left( {1 - c} \right)\left( {1 + ee_m} \right)} \right]}}$$

where $$c = 1 - \frac{{\left[ S \right] + \left[ R \right]}}{{\left[ S \right]_0 + \left[ R \right]_0}},\,ee_m = \frac{{\left[ S \right] - \left[ R \right]}}{{\left[ S \right] + \left[ R \right]}}.$$

### Polymer characterizations

Measurements of polymer absolute weight-average molecular weight (*M*_w_), number-average molecular weight (*M*_n_), and molecular weight distributions or dispersity indices (*Đ* = *M*_w_/*M*_n_) were performed via gel-permeation chromatography (GPC). The GPC instrument consisted of an Agilent HPLC system equipped with one guard column and two PLgel 5 μm mixed-C gel permeation columns and coupled with a Wyatt DAWN HELEOS II multi (18)-angle light scattering detector and a Wyatt Optilab TrEX *d*RI detector; the analysis was performed at 40 °C using chloroform as the eluent at a flow rate of 1.0 mL min^−1^, using Wyatt ASTRA 7.1.2 molecular weight characterization software (some presentative GPC traces are shown in Supplementary Figs. 61–66). The refractive index increment (d*n*/d*c*) of P3HB was determined to be 0.0254 ± 0.0004 mL g^−1^, obtained by batch experiments using Wyatt Optilab TrEX *d*RI detector and calculated using ASTRA software. Polymer solutions were prepared in chloroform and injected into *d*RI detector by Harvard Apparatus pump 11 at a flow rate of 0.25 mL min^−1^. A series of known concentrations were injected and the change in refractive index was measured to obtain a plot of change in refractive index vs. change in concentration ranging from 0.4 to 4.0 mg mL^−1^. The slope from a linear fitting of the data was the d*n*/d*c* of the polymer.

The isolated low molecular weight samples were analyzed by MALDI-TOF MS; the experiment was performed on Microflex-LRF mass spectrometer (Bruker Daltonics, Billerica, MA) operated in positive ion, reflector mode using a Nd:YAG laser at 355 nm and 25 kV accelerating voltage. A thin layer of a 1% NaI solution was first deposited on the target plate, followed by 0.6 µL of both sample and matrix (dithranol in chloroform). External calibration was done using a peptide calibration mixture (4–6 peptides) on a spot adjacent to the sample. The raw data was processed in the FlexAnalysis software (version 3.4.7, Bruker Daltonics).

*T*_m_ and *T*_g_ were measured by DSC on an Auto Q20, TA Instrument. All *T*_m_ and *T*_g_ values were obtained from a second scan after the thermal history was removed from the first scan. The second heating rate was 10 °C min^−1^ and cooling rate was 10 °C min^−1^ unless indicated otherwise in the polymerization tables. *T*_d_, defined by the temperature of 5% weight loss, and *T*_max_ of the polymers were measured by TGA on a Q50 TGA Analyzer, TA Instrument. Polymer samples were heated from ambient temperatures to 700 °C at a heating rate of 10 °C min^−1^. Values of *T*_max_ were obtained from derivative (wt%/°C) vs. temperature (°C) plots, while *T*_d_ and *T*_onset_ values (initial and end temperatures) were obtained from wt% vs. temperature (°C) plots.

### Data availability

The authors declare that the data supporting the findings of this study are provided in the main article and the Supplementary Information.

## Electronic supplementary material

Supplementary Information

## References

[1] Anjum A (2016). Microbial production of polyhydroxyalkanoates (PHAs) and its copolymers: a review of recent advancements. Int. J. Biol. Macromol..

[2] Muhammadi S, Afzal M, Hameed S (2015). Bacterial polyhydroxyalkanoates-eco-friendly next generation plastic: production, biocompatibility, biodegradation, physical properties and applications. Green Chem. Lett. Rev..

[3] Laycock B, Halley P, Pratt S, Werker A, Lanta P (2013). The chemomechanical properties of microbial polyhydroxyalkanoates. Prog. Polym. Sci..

[4] Somleva MN, Peoples OP, Snell KD (2013). PHA bioplastics, biochemicals, and energy from crops. Plant Biotechnol. J..

[5] Taguchi, S., Iwata, T., Abe, H. & Doi, Y. in *Polymer Science: A Comprehensive Reference*, Vol. 9 (eds Matyjaszewski, K. & Moeller, M.) 157–182 (Elsiver B.V., Amsterdam, 2012).

[6] Chen, G.-Q. in *Plastics from Bacteria: Natural Functions and Applications*, Vol. 14 (ed Chen, G.-Q.) 17–37 (Springer-Verlag, Berlin, 2010).

[7] Chen GQ (2009). A microbial polyhydroxyalkanoates (PHA) based bio- and materials industry. Chem. Soc. Rev..

[8] Lenz RW, Marchessault RH (2005). Bacterial polyesters: biosynthesis, biodegradable plastics and biotechnology. Biomacromolecules.

[9] Sudesh K, Abe H, Doi Y (2000). Synthesis, structure and properties of polyhydroxyalkanoates: biological polyesters. Prog. Polym. Sci..

[10] Poirier Y, Nawrath C, Somerville C (1995). Production of polyhydroxyalkanoates, a family of biodegradable plastics and elastomers, in bacteria and plants. Nat. Biotechnol..

[11] Müller HM, Seebach D (1993). Poly(hydroxyalkanoates): a fifth class of physiologically important organic biopolymers?. Angew. Chem. Int. Ed. Engl..

[12] Doi Y (1990). Microbial Polyesters.

[13] Tong R (2017). New chemistry in functional aliphatic polyesters. Ind. Eng. Chem. Res..

[14] Hillmyer MA, Tolman WB (2014). Aliphatic polyester block polymers: renewable, degradable, and sustainable. Acc. Chem. Res..

[15] Coulembier O, Degée P, Hedrick JL, Dubois P (2006). From controlled ring-opening polymerization to biodegradable aliphatic polyester: especially poly(*β*-malic acid) derivatives. Prog. Polym. Sci..

[16] Mecking S (2004). Nature or petrochemistry?—biologically degradable materials. Angew. Chem. Int. Ed..

[17] Gross RA, Kalra B (2002). Biodegradable polymers for the environment. Science.

[18] Albertsson AC, Varma IK (2002). Aliphatic polyesters: synthesis, properties and applications. Adv. Polym. Sci..

[19] Middleton J, Tipton A (2000). Synthetic biodegradable polymers as orthopedic devices. Biomaterials.

[20] Mecerreyes D, Jérôme R, Dubois P (1999). Novel macromolecular architectures based on aliphatic polyesters: relevance of the “coordination-insertion” ring-opening polymerization. Adv. Polym. Sci..

[21] Carpentier JF (2015). Rare-earth complexes supported by tripodal tetradentate bis(phenolate) ligands: a privileged class of catalysts for ring-opening polymerization of cyclic esters. Organometallics.

[22] Jérôme C, Lecomte P (2012). Recent developments in ring-opening polymerization of lactones. Adv. Polym. Sci..

[23] Carpentier JF (2010). Discrete metal catalysts for stereoselective ring-opening polymerization of chiral racemic β-lactones. Macromol. Rapid Commun..

[24] Kiesewetter MK, Shin EJ, Hedrick JL, Waymouth RM (2010). Organocatalysis: opportunities and challenges for polymer synthesis. Macromolecules.

[25] Thomas CM (2010). Stereocontrolled ring-opening polymerization of cyclic esters: synthesis of new polyester microstructures. Chem. Soc. Rev..

[26] Kamber NE (2007). Organocatalytic ring-opening polymerization. Chem. Rev..

[27] Dechy-Cabaret O, Martin-Vaca B, Bourissou D (2004). Controlled ring-opening polymerization of lactide and glycolide. Chem. Rev..

[28] Okada M (2002). Chemical synthesis of biodegradable polymers. Prog. Polym. Sci..

[29] Jedlinski Z, Kurcok P, Lenz RW (1998). First facile synthesis of biomimetric poly-(*R*)-3-hydroxybutyrate via regioselective anionic polymerization of (*S*)-β-butyrolactone. Macromolecules.

[30] Iida M, Araki T, Teranishi K, Tani H (1977). Effect of substituents on stereospecific polymerization of β-alkyl- and β-chloroalkyl-β-propiolactones. Macromolecules.

[31] Teranishi K, Iida M, Araki T, Yamashita S, Tani H (1974). Stereospecific polymerization of β-alkyl-β-propiolactone. Macromolecules.

[32] Agostini DE, Undo JB, Shelton JR (1971). Synthesis and characterization of poly-β-hydroxybutyrate. I. Synthesis of crystalline DL-poly-β-hydroxybutyrate from DL-β-butyrolactone. J. Polym. Sci. Part A1.

[33] Inoue S, Tomoi S, Tsuruta T, Furukawa J (1961). Organometallic-catalyzed polymerization of propiolactone. Makromol. Chem..

[34] Moore T, Adhikari R, Gunatillake P (2005). Chemosynthesis of bioresorbable poly*(γ*-butyrolactone) by ring-opening polymerisation: a review. Biomaterials.

[35] Martin DP, Williams SF (2003). Medical applications of poly-4-hydroxybutyrate: a strong flexible absorbable biomaterial. Biochem. Eng. J..

[36] Hong M, Chen EYX (2016). Completely recyclable biopolymers with linear and cyclic topologies *via* ring-opening polymerization of *γ*-butyrolactone. Nat. Chem..

[37] Wu B, Lenz RW (1998). Stereoregular polymerization of [*R*,*S*]-3-butyrolactone catalyzed by alumoxane–monomer adducts. Macromolecules.

[38] Jaimes C (1998). Structure and morphology of poly([*R*,*S*]-β-butyrolactone) synthesized from aluminoxane catalyst. Eur. Polym. J..

[39] Bloembergen S, Holden DA, Bluhm TL, Hamer GK, Marchessault RH (1989). Stereoregularity in synthetic *β*-hydroxybutyrate and *β*-hydroxyvalerate homopolyesters. Macromolecules.

[40] Borgne AL, Spassky N (1989). Stereoselective polymerization of β-butyrolactone. Polymer (Guildf.).

[41] Takeichi T, Hieda Y, Takayama Y (1998). Asymmetric selective polymerization of β-butyrolactone catalyzed by optically active cobalt complex/triethylaluminum system. Polym. J..

[42] Rieth LR, Moore DR, Lobkovsky EB, Coates GW (2002). Single-site β-diiminate zinc catalysts for the ring-opening polymerization of β-butyrolactone and β-valerolactone to poly(3-hydroxyalkanoates). J. Am. Chem. Soc..

[43] Zintl M (2008). Variably isotactic poly(hydroxybutyrate) from racemic β-butyrolactone: microstructure control by achiral chromium(III) salophen complexes. Angew. Chem. Int. Ed..

[44] Ajellal N (2010). Polymerization of racemic β-butyrolactone using supported catalysts: a simple access to isotactic polymers. Chem. Commun..

[45] Kricheldorf HR, Eggerstedt S (1997). Polylactones. 41. Polymerization of β-D,L-butyrolactone with dialkyltinoxides as initiators. Macromolecules.

[46] Kemnitzer JE, McCarthy SP, Gross RA (1993). Syndiospecific ring-opening polymerization of β-butyrolactone to form predominantly syndiotactic poly(β-hydroxybutyrate) using tin(IV) catalysts. Macromolecules.

[47] Bouyahyi M, Ajellal N, Kirillov E, Thomas CM, Carpentier JF (2011). Exploring electronic versus steric effects in stereoselective ring-opening polymerization of lactide and β-butyrolactone with amino-alkoxybis(phenolate)–yttrium complexes. Chem. – Eur. J..

[48] Ajellal N (2009). Syndiotactic-enriched poly(3-hydroxybutyrate)s via stereoselective ring-opening polymerization of racemic *β*-butyrolactone with discrete yttrium catalysts. Macromolecules.

[49] Amgoune A, Thomas CM, Ilinca S, Roisnel T, Carpentier JF (2006). Highly active, productive, and syndiospecific yttrium initiators for the polymerization of racemic *β*-butyrolactone. Angew. Chem. Int. Ed..

[50] Amgoune A, Thomas CM, Roisnel T, Carpentier JF (2006). Ring-opening polymerization of lactide with group 3 metal complexes supported by dianionic alkoxy-amino-bisphenolate ligands: combining high activity, productivity, and selectivity. Chem. – Eur. J..

[51] Ligny R, Hänninen MM, Guillaume SM, Carpentier JF (2017). Highly syndiotactic or isotactic polyhydroxyalkanoates by ligand-controlled yttrium-catalyzed stereoselective ring-opening polymerization of functional racemic *β*-butyrolactone. Angew. Chem. Int. Ed..

[52] Drumright RE, Gruber PR, Henton DE (2000). Polylactic acid technology. Adv. Mater..

[53] Manso JA, Perez-Prior MT, Del Pilar Garcia-Santos M, Calle E, Casado J (2005). A kinetic approach to the alkylating potential of carcinogenic lactones. Chem. Res. Toxicol..

[54] Arcana M, Giani-Beaune O, Schue F, Amass W, Amass A (2000). Structure and morphology of poly(β-hydroxybutyrate) synthesized by ring-opening polymerization of racemic (*R,S*)-β-butyrolactone with distannoxane derivatives. Polym. Int..

[55] Tanahashi N, Doi Y (1991). Thermal properties and stereoregularity of poly(3-hydroxybutyrate) from optically active β-butyrolactone with a zinc-based catalyst. Macromolecules.

[56] Zhang Y, Gross RA, Lenz RW (1990). Stereochemistry of the ring-opening polymerization of (*S*)-β-butyrolactone. Macromolecules.

[57] Seebach D, Hoffmann T, Kühnle FNM, Kinkel JN, Schulte M (1995). Preparation, structure, and properties of all possible cyclic dimers (diolides) of 3-hydroxybutanoic acid. Helv. Chim. Acta.

[58] Seebach D, Hoffmann T, Kühnle FNM, Kinkel JN, Schulte M (1992). The triolide of (*R*)-3-hydroxybutyric acid—direct preparation from polyhydroxybutyrate and formation of a crown estercarbonyl complex with Na Ions. Angew. Chem. Int. Ed..

[59] Melchiors M, Keul H, Höcker H (1996). Depolymerization of poly[(*R*)-3-hydroxybutyrate] to cyclic oligomers and polymerization of the cyclic trimer: an example of thermodynamic recycling. Macromolecules.

[60] Melchiors M, Keul H, Höcker H (1994). Synthesis of highly isotactic poly[(*R*)-3-hydroxybutyrate] by ring-opening polymerization of (*R,R,R*)-4,8,12-trimethyl-l,5,9-trioxacyclododeca-2,6,10-trione. Macromol. Chem. Rapid Commun..

[61] Hong M, Tang X, Newell BS, Chen EYX (2017). 'Non-strained' *γ*-butyrolactone-based copolyesters: copolymerization characteristics and composition-dependent (thermal, eutectic, cocrystallization and degradation) properties. Macromolecules.

[62] Tang X (2016). The quest for converting biorenewable bifunctional *α*-methylene*-γ*-butyrolactone into degradable and recyclable polyester: controlling vinyl-addition/ring-opening/cross-linking pathways. J. Am. Chem. Soc..

[63] Liu, Q. et al. Cationic rare-earth metal SALEN complexes. *Dalton Trans*. **44**, 6170–6178 (2008). 10.1039/b808781d 18985250

[64] Lin MH, RajanBabu TV (2002). Ligand-assisted rate acceleration in transacylation by a yttrium**-**salen complex. Demonstration of a conceptually new strategy for metal-catalyzed kinetic resolution of alcohols. Org. Lett..

[65] DiCiccio AM, Longo JM, Rodríguez-Calero GG, Coates GW (2016). Development of highly active and regioselective catalysts for the copolymerization of epoxides with cyclic anhydrides: an unanticipated effect of electronic variation. J. Am. Chem. Soc..

[66] Char J (2016). Microstructurally controlled polymers of *rac*-lactide by lithium complexes. C. R. Chim..

[67] Chen HL, Dutta S, Huang PY, Lin CC (2012). Preparation and characterization of aluminum alkoxides coordinated on salen-type ligands: highly stereoselective ring-opening polymerization of *rac*-lactide. Organometallics.

[68] Kochnev AI, Oleynik II, Oleynik IV, Ivanchev SS, Tolstikov GA (2007). Synthesis of salicylaldehydes bearing bulky substituents in the positions 3 and 5. Russ. Chem. Bull. Int. Ed..

[69] Sanz M (2004). Monocyclopentadienyl bis(phenoxo-imino)zirconium complexes as precatalyst for olefin polymerization. Stereospecific methylation of an imino group with formation of a zirconium–amido bond. Organometallics.

[70] Larrow JF (1994). A practical method for the large-scale preparation of [*N,N’*-Bis(3,5-di-tert-butyl salicylidene)-1,2-cyclohexanediaminato2(-)]manganese (III) chloride, a highly enantioselective epoxidation catalyst. J. Org. Chem..

[71] Casiraghi, G., Casnati, G., Puglia, G., Sartori, G. & Terenghi, G. Selective reactions between phenols and formaldehyde. A novel route to salicylaldehydes. *J. Chem. Soc. Perkin Trans. 1* **0**, 1862–1865 (1980).

[72] Barham PJ, Keller A, Otun EL, Holmes PA (1984). Crystallization and morphology of a bacterial thermoplastic: poly-3-hydroxybutyrate. J. Mater. Sci..

