# Supplementary material for: Chemical synthesis of perfectly isotactic and high melting bacterial poly(3-hydroxybutyrate) from bio-sourced racemic cyclic diolide
Source: Nat Commun. 2018 Jun 11;9:2345. doi: 10.1038/s41467-018-04734-3 (PMC5995816; doi:10.1038/s41467-018-04734-3)
Supplement: Supplementary file 1 — Supplementary Information [file 41467_2018_4734_MOESM1_ESM.pdf]

Supplementary Information for

Chemical Synthesis of Perfectly Isotactic and High Melting Bacterial Poly(3-hydroxybutyrate) from Bio-Sourced Racemic Cyclic Diolide

Tang *et al.*

Supplementary Methods

The following compounds, ligands, and complexes were prepared according to their respective literature procedures: $Y[N(SiHMe_2)_2]_3(THF)_2$[1,2] and yttrium complexes **2** and **3** supported by the tetradentate, dianionic alkoxy-amino-bis(phenolate) [O^-,N,O,O^-] ligands.[3] The monomer, racemic eight-membered cyclic diolide (*rac*-DL) was prepared according to the literature route[4,5] but the procedures were significantly modified and thus described in detail below; the monomer was purified by sublimation twice prior to polymerization runs.

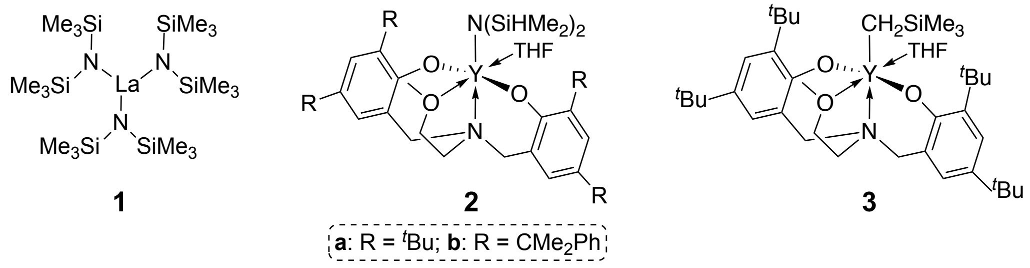

Supplementary Figure 1. Structures of complexes **1-3**.

Synthesis of Racemic Eight-membered Cyclic Diolide (*rac*-DL)

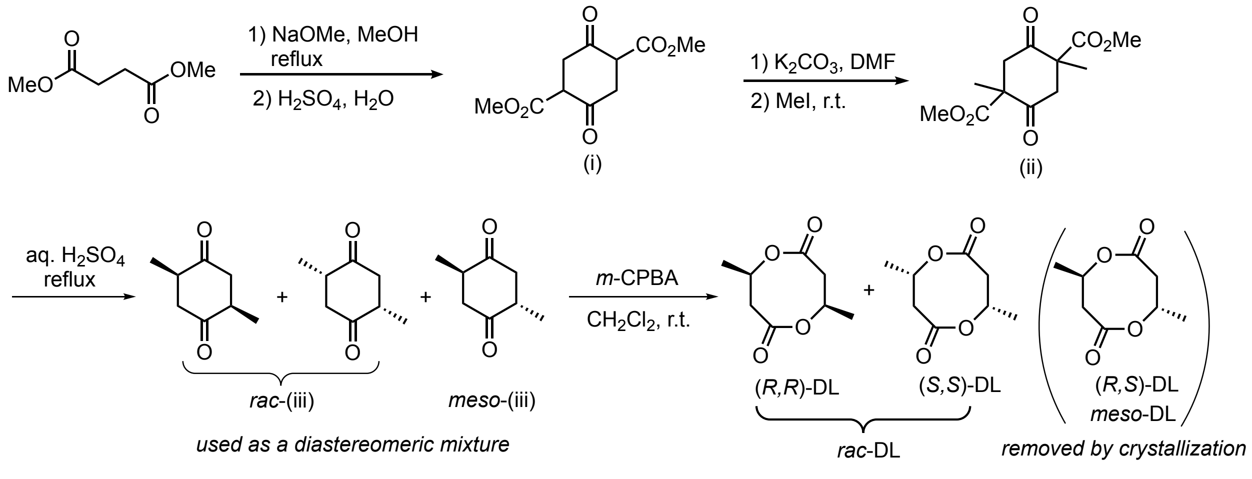

Supplementary Figure 2. Synthetic route for racemic eight-membered cyclic ciolide (*rac*-DL).

Dimethyl 2,5-dioxocyclohexane-1,4-dicarboxylate (i). A solution of sodium methoxide (185 mL, 5.4 M, 1.0 mol) was added to dimethyl succinate (73.1 g, 0.5 mol) in one portion, and the mixture was heated under reflux for 24 h. A thick pink-colored precipitate was then formed and remained throughout

the reaction. The methanol was removed using evaporator, a 2*N* sulfuric acid solution (500 mL) was added to the residue, and the mixture was stirred vigorously for 4 h. The solid was collected by filtration and washed several times with water. The air-dried product was a pale-buff powder, which was recrystallized from 300 mL ethyl acetate. The filtrate was chilled to give cream to pink-cream colored crystals of (i), 24.5 g (43%). ^{1}H NMR (400 MHz, $CDCl_3$): δ 12.12 (s, 1H, -C*H*-), 3.79 (s, 3H, $-CO_2CH_3$), 3.18 (s, 2H, $-CH_2-$).

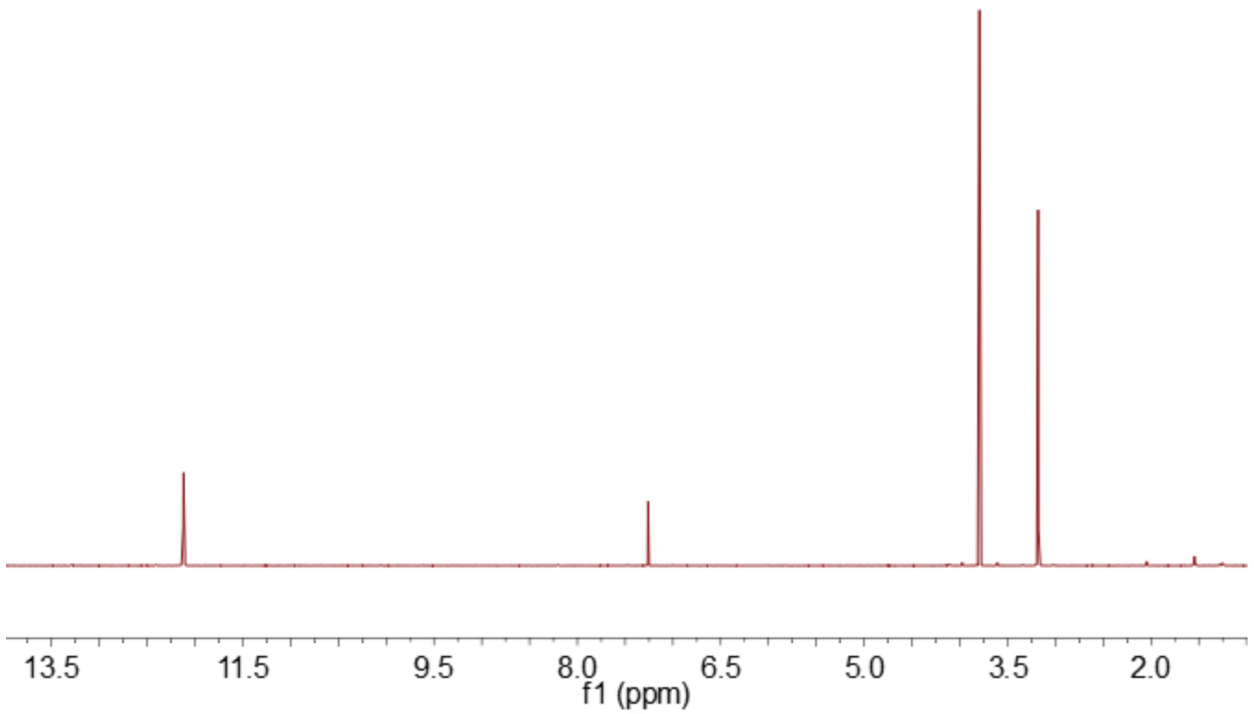

Supplementary Figure 3. ^{1}H NMR ($CDCl_3$) spectrum of dimethyl 2,5-dioxocyclohexane-1,4-dicarboxylate (i).

Dimethyl 1,4-dimethyl-2,5-dioxocyclohexane-1,4-dicarboxylate (ii). To a stirred suspension of K_2CO_3 (41.5 g, 0.3 mol) in 400 mL DMF under N_2 was added (i) (22.8 g, 0.1 mmol). After 15 min stirring at room temperature, MeI (56.8 g, 0.4 mmol) was added dropwise. After 15 h, the mixture was concentrated in vacuo, dissolved in 300 mL of H_2O, and extracted with CH_2Cl_2 (120 mL × 5). The combined organic layers were washed with 10% $Na_2S_2O_3$ solution, dried with anhydrous Na_2SO_4, and

evaporated. The residue was purified by column chromatography to give 20.8 g (81%) of (ii) as a 2:1 mixture of diastereoisomers. ^{1}H NMR (400 MHz, $CDCl_3$), main diastereoisomer: δ 3.72 (s, 6H, *Me*O); ν_A = 3.15, ν_B = 2.81 *(AB,* J_{AB} = 15.2, 4H, CH_2); 1.44 (s, 6H, *Me*); minor diastereoisomer: δ 3.74 (s, 6H, *Me*O); ν_A = 3.44, ν_B = 2.61 (*AB,* J_{AB} = 15.7, 4H, CH_2); 1.41 (s, 6H, *Me*).

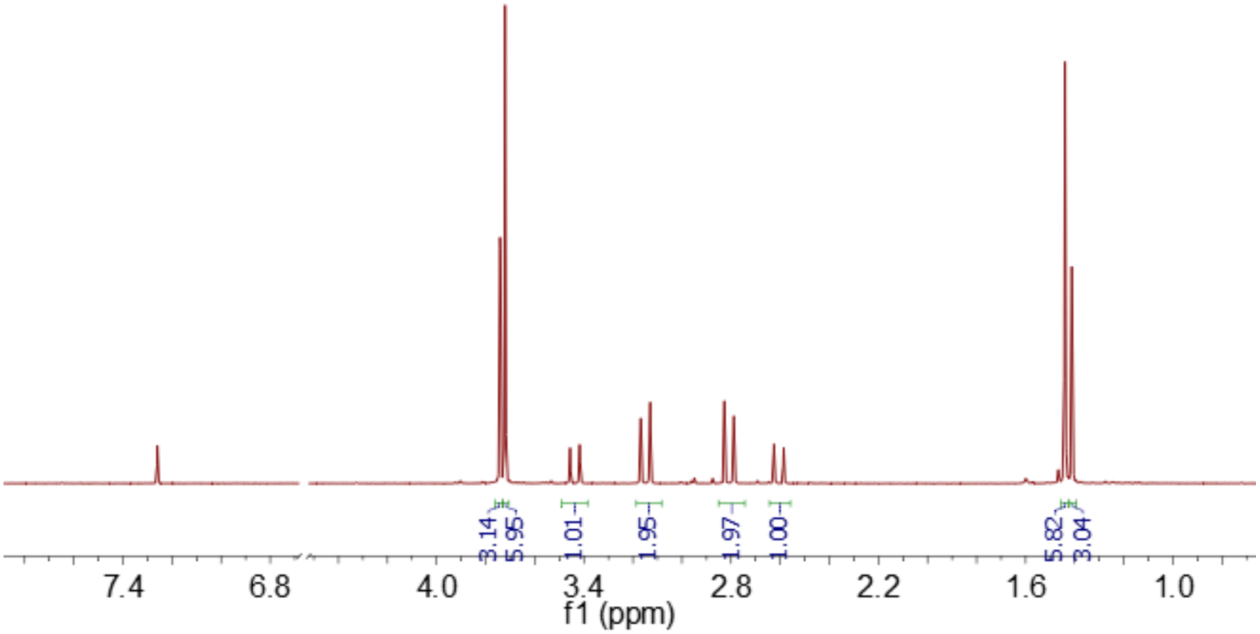

Supplementary Figure 4. ^{1}H NMR ($CDCl_3$) spectrum of dimethyl 1,4-dimethyl-2,5-dioxocyclohexane-1,4-dicarboxylate (ii)

Synthesis of 2,5-dimethylcyclohexane-1,4-dione (iii). To a stirred suspension of (ii) (20.5 g, 80 mmol) in 40 mL of conc. H_2SO_4 were added 3 mL of methanol and 45 g of crushed ice. After 15 min, the mixture was heated to 100 °C for an additional 2 h. The acidic solution was cooled to room temperature, neutralized with aq. NaOH (pH 6-7), and extracted with CH_2Cl_2 (150 mL × 3). The combined organic layers were dried with anhydrous Na_2SO_4, and evaporated. The residue was purified by column chromatography to afford 10.2 g (91%) of *rac*-(iii) (70%) and *meso*-(iii) (30%).

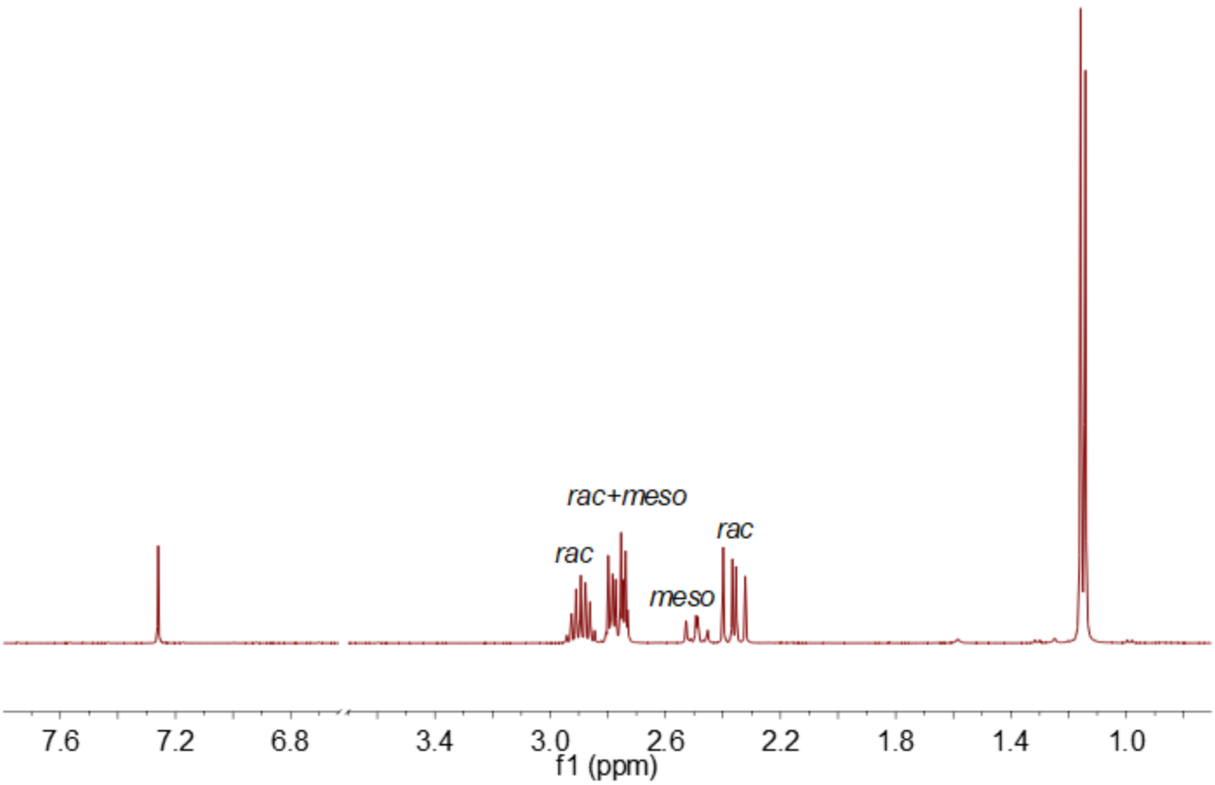

Supplementary Figure 5. ^{1}H NMR spectrum of 2,5-dimethylcyclohexane-1,4-dione (iii) in $CDCl_3$

4,8-Dimethyldioxocane-2,6-dione (*rac*-DL). To a solution of the mixture of *rac*-(iii) and *meso*-(iii) (10.0 g, 71 mmol) in 300 mL of CH_2Cl_2 was added *m*-CPBA (52.7 g, 70%, 213 mmol) in one portion. The pale-yellow solution was stirred at room temperature in the dark for 48 h. The obtained white suspension was diluted with 200 mL of CH_2Cl_2, washed saturated $NaHCO_3$ solution (200 mL × 3), which contained 5% $Na_2S_2O_3$, dried with anhydrous Na_2SO_4, and evaporated. After recrystallization of the residue (10.5 g) from hexanes/ethyl acetate (5/1) for 3~6 times, 5.1 g of pure *rac*-DL was obtained. ^{1}H NMR (400 MHz, $CDCl_3$): δ 5.35 – 5.23 (m, 2H, MeC*H*O-C=O), v_A = 2.65, v_B = 2.53, (*AB* of *ABX*, J_{AB} = 11.4, J_{AX} = 9.7, J_{BX} = 3.6, 4H, CH_2), 1.44 (d, J = 6.4 Hz, 6H, Me).

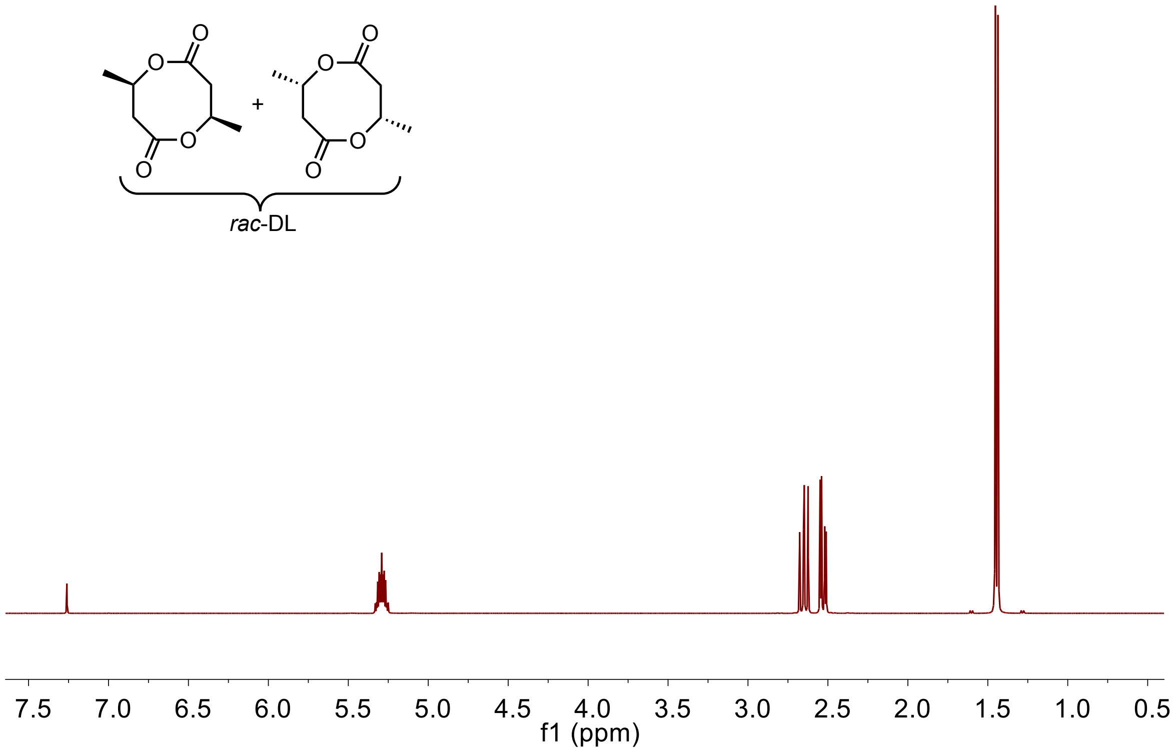

Supplementary Figure 6. ^{1}H NMR spectrum of *rac*-DL in $CDCl_3$.

Synthesis of Yttrium Complexes *rac*-4a-d and 4e

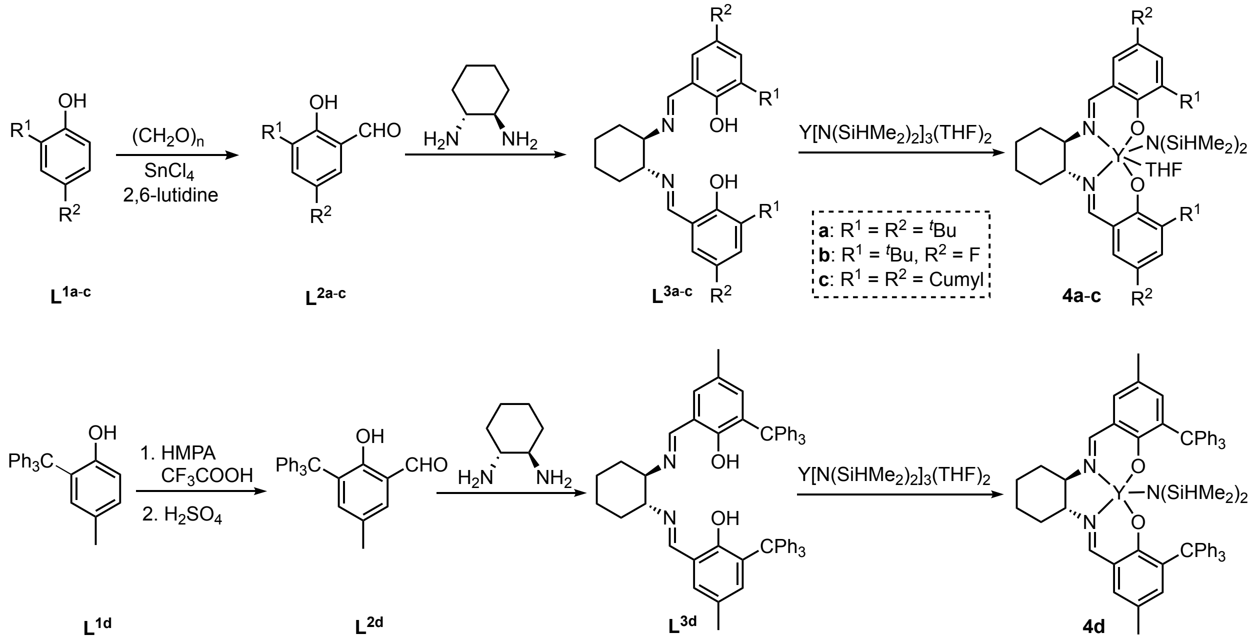

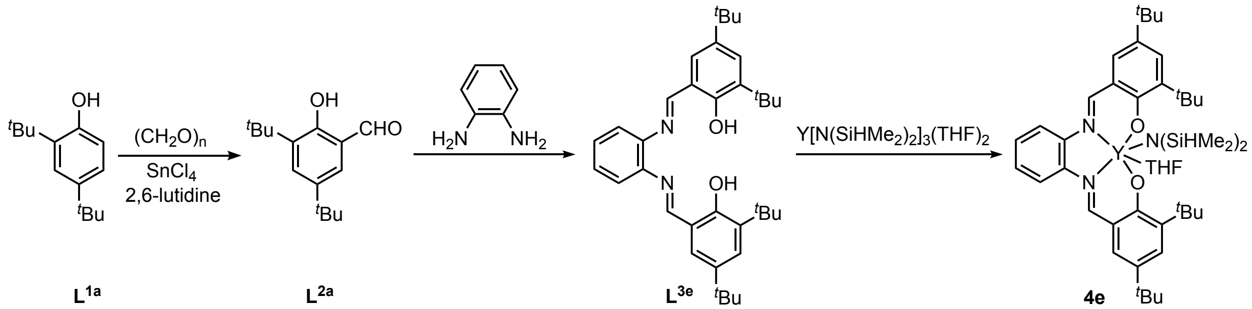

L^1a^ **L^2a^** **L^3e^** **4e**

Supplementary Figure 7. Synthesis of Yttrium Complexes *rac*-**4a-d** and **4e.**

Synthesis of Salicylaldehydes $\mathbf{L^{2a\text{-}d}}$

(1) 3,5-Bis(*tert*-butyl)salicylaldehyde $\mathbf{L^{2a}}$.[6] 2,4-Di-*tert*-butylphenol (30.45 g, 0.147 mol), 2,6-lutidine (6.9 mL, 0.059 mmol) and 100 mL anhydrous toluene were measured into a side-arm round-bottom flask under nitrogen. Tin(IV) chloride (1.72 mL, 14.7 mmol) was added slowly to the reaction flask. The mixture was stirred at room temperature for 30 min, and then paraformaldehyde (9.74 g, 0.325 mol) was added. The resulting yellowish solution was heated at 100 °C for 8 h, after which time TLC analysis indicated >99% consumption of the phenol. The mixture was allowed to cool to room temperature, and 600 mL water was added to the flask. The aqueous layer was acidified to approximately pH = 2 with 2 N HCl. The aqueous layer was extracted with diethyl ether, and the combined ether extracts were dried over anhydrous Na_2SO_4. The concentrated product was purified using column chromatography. Yield: 18.6 g (54%). ^{1}H NMR (400 MHz, $CDCl_3$): δ 11.64 (s, 1H, OH), 9.87 (s, 1H, CHO), 7.59 (d, J = 2.4 Hz, 1H, Ar-H), 7.35 (d, J = 2.4 Hz, 1H, Ar-H), 1.43 (s, 9H, ^{t}Bu), 1.33 (s, 9H, ^{t}Bu).

(2) 3-*tert*-Butyl-5-fluorosalicylaldehyde $\mathbf{L^{2b}}$. 2-*tert*-Butyl-4-fluorophenol $\mathbf{L^{1b}}$ was first prepared according to the literature procedure.[7] Next, $\mathbf{L^{2b}}$ was synthesized following the general procedure detailed for $\mathbf{L^{2a}}$, expect that $\mathbf{L^{1b}}$ was used instead of $\mathbf{L^{1a}}$. Yield: 43%. ^{1}H NMR (400 MHz, $CDCl_3$): δ 11.59 (s, 1H, OH), 9.82 (s, 1H,-CHO), 7.28 (dd, J = 10.3, 2.9 Hz, 1H, Ar-H), 7.07 (dd, J = 7.0, 3.1 Hz, 1H, Ar-H), 1.41 (s, 9H, ^{t}Bu).

(3) 3,5-Dicumylsalicylaldehyde $\mathbf{L^{2c}}$. 3,5-Dicumylsalicylaldehyde **2c** was synthesized according to the general procedure detailed for $\mathbf{L^{2a}}$, except that 2,4-dicumylphenol $\mathbf{L^{1c}}$ was used instead of 2,4-di-*tert*-butylphenol $\mathbf{L^{1a}}$.[8] Yield: 57%. ^{1}H NMR (400 MHz, $CDCl_3$): δ 11.25 (s, 1H, OH), 9.77 (s, 1H, CHO), 7.51 (d, J = 2.3 Hz, 1H, Ar-H), 7.38 – 7.09 (m, 11H, Ar-H), 1.73 (s, 6H, Me), 1.64 (s, 6H, Me).

(4) 3-Trityl-5-methylsalicylaldehyde $\mathbf{L^{2d}}$. 2-Trityl-4-methylphenol $\mathbf{L^{1d}}$ was prepared first according to the literature procedure.[8] Next, a mixture of $\mathbf{L^{1d}}$ (5.26 g, 15 mmol), hexamethylenetetraine (4.2 g, 30 mmol), and CF_3COOH (15 mL) was heated for 4 h at 115–125 °C, and then cooled down to 75–80 °C. H_2SO_4 (33% aq., 23 mL) was added to the reaction, and the resulting mixture was heated for 1-2 h at 125–130 °C. After cooling down to room temperature, ethyl acetate (40 mL) and water (50 mL) were added. The organic layer was separated and water was extracted with ethyl acetate (3 × 20 mL). The combined extracts were washed with water (70 mL) and brine (50 mL), separated, and dried over anhydrous Na_2SO_4. The product was purified by column chromatography. Yield: 4.50 g (79%). ^{1}H NMR (400 MHz, CDCl3): δ 11.12 (s, 1H, OH), 9.80 (s, 1H, CHO), 7.36 (d, J = 1.9 Hz, 1H, Ar-H), 7.29 (d, J = 1.5 Hz, 1H, Ar-H), 7.25 – 7.14 (m, 15H, Ar), 2.27 (s, 3H, Me).

Synthesis of Salen Ligands $\mathbf{L^{3a\text{-}e}}$

(1) Salcy ligand $\mathbf{L^{3a}}$.[9] A mixture of 3,5-bis(*tert*-butyl)salicylaldehyde $\mathbf{L^{2a}}$ (7.03 g, 30 mmol) and racemic *trans*-1,2-diaminocyclohexane (1.71 g, 15.0 mmol) was dissolved in methanol (80 mL), and about 0.2 mL formic acid was added to the solution. The reaction was then heated to reflux for 6 h. Upon cooling, the yellow precipitate was collected by filtration and dried under vacuum. Yield: 6.4 g (78%). ^{1}H NMR (400 MHz, $CDCl_3$): δ 13.71 (s, 2H, OH), 8.30 (s, 2H, N=C*H*), 7.28 (d, J = 2.8 Hz, 2H, Ar-H), 6.98 (d, J = 2.2 Hz, 2H, Ar-H), 3.32 (m, 2H, NC*H*), 1.94 (m, 2H, Cy-*H*), 1.86 (m, 2H, Cy-*H*), 1.75 (m, 2H, Cy-*H*), 1.47 (m, 2H, Cy-*H*), 1.41 (s, 9H, ^{t}Bu), 1.23 (s, 9H, ^{t}Bu).

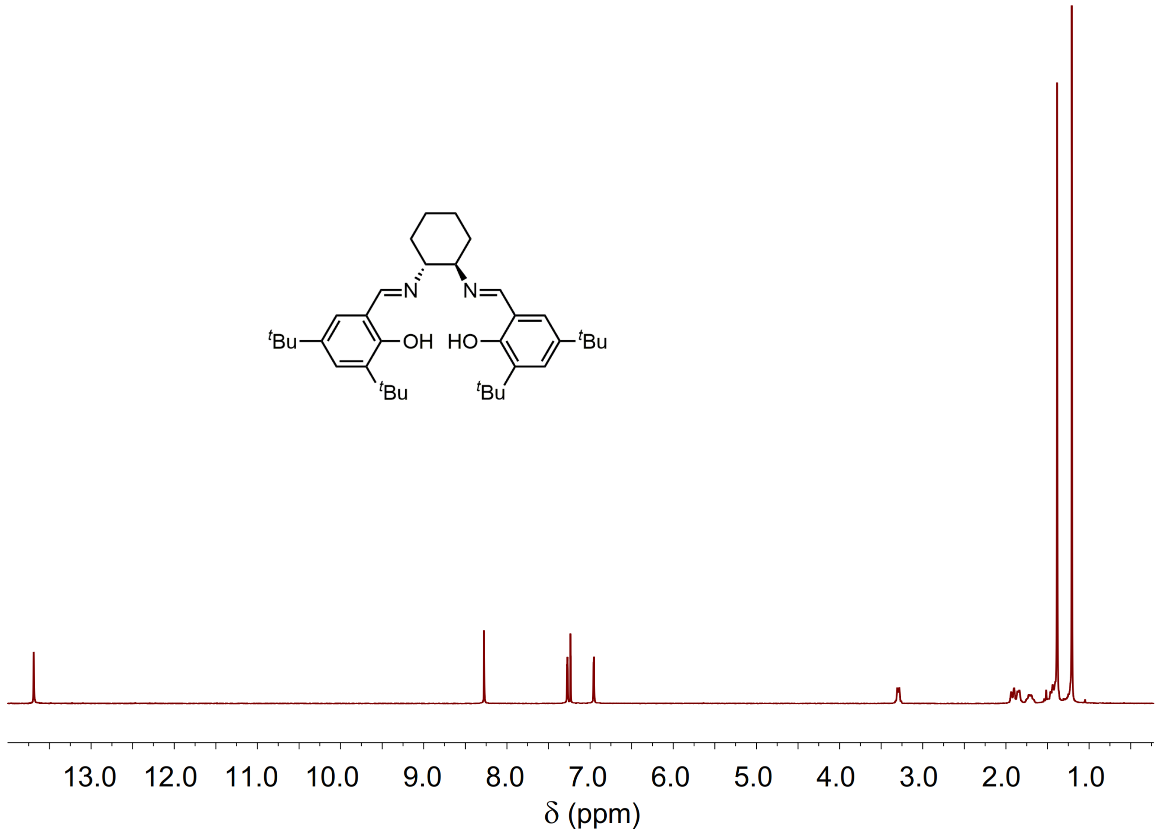

Supplementary Figure 8. ^{1}H NMR spectrum ($CDCl_3$) of ligand $\mathbf{L^{3a}}$.

(2) Salcy ligand $\mathbf{L^{3b}}$. $\mathbf{L^{3b}}$ was synthesized according to the general procedure detailed for $\mathbf{L^{3a}}$ expect that 3-*tert*-butyl-5-fluorosalicylaldehyde $\mathbf{L^{2b}}$ was used instead of 3,5-bis(*tert*-butyl)salicylaldehyde $\mathbf{L^{2a}}$. Yield: 75%. ^{1}H NMR (400 MHz, $CDCl_3$): δ 13.57 (s, 2H, OH), 8.20 (s, 2H, N=C*H*), 6.99 (dd, J = 10.7, 2.9 Hz, 2H, Ar-H), 6.67 (dd, J = 7.8, 3.0 Hz, 2H, Ar-H), 3.39 – 3.24 (m, 2H, NC*H*), 1.99 (m, 2H, Cy-*H*), 1.90 (m, 2H, Cy-*H*), 1.75 (m, 2H, Cy-*H*), 1.48 (m, 2H, Cy-*H*), 1.39 (s, 18H , ^{t}Bu).

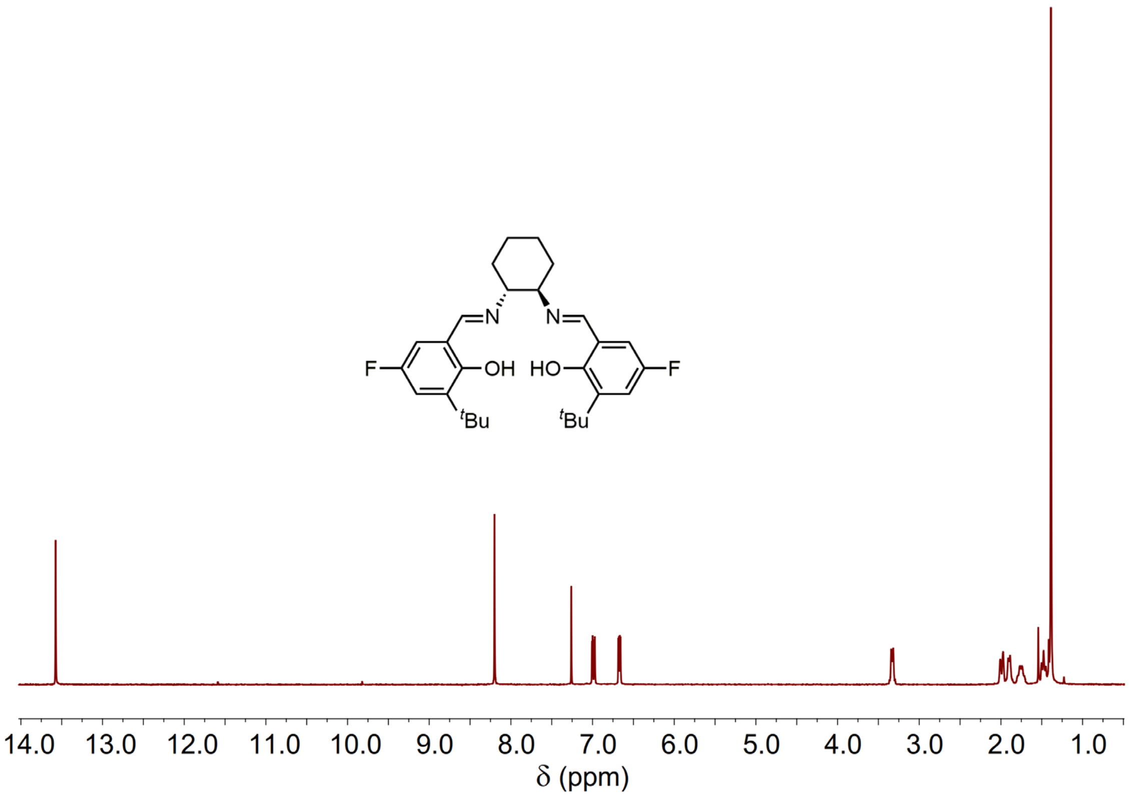

Supplementary Figure 9. ^{1}H NMR spectrum ($CDCl_3$) of ligand **L^{3b}**.

(3) Salcy ligand **L^{3c}**. **L^{3c}** was synthesized according to the general procedure detailed for **L^{3a}** expect that 3,5-dicumylsalicylaldehyde **L^{2c}** was used instead of 3,5-bis(*tert*-butyl)salicylaldehyde **L^{2a}**. Yield: 95%. ^{1}H NMR (400 MHz, $CDCl_3$): δ 13.26 (s, 2H, OH), 8.10 (s, 2H, N=C*H*), 7.32 – 7.08 (m, 22H, Ar-H), 6.93 (s, 2H, Ar-H), 3.15 (m, 2H, NC*H*), 1.75 (m, 4H, Cy-H), 1.67 (d, J = 2.6 Hz, 12H, -Me), 1.65 (s, 6H, Me), 1.58 (s, 6H, Me), 1.57 – 1.48 (m, 2H, Cy-H), 1.38 – 1.25 (m, 2H, Cy-H).

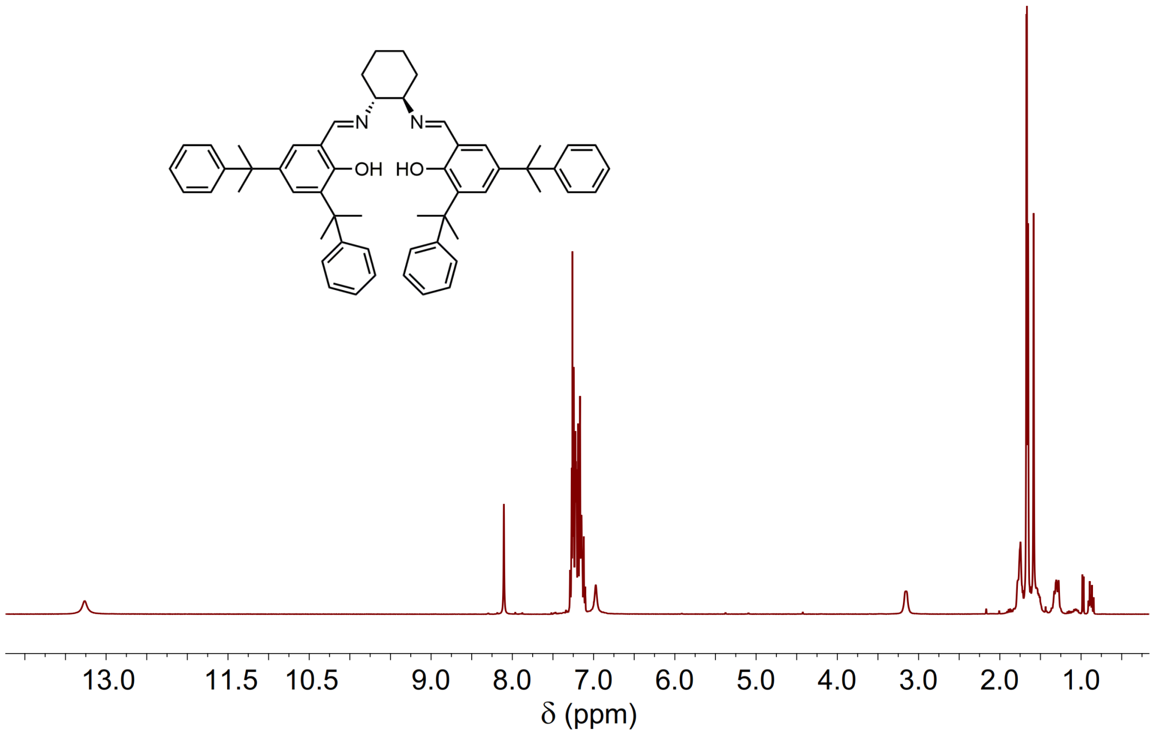

Supplementary Figure 10. ^{1}H NMR spectrum ($CDCl_3$) of ligand $\mathbf{L}^{3c}$.

(4) Salcy ligand $\mathbf{L}^{3d}$. According to the literature procedure,[10] a mixture of racemic *trans*-1,2-diaminocyclohexane (0.34 g, 3 mmol) and 3-trityl-5-methylsalicylaldehyde $\mathbf{L}^{2d}$ (2.28 g, 6 mmol) in 30 mL of dichloromethane was stirred under reflux overnight. All volatiles were removed via rotary evaporator. The product ($\mathbf{L}^{3d}$) was then was purified by recrystallization from ethanol. Yield: 2.38 g (95%). ^{1}H NMR (400 MHz, $CDCl_3$): δ 13.20 (s, 2H, OH), 7.96 (s, 2H, N=C*H*), 7.21–7.12(m, 30H, Ar-H), 7.04 (s, 2H, Ar-H), 6.95 (m, 2H, Ar-H), 3.10 (m, 2H, NC*H*), 2.24 (s, 6H, Me), 1.73 (m, 4H, Cy-H), 1.55 (m, 2H, Cy-H), 1.27 (m, 2H, Cy-H).

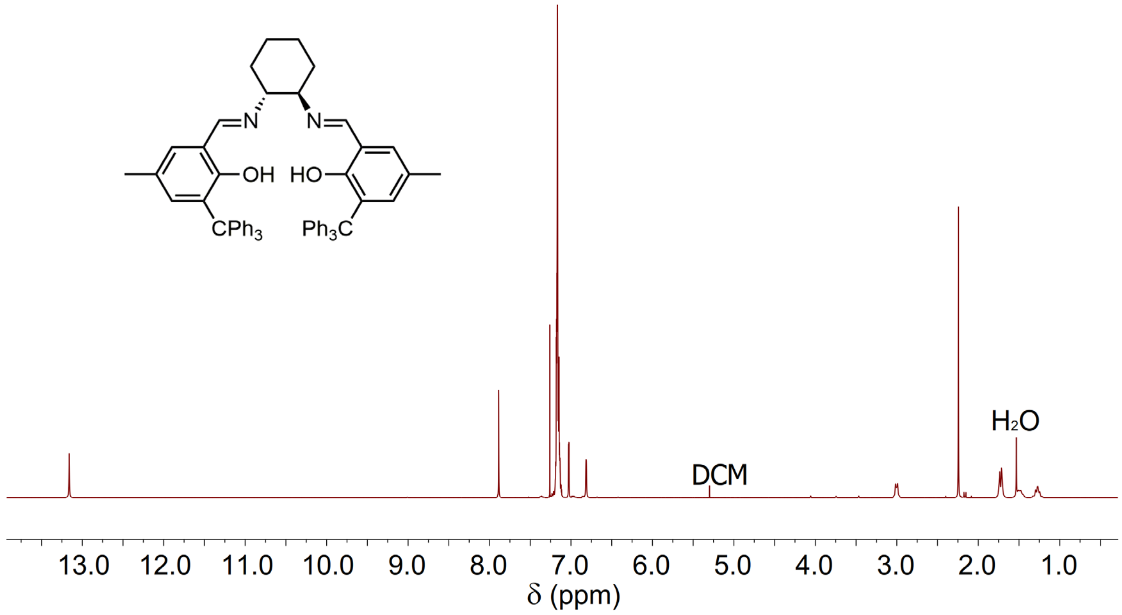

Supplementary Figure 11. ^{1}H NMR spectrum ($CDCl_3$) of ligand $\mathbf{L^{3d}}$.

(5) Salph ligand $\mathbf{L^{3e}}$. $\mathbf{L^{3e}}$ was synthesized according to the general procedure detailed for $\mathbf{L^{3a}}$ expect that 1,2-diaminobenzene was used instead of racemic *trans*-1,2-diaminocyclohexane.[11] Yield: 85%. ^{1}H NMR (400 MHz, $CDCl_3$): δ 13.54 (s, 2H, OH), 8.66 (s, 2H, N=C*H*), 7.44 (d, *J* = 2.4 Hz, 2H, Ar-H), 7.34 – 7.29 (m, 2H, Ar-H), 7.25 – 7.20 (m, 4H, Ar-H), 1.43 (s, 18H), 1.32 (s, 18H).

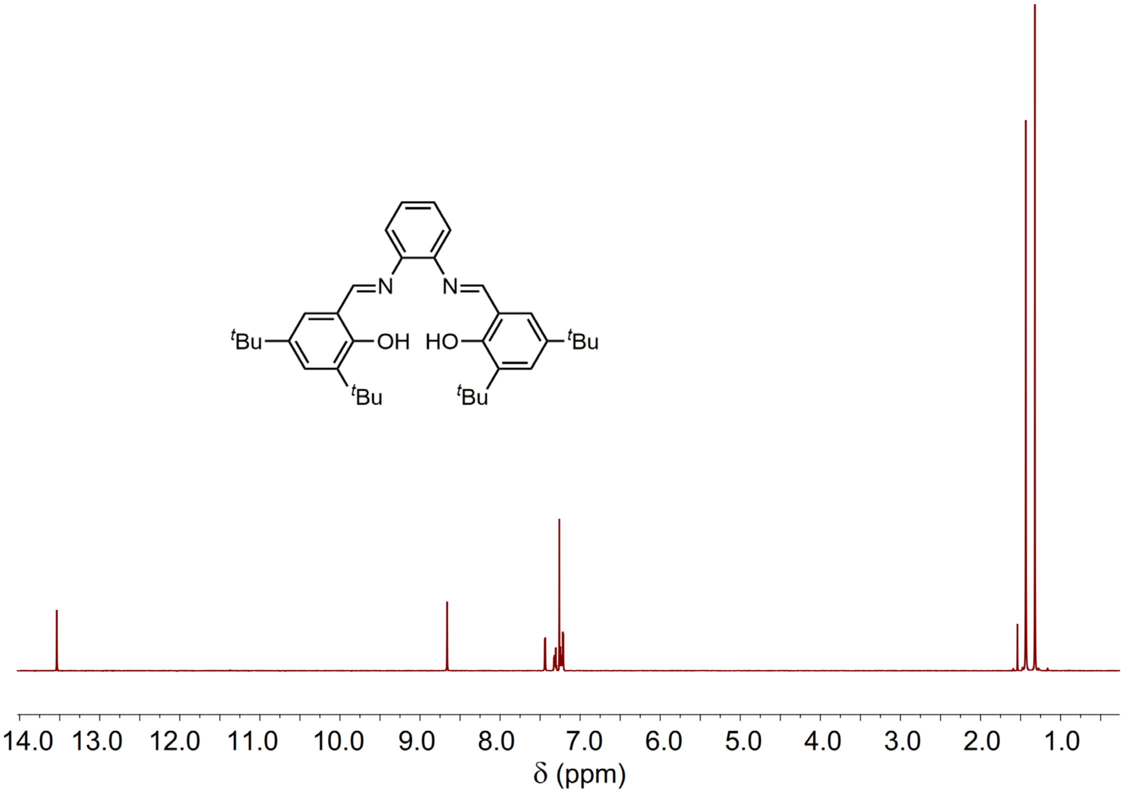

Supplementary Figure 12. ^{1}H NMR spectrum ($CDCl_3$) of ligand $\mathbf{L^{3e}}$.

Synthesis of yttrium complexes *rac*-4a-d and 4e

(1) Yttrium complex **4a**. Synthesis of yttrium complex **4a** followed the literature procedure[12-13] with minor modifications detailed below. A solution of salcy ligand $\mathbf{L^{3a}}$ (0.547 g, 1.00 mmol) in hexanes (20 mL) was added to a solution of $Y[N(SiHMe_2)_2]_3(THF)_2$ (0.630 g, 1.00 mmol) in hexanes (20 mL) and stirred for 24 h at room temperature. The volatiles were removed in vacuo, and the residue was washed with cold hexanes. The product was obtained as pale yellow solid. Yield: 0.60 g (72%). ^{1}H NMR (400 MHz, C_6D_6): δ 8.03 (s, 1H, Ar-H, N=C*H*), 7.90 (s, 1H, N=C*H*), 7.73 (dd, J = 8.9, 2.6 Hz, 2H, Ar-H), 7.32 (d, J = 2.6 Hz, 1H, Ar-H), 7.10 (d, J = 2.5 Hz, 1H, Ar-H), 5.07 (dt, J = 5.9, 2.8 Hz, 2H, Si*H*-), 4.79 (m, 1H, NC*H*), 3.99 (m, 4H, THF), 2.29 (m, 1H, , NC*H*), 1.76 (s, 9H, ^{t}Bu), 1.61 (s, 9H, ^{t}Bu), 1.65 – 1.32 (m, 6H, Cy-H), 1.46 (m, 4H, THF), 1.40 (s, 9H, ^{t}Bu), 1.37 (s, 9H, ^{t}Bu), 1.04 – 0.82 (m, 2H, Cy-H), 0.33 (dd, J = 4.2, 3.2 Hz, 12H, SiMe). ^{13}C NMR (100 MHz, C_6D_6): δ 171.0, 164.8, 164.0, 162.8, 139.5, 139.1, 136.8,

136.7, 130.3, 129.8, 129.7, 129.6, 122.9, 122.6, 72.6, 70.3, 65.6, 35.9, 35.8, 34.2, 33.3, 31.9, 31.8, 30.5, 30.2, 27.5, 25.7, 25.4, 25.1, 3.4, 3.1.

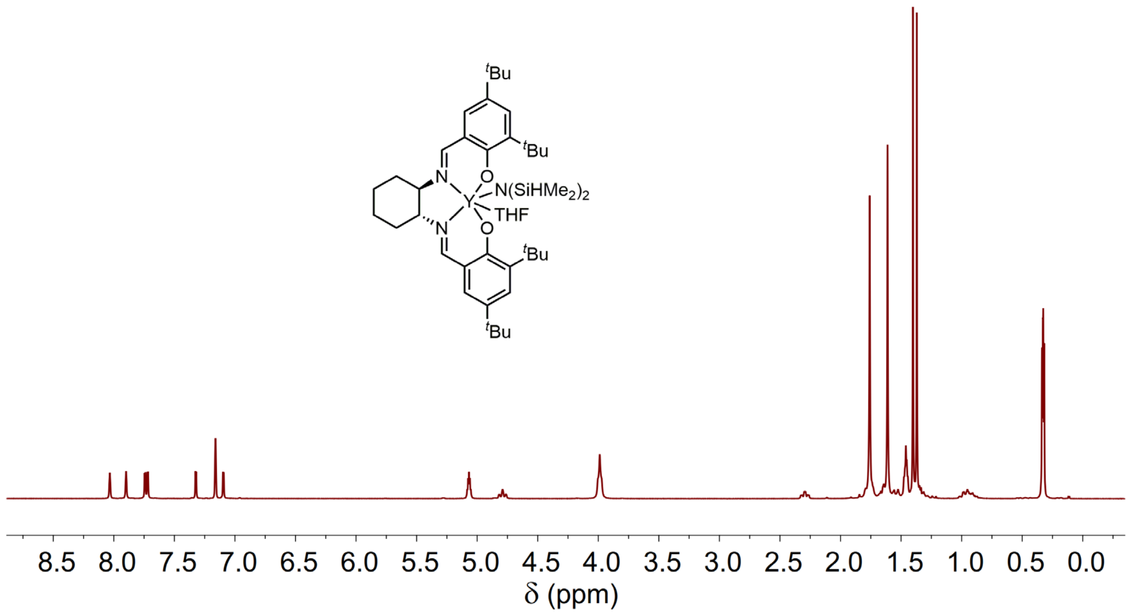

Supplementary Figure 13. ^{1}H NMR spectrum (C_6D_6) of yttrium complex **4a**.

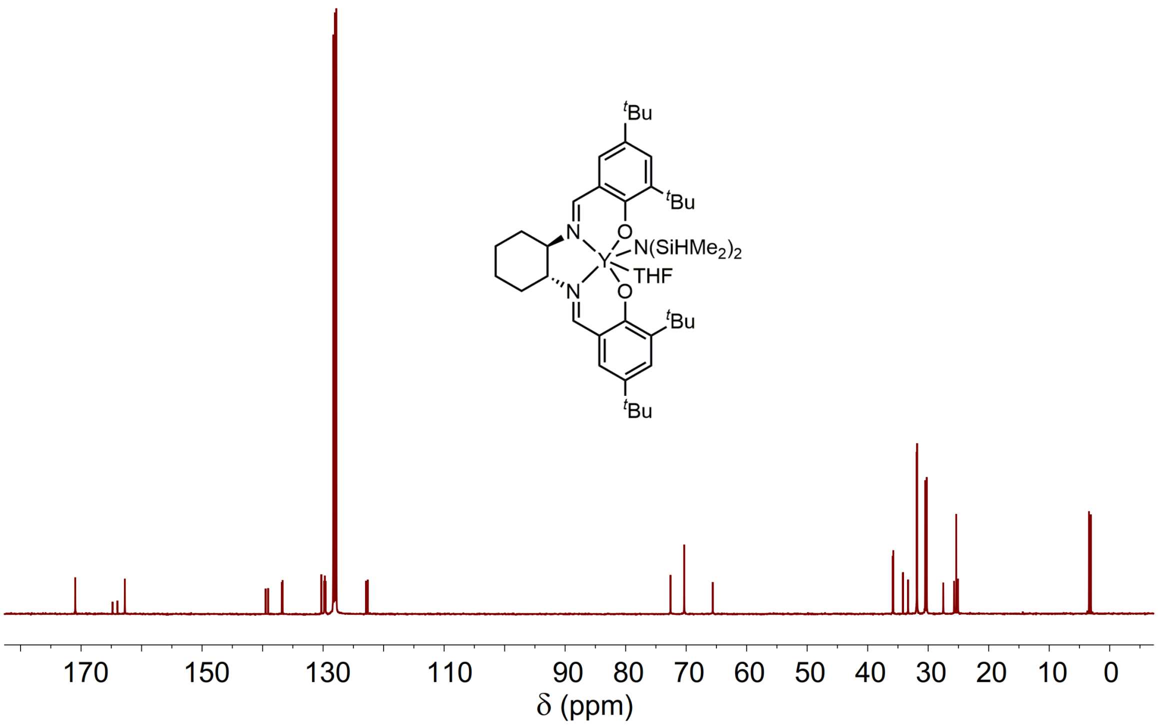

Supplementary Figure 14. ^{13}C NMR spectrum (C_6D_6) of yttrium complex **4a**.

(2) Yttrium complex **4b**. Yttrium complex **4b** was synthesized according to the general procedure detailed for **4a** expect that salcy ligand $\mathbf{L}^{\mathbf{3b}}$ was used instead of $\mathbf{L}^{\mathbf{3a}}$. The product was obtained as pale yellow solid. Yield: 73%. ^{1}H NMR (400 MHz, C_6D_6): δ 7.66 (s, 1H, N=C*H*), 7.50 (s, 1H, N=C*H*), 7.35 (ddd, J = 10.5, 3.3, 1.8 Hz, 2H, Ar-H), 6.81 (dd, J = 8.2, 3.3 Hz, 1H, Ar-H), 6.64 (dd, J = 8.4, 3.3 Hz, 1H, Ar-H), 5.01 (dt, J = 6.0, 3.0 Hz, 2H, Si*H*-), 4.47 (m, 1H, NC*H*), 3.90 (m, 4H, THF), 2.18 – 2.02 (m, 1H, NC*H*), 1.68 – 1.39 (m, 4H, Cy-H), 1.56 (s, 9H, ^{t}Bu), 1.48 (s, 9H, ^{t}Bu), 1.44 (m, 4H, THF), 1.31 – 1.14 (m, 2H, Cy-H), 0.91 – 0.80 (m, 2H, Cy-H), 0.29 (dd, J = 3.0, 1.0 Hz, 12H, SiMe). ^{13}C NMR (100 MHz, C_6D_6): δ 169.6, 162.8, 162.0, 161.8, 154.7, 152.4, 142.0, 141.7, 122.2, 120.6, 120.4, 120.2, 117.3 (d, J = 21.6 Hz), 116.9 (d, J = 21.5 Hz), 71.9, 70.5, 65.8, 35.7, 35.6, 33.1, 29.8, 29.6, 27.4, 25.5, 25.3, 24.9, 3.3, 3.0.

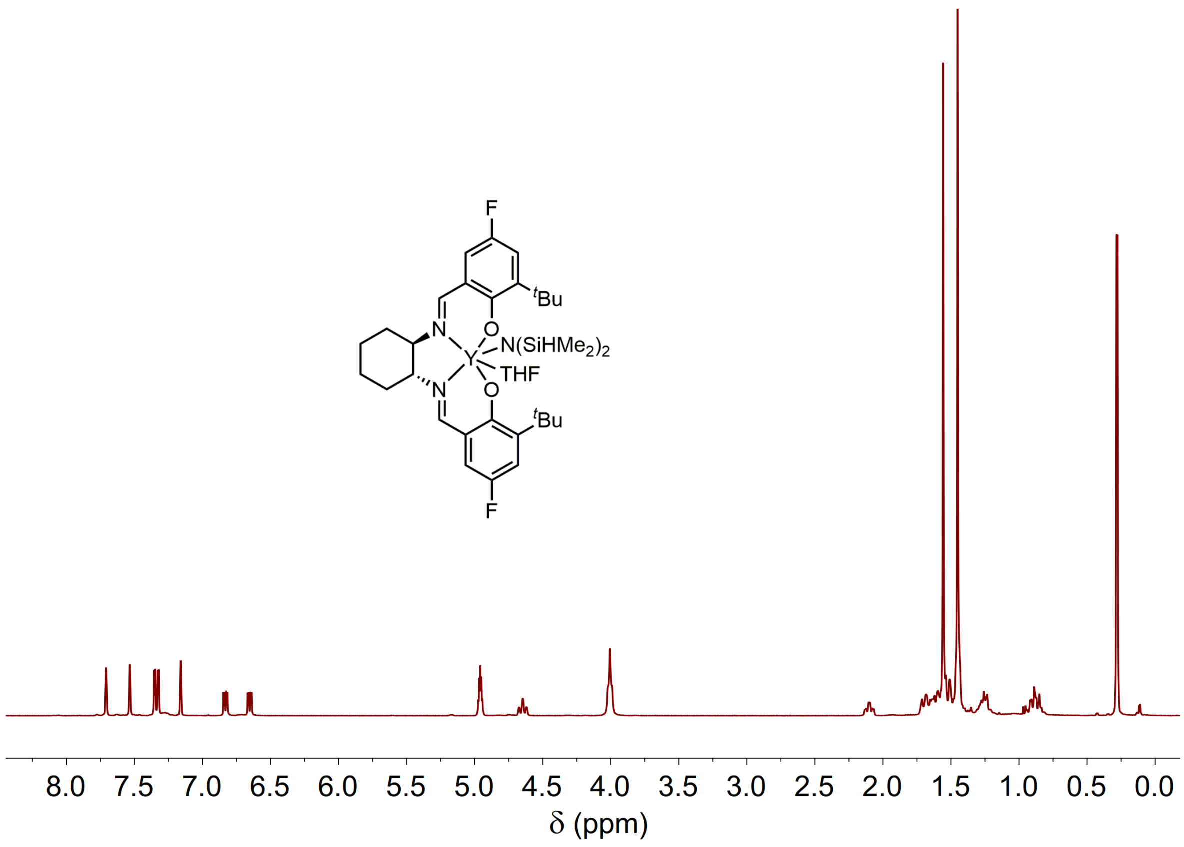

Supplementary Figure 15. ^{1}H NMR spectrum (C_6D_6) of yttrium complex **4b**.

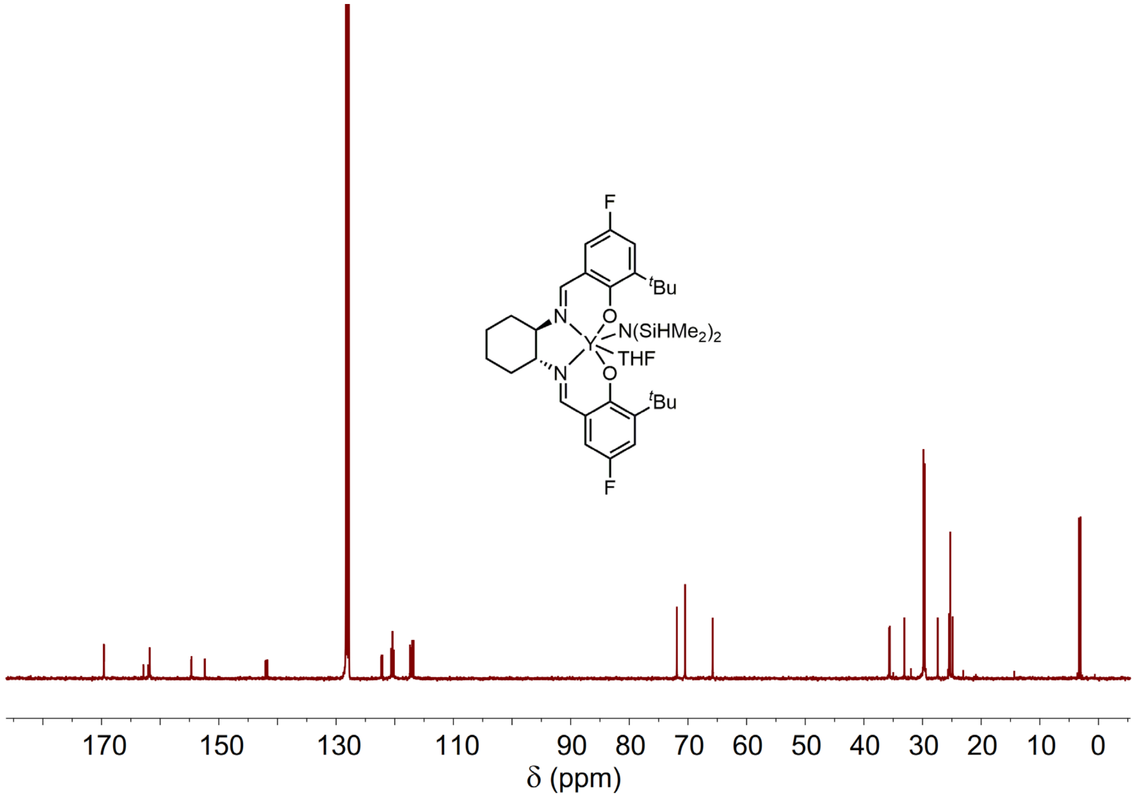

Supplementary Figure 16. ^{13}C NMR spectrum (C_6D_6) of yttrium complex **4b**.

(3) Yttrium complex **4c**. Yttrium complex **4c** was synthesized according to the general procedure detailed for **4a** expect that salcy ligand $\mathbf{L^{3c}}$ was used instead of $\mathbf{L^{3a}}$. The product was obtained as pale yellow solid. Yield: 83%. ^1H NMR (400 MHz, C_6D_6): δ 7.82 (s, 1H, N=C*H*), 7.70 (s, 1H, N=C*H*), 7.66 (m, 2H, Ar-H), 7.50 – 7.40 (m, 4H, Ar-H), 7.40 – 7.34 (m, 4H, Ar-H), 7.28 – 7.18 (m, 9H, Ar-H), 7.15 – 7.02 (m, 5H, Ar-H), 4.61 (m, 2H, Si*H*-), 4.38 (m, 1H, NC*H*), 3.36 (s, 4H, THF), 2.12 (m, 1H, NC*H*), 2.10 (s, 3H, Me), 1.95 (s, 3H, Me), 1.80 (s, 3H, Me), 1.72 (d, J = 2.4 Hz, 6H, Me), 1.70 (s, 6H, Me), 1.66 (s, 3H, Me), 1.59 – 1.19 (m, 10H, Cy-H, THF), 0.84 – 0.57 (m, 2H, Cy-H), 0.10 (m, 12H, SiMe). ^{13}C NMR (100 MHz, C_6D_6): δ 170.5, 164.6, 164.0, 162.6, 152.1, 151.6, 151.5, 151.4, 138.5, 138.0, 136.6, 136.4, 133.0, 132.9, 132.7, 132.2, 128.4, 128.3, 128.2, 127.2, 126.7, 126.6, 126.0, 125.5, 125.3, 123.2, 122.8, 72.1, 69.4, 65.4, 43.4, 43.4, 42.6, 33.6, 33.2, 33.0, 31.4, 31.3, 28.6, 27.7, 27.3, 25.9, 25.6, 24.9, 3.2, 2.9.

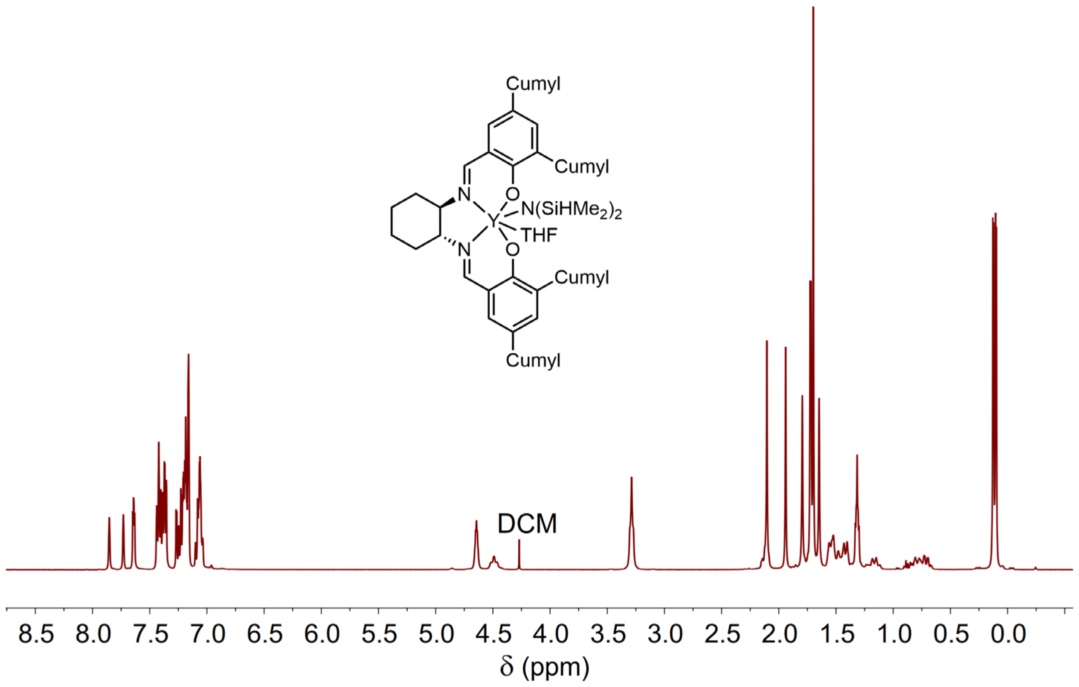

Supplementary Figure 17. ^{1}H NMR spectrum (C_6D_6) of yttrium complex **4c**.

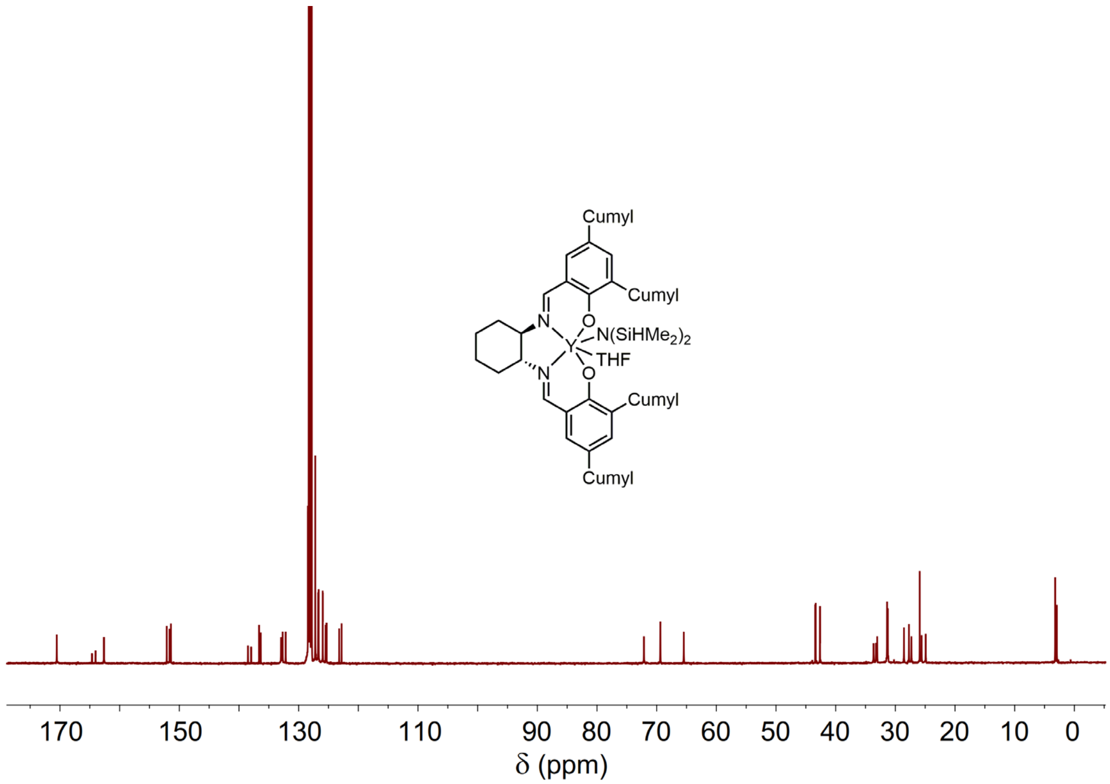

Supplementary Figure 18. ^{13}C NMR spectrum (C_6D_6) of yttrium complex **4c**.

(4) Yttrium complex **4d**. A solution of salen ligand $\mathbf{L^{3d}}$ (0.585 g, 0.70 mmol) in toluene (15 mL) was added to a solution of $Y[N(SiHMe_2)_2]_3(THF)_2$ (0.441 g, 0.70 mmol) in toluene (15 mL) and stirred for 3 days at room temperature. The volatiles were removed in vacuo, and the residue was washed with cold hexanes. The product was obtained as yellow solid. Yield: 0.602 g (82%). Anal. Calc. for $C_{64}H_{66}N_3O_2Si_2Y$: C, 72.9; H, 6.3; N, 4.0. Found: C, 72.9; H, 6.6; N, 3.7%.

^1H NMR (400 MHz, C_6D_6): δ 7.83 (s, 1H, N=C*H*), 7.68 (s, 1H, N=C*H*), 7.58 (dd, J = 5.2, 2.2 Hz, 2H, Ar-H), 7.50 (m, 6H, Ar-H), 7.42 (m, 6H, Ar-H), 7.13 – 6.96 (m, 19H, Ar-H), 6.82 (d, J = 1.9 Hz, 1H, Ar-H), 4.39 (dt, J = 5.9, 2.9 Hz, 2H, Si*H*-), 3.97 (m, 1H, NC*H*), 2.14 – 2.10 (m, 1H, NC*H*), 2.10 (s, 3H, Me), 2.06 (s, 3H, Me), 1.90 – 1.71 (m, 2H, Cy-H), 1.61 – 1.42 (m, 2H, Cy-H), 1.23 – 0.99 (m, 2H, Cy-H), 0.91 – 0.67 (m, 2H, Cy-H), 0.15 (d, J = 3.0 Hz, 6H, SiMe), 0.01 (d, J = 2.9 Hz, 6H, SiMe). ^{13}C NMR (100 MHz, C_6D_6): δ 168.7, 164.0, 163.3, 163.1, 147.2, 139.5, 138.1, 136.7, 135.3, 131.9, 131.8, 127.7, 127.6, 126.0, 125.7, 124.4, 124.2, 123.5, 123.4, 69.4, 64.9, 64.5, 64.4, 31.6, 28.2, 25.2, 24.6, 20.7, 3.4, 3.0.

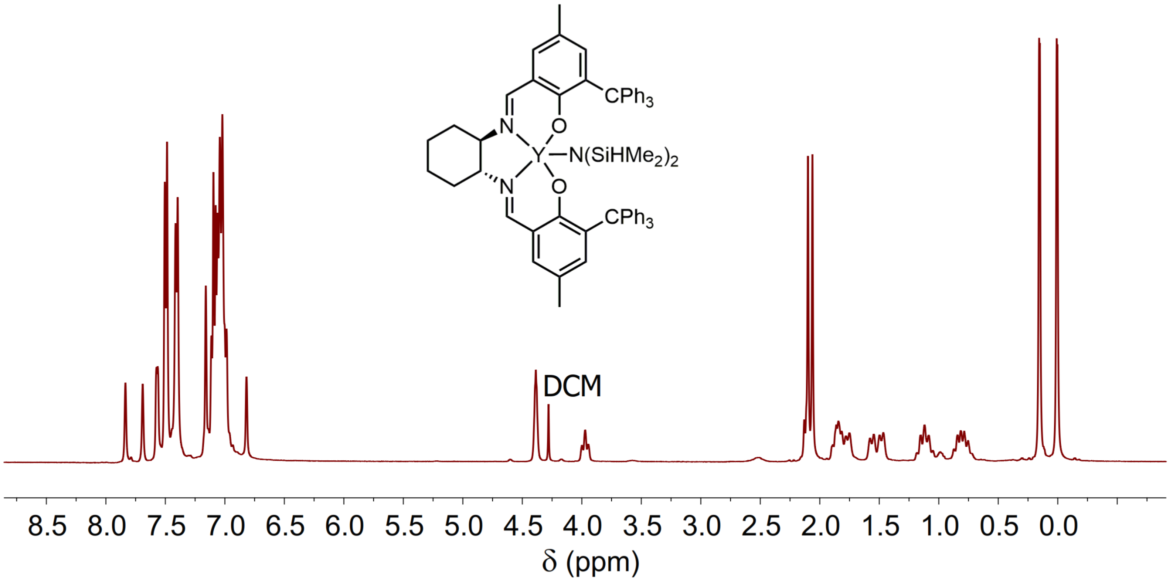

Supplementary Figure 19. ^1H NMR spectrum (C_6D_6) of yttrium complex **4d**.

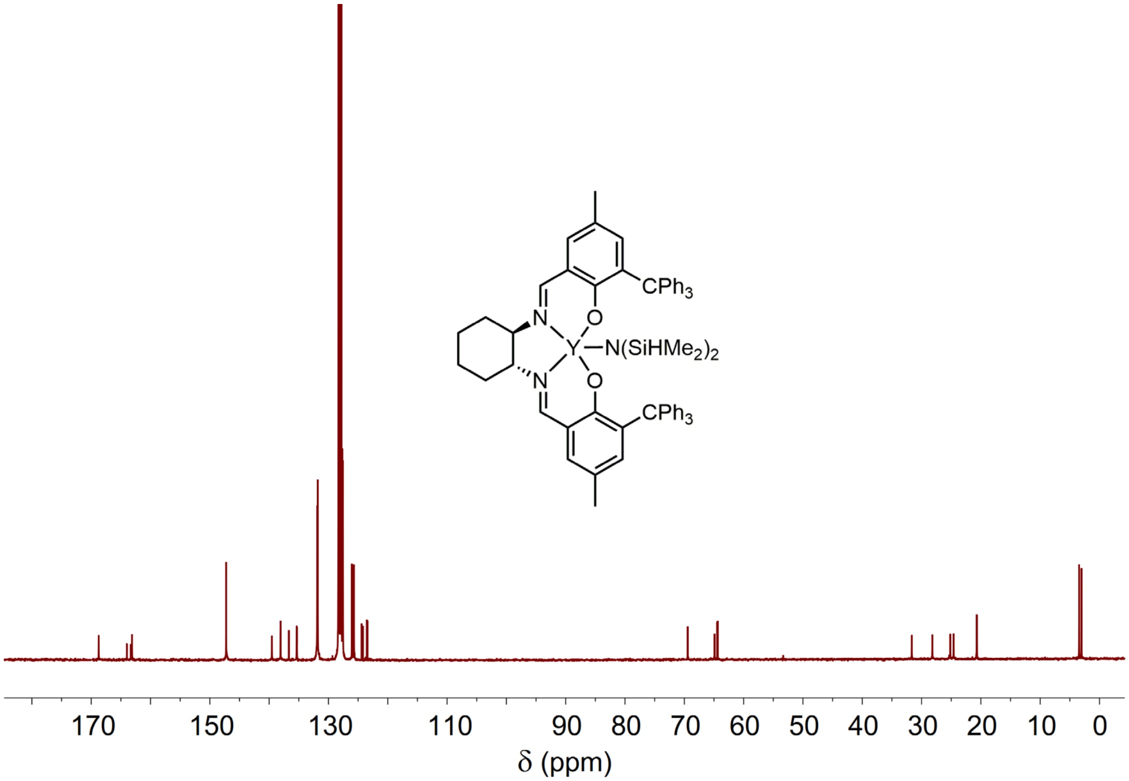

Supplementary Figure 20. ^{13}C NMR spectrum (C_6D_6) of yttrium complex **4d**.

(5) Yttrium complex **4e**. Yttrium complex **4e** was synthesized according to the general procedure detailed for **4a** expect that salph ligand $\mathbf{L^{3e}}$ was used instead of $\mathbf{L^{3a}}$. The product was obtained as yellow solid. Yield: 58%. ^{1}H NMR (400 MHz, C_6D_6): δ 8.25 (s, 2H, N=C*H*), 7.77 (d, J = 2.5 Hz, 2H, Ar-H), 7.21 (d, J = 2.5 Hz, 2H, Ar-H), 7.08 – 6.96 (m, 4H, Ar-H), 4.81 (dt, J = 5.7, 2.8 Hz, 2H, Si*H*-), 4.17 (m, 4H, THF), 1.69 (s, 18H, ^{t}Bu), 1.55 – 1.45 (m, 4H, THF), 1.39 (s, 18H, ^{t}Bu), 0.07 (d, J = 3.0 Hz, 12H, $SiMe_3$). ^{13}C NMR (100 MHz, C_6D_6): δ 166.5, 165.8, 146.2, 139.8, 137.3, 130.8, 130.6, 127.5, 122.7, 118.8, 70.8, 35.9, 34.2, 31.8, 30.4, 25.4, 3.1.

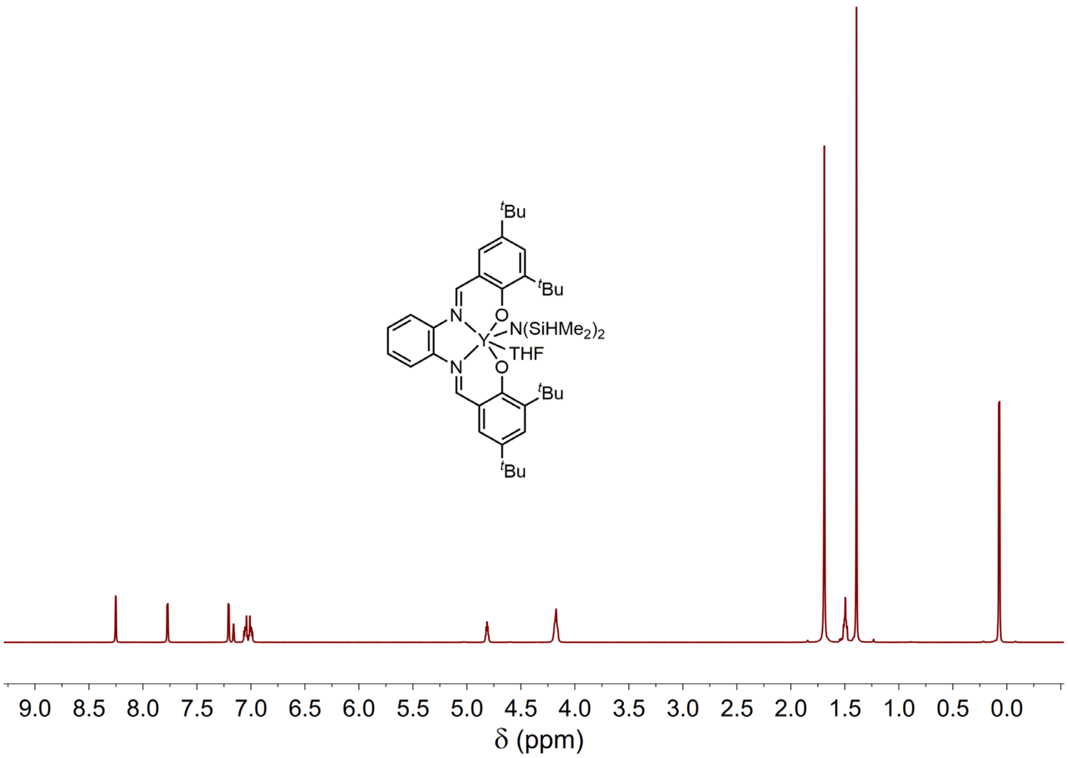

Supplementary Figure 21. ^{1}H NMR spectrum (C_6D_6) of yttrium complex **4e**.

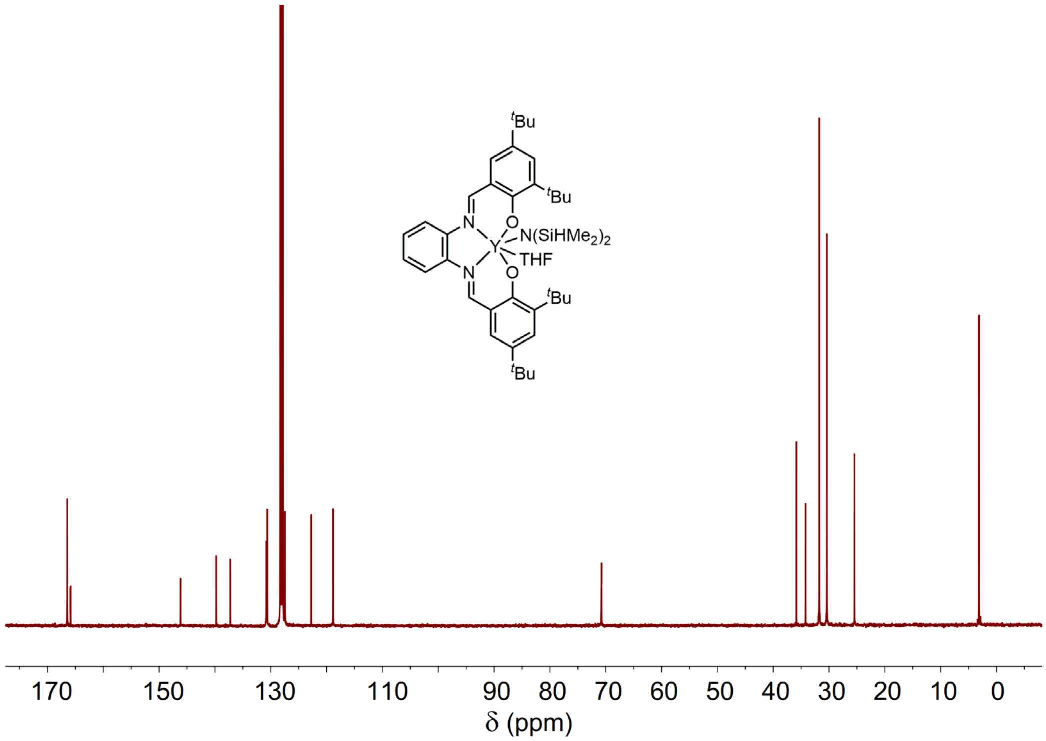

Supplementary Figure 22. ^{13}C NMR spectrum (C_6D_6) of yttrium complex **4e**.

Synthesis of Enantiomeric Yttrium Complexes (*R,R*)-4d and (*S,S*)-4d

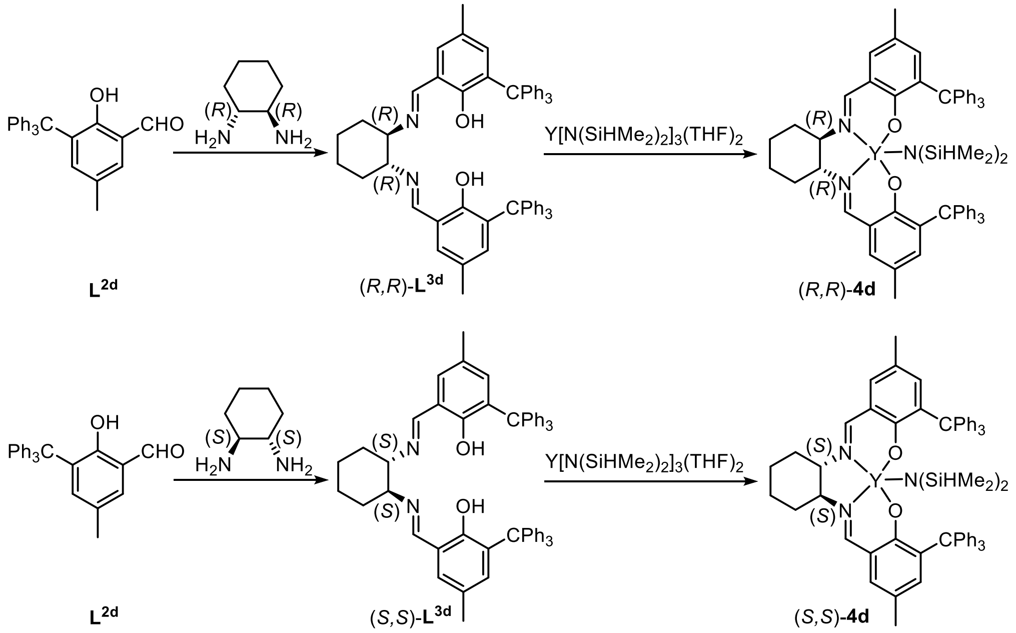

Supplementary Figure 23. Synthesis of Enantiomeric Yttrium Complexes (*R,R*)-**4d** and (*S,S*)-**4d**

Synthesis of enantiopure salcy ligand $\mathbf{L^{3d}}$

(1). Salcy ligand (*R,R*)-$\mathbf{L^{3d}}$. A mixture of (*1R,2R*)-(-)-1,2-diaminocyclohexane (0.34 g, 3 mmol) and 3-trityl-5-methylsalicylaldehyde $\mathbf{L^{2d}}$ (2.28 g, 6 mmol) in 30 mL of dichloromethane was stirred under reflux overnight. All volatiles were removed via rotary evaporator. The product [(*R,R*)-$\mathbf{L^{3d}}$] was then purified by recrystallization from ethanol; yield: 2.33 g (93%). ^{1}H NMR (400 MHz, $CDCl_3$): δ 13.14 (s, 2H, OH), 7.88 (s, 2H, N=C*H*), 7.22 – 7.08 (m, 30H, Ar-H), 7.03 (d, J = 2.0 Hz, 2H, Ar-H), 6.81 (d, J = 1.7 Hz, 2H, Ar-H), 3.08 – 2.89 (m 2H, NC*H*), 2.24 (s, 6H, Me), 1.80 – 1.66 (m, 4H, Cy-H), 1.57 – 1.41 (m, 2H, Cy-H), 1.35 – 1.17 (m, 2H, Cy-H). $[\alpha]_D^{23}$ = –334.4° (c = 0.519 g/100 mL, chloroform).

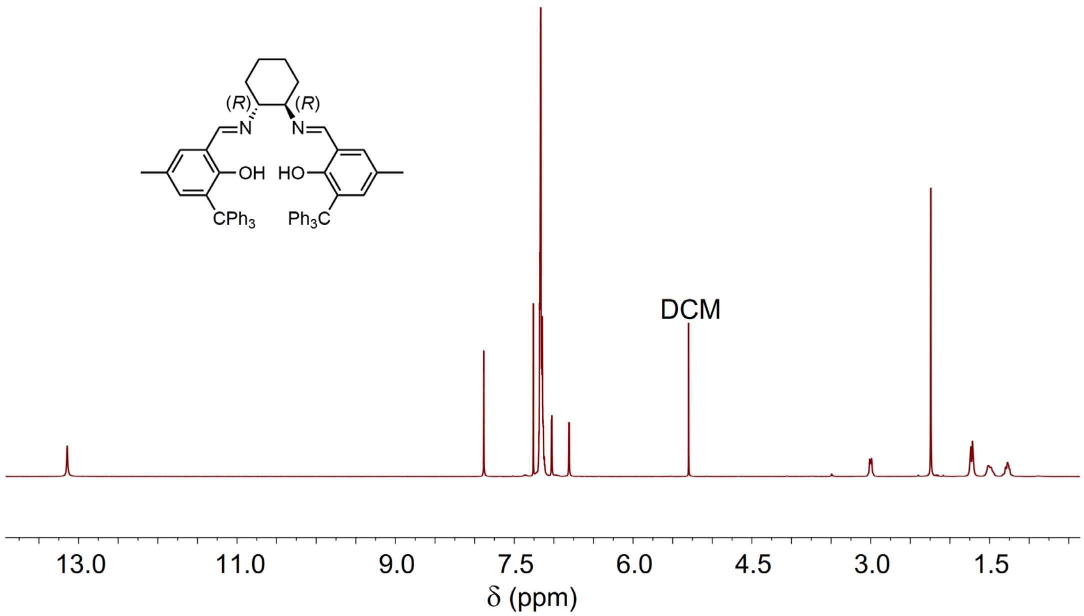

Supplementary Figure 24. ^{1}H NMR spectrum of salcy ligand (*R,R*)-**L^{3d}** in $CDCl_3$

(2). Salcy ligand (*S,S*)-**L^{3d}**. A mixture of (*1S,2S*)-(+)-1,2-diaminocyclohexane (0.34 g, 3 mmol) and 3-trityl-5-methylsalicylaldehyde **L^{2d}** (2.28 g, 6 mmol) in 30 mL of dichloromethane was stirred under reflux overnight. All volatiles were removed via rotary evaporator. The product [(*S,S*)-**L^{3d}**] was then purified by recrystallization from ethanol; yield: 2.31 g (92%). ^{1}H NMR (400 MHz, $CDCl_3$): *δ* 13.14 (s, 2H, OH), 7.88 (s, 2H, N=C*H*), 7.21 – 7.09 (m, 30H, Ar-H), 7.03 (d, *J* = 1.9 Hz, 2H, Ar-H), 6.81 (d, *J* = 1.7 Hz, 2H, Ar-H), 3.07 – 2.91 (m 2H, NC*H*), 2.24 (s, 6H, Me), 1.81 – 1.66 (m, 4H, Cy-H), 1.59 – 1.40 (m, 2H, Cy-H), 1.37 – 1.19 (m, 2H, Cy-H). $[\alpha]_D^{23}$ = +336.8 (*c* = 0.366 g/100 mL, chloroform).

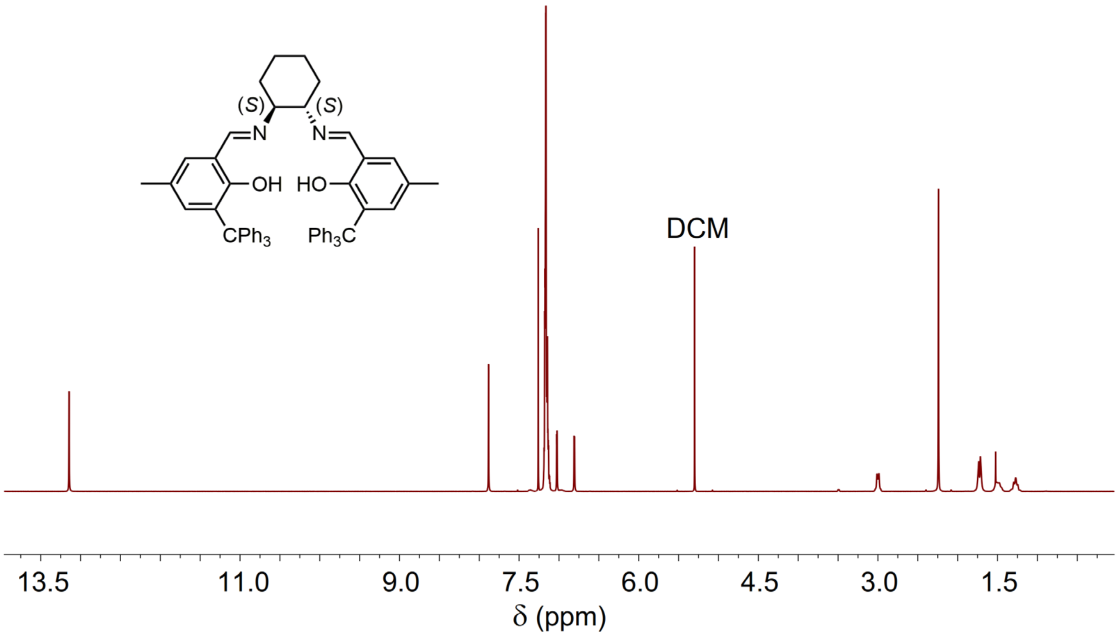

Supplementary Figure 25. ^{1}H NMR spectrum of salcy ligand (*S,S*)-**L^{3d}** in $CDCl_3$.

Synthesis of enantiomeric yttrium complexes (*R,R*)-4d and (*S,S*)-4d

(1). Yttrium complex (*R,R*)-**4d**. A solution of salcy ligand (*R,R*)-**L^{3d}** (0.668 g, 0.80 mmol) in toluene (15 mL) was added to a solution of $Y[N(SiHMe_2)_2]_3(THF)_2$ (0.504 g, 0.80 mmol) in toluene (15 mL) and stirred for 3 days at room temperature. The volatiles were removed in vacuo, and the residue was washed with cold hexanes. The product was obtained as yellow solid; yield: 0.680 g (81%). ^{1}H NMR (400 MHz, C_6D_6): δ 7.83 (s, 1H, N=C*H*), 7.68 (s, 1H, N=C*H*), 7.58 (dd, *J* = 5.3, 2.3 Hz, 2H, Ar-H), 7.53 – 7.46 (m, 6H, Ar-H), 7.45 – 7.37 (m, 6H, Ar-H), 7.13 – 6.95 (m, 19H, Ar-H), 6.82 (d, *J* = 2.1 Hz, 1H, Ar-H), 4.39 (dt, *J* = 5.9, 2.9 Hz, 2H, Si*H*-), 3.97 (m, 1H, NC*H*), 2.10 (s, 3H, Me), 2.06 (s, 3H, Me), 1.92 – 1.69 (m, 3H, Cy-H), 1.62 – 1.41 (m, 2H, Cy-H), 1.23 – 0.99 (m, 2H, Cy-H), 0.91 – 0.68 (m, 2H, Cy-H), 0.15 (d, *J* = 3.0 Hz, 6H, $SiMe_2$), 0.01 (d, *J* = 3.0 Hz, 6H, $SiMe_2$). ^{13}C NMR (101 MHz, C_6D_6): δ 168.7, 164.0 163.3, 163.1, 147.2, 139.5, 138.1, 136.7, 135.3, 131.9, 131.8, 127.7, 127.6, 126.0, 125.7, 124.4, 124.2, 123.5, 123.4, 69.4, 64.9, 64.5, 64.4, 31.6, 28.2, 25.2, 24.6, 20.7, 3.4, 3.0. $[\alpha]_D^{23}$ = –376.4° (*c* = 0.426 g/100 mL, toluene).

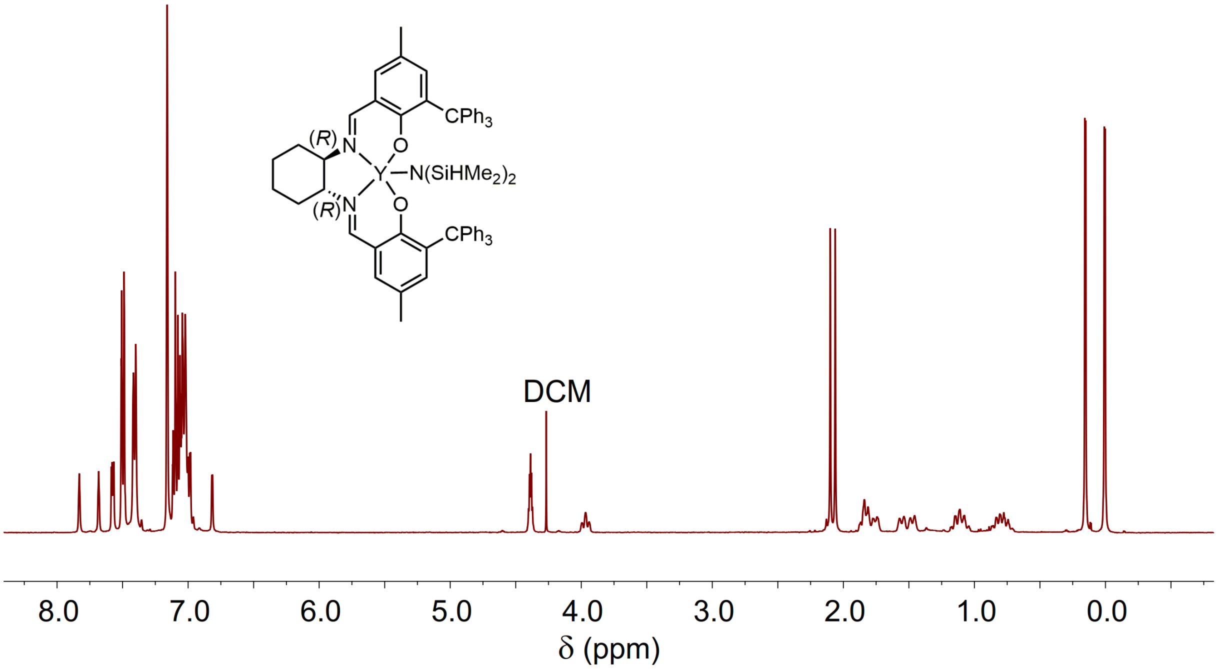

Supplementary Figure 26. ^{1}H NMR spectrum of yttrium complex (*R,R*)-**4d** in C_6D_6.

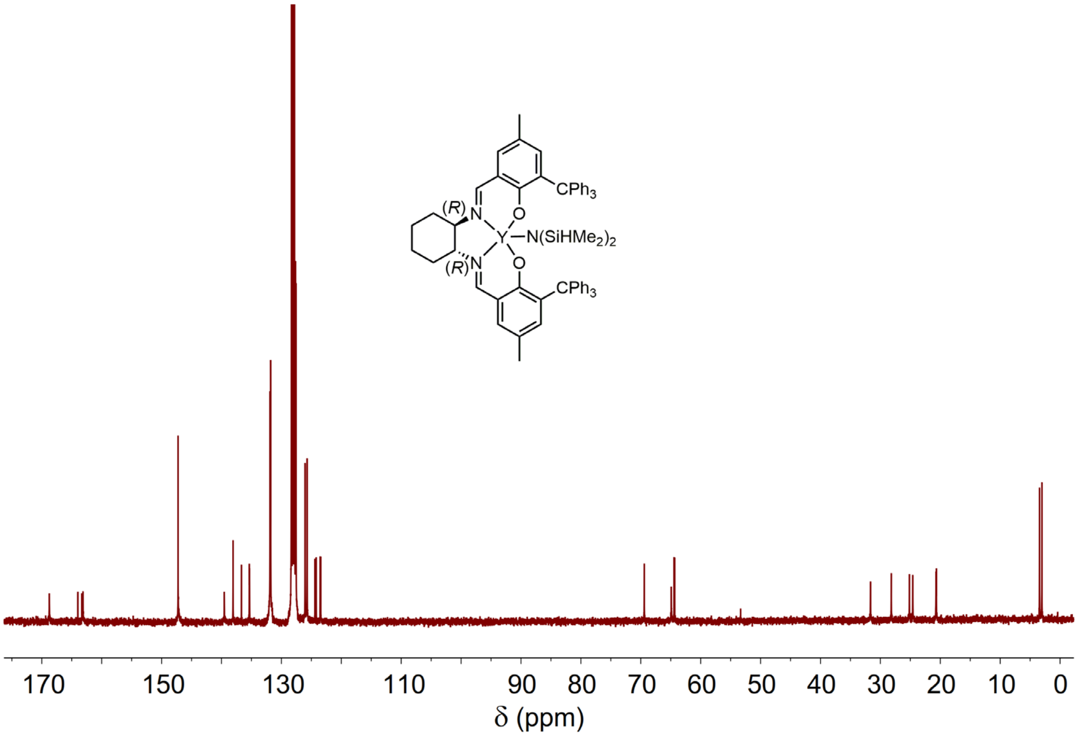

Supplementary Figure 27. ^{13}C NMR spectrum of yttrium complex (*R,R*)-**4d** in C_6D_6.

(2). Yttrium complex (*S,S*)-**4d**. A solution of salcy ligand (*S,S*)-**L**[3d] (0.668 g, 0.80 mmol) in toluene (15 mL) was added to a solution of $Y[N(SiHMe_2)_2]_3(THF)_2$ (0.504 g, 0.80 mmol) in toluene (15 mL) and stirred for 3 days at room temperature. The volatiles were removed in vacuo, and the residue was washed with cold hexanes. The product was obtained as yellow solid; yield: 0.710 g (84%). ^1H NMR (400 MHz, C_6D_6): δ 7.83 (s, 1H, N=C*H*), 7.68 (s, 1H, N=C*H*), 7.58 (dd, J = 5.3, 2.2 Hz, 2H, Ar-H), 7.53 – 7.45 (m, 6H, Ar-H), 7.45 – 7.38 (m, 6H, Ar-H), 7.14 – 6.95 (m, 19H, Ar-H), 6.82 (d, J = 1.9 Hz, 1H, Ar-H), 4.39 (dt, J = 5.8, 2.8 Hz, 2H, Si*H*-), 3.97 (t, J = 10.7 Hz, 1H, NC*H*), 2.10 (s, 3H, Me), 2.06 (s, 3H, Me), 1.93 – 1.71 (m, 3H, Cy-H), 1.63 – 1.42 (m, 2H, Cy-H), 1.29 – 1.01 (m, 2H, Cy-H), 0.93 – 0.71 (m, 2H, Cy-H), 0.15 (d, J = 3.0 Hz, 6H, $SiMe_2$), 0.01 (d, J = 2.9 Hz, 6H, $SiMe_2$). ^{13}C NMR (101 MHz, c_6d_6) δ 168.7, 164.0, 163.3, 163.1, 147.2, 139.5, 138.1, 136.7, 135.3, 131.9, 131.8, 127.7, 127.6, 126.0, 125.7, 124.4, 124.2, 123.6, 123.4, 69.4, 64.9, 64.5, 64.4, 31.6, 28.2, 25.2, 24.6, 20.7, 3.4, 3.0. $[\alpha]_D^{23}$ = +384.8 (c = 0.442 g/100 mL, toluene).

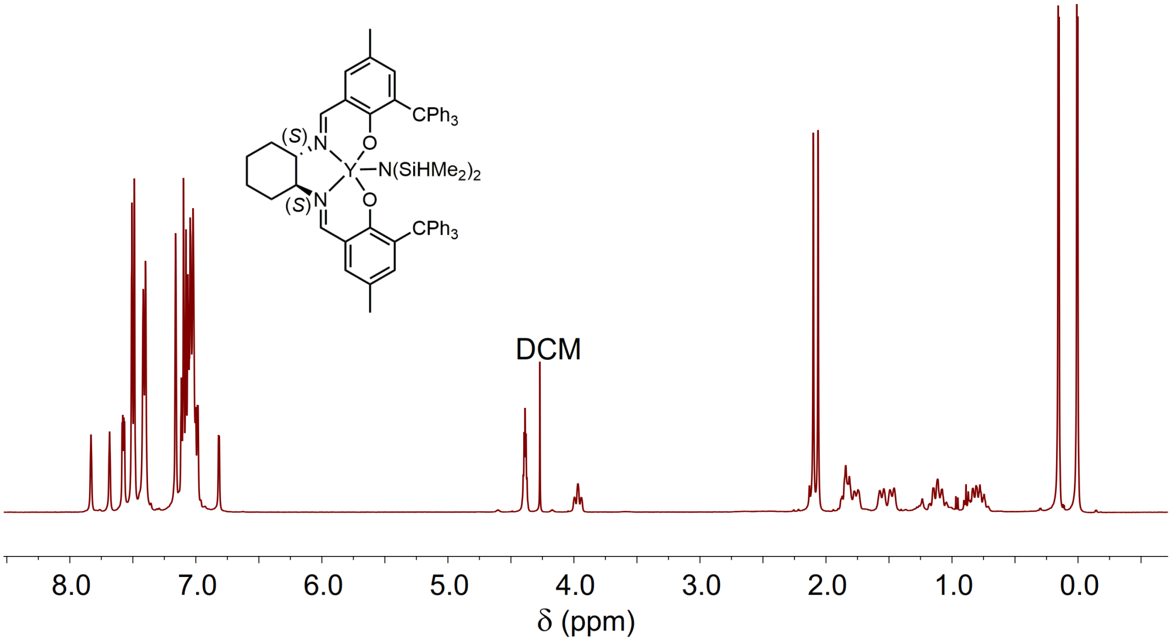

Supplementary Figure 28. ^1H NMR spectrum of yttrium complex (*S,S*)-**4d** in C_6D_6.

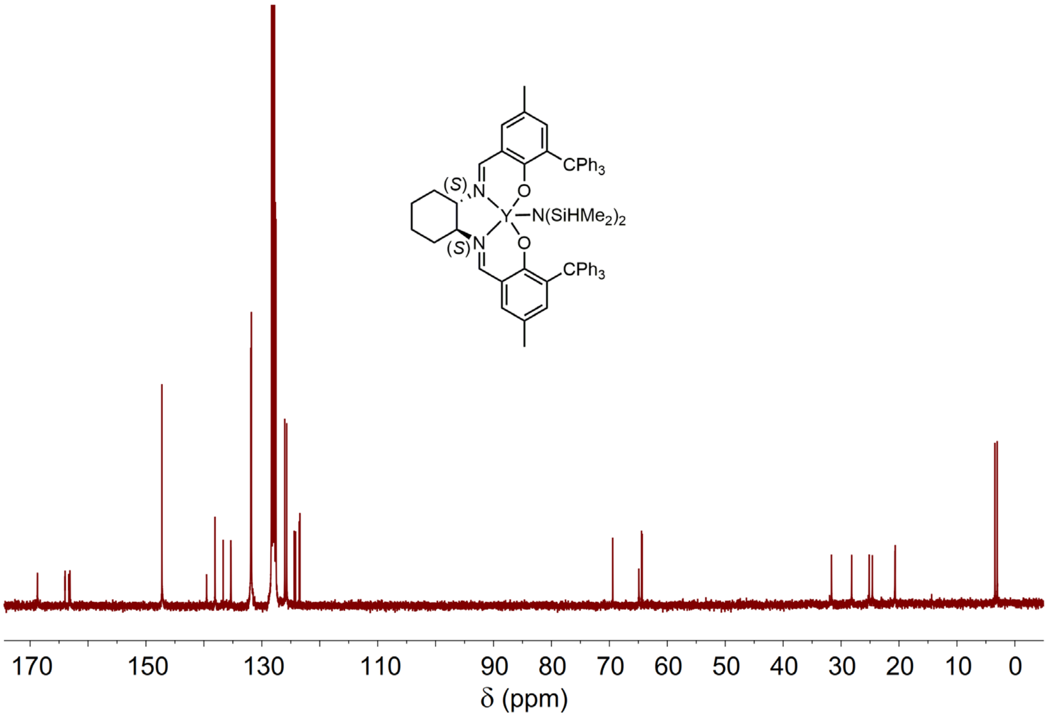

Supplementary Figure 29. ^{13}C NMR spectrum of yttrium complex (*S,S*)-**4d** in C_6D_6.

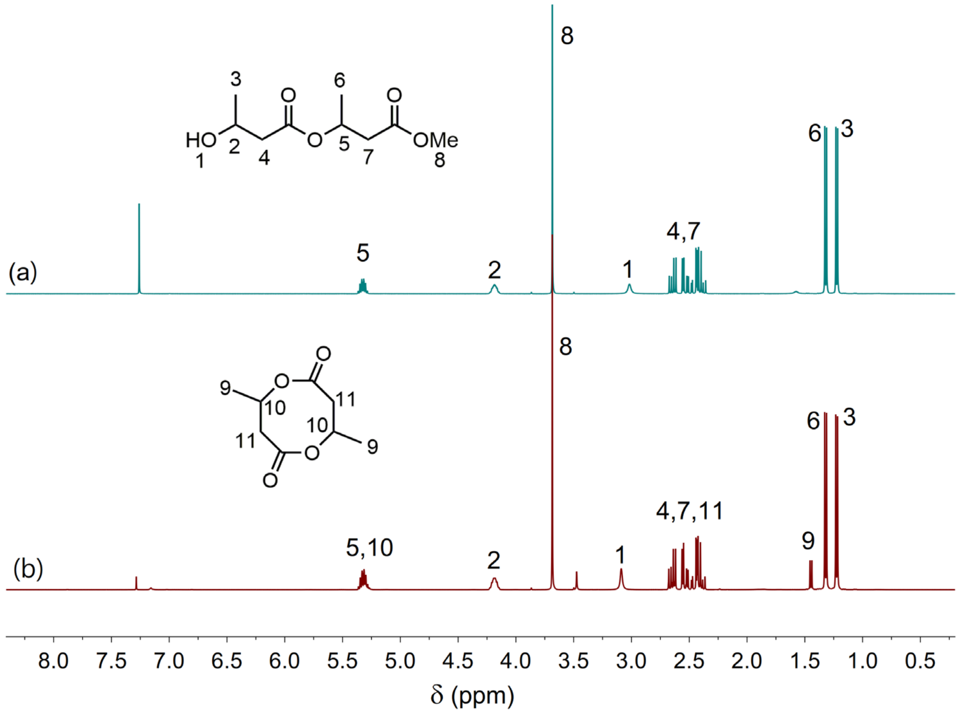

Supplementary Figure 30. ^{1}H NMR ($CDCl_3$) spectra of polymerization reaction filtrate quenched by (a) acidified MeOH; (b) MeOH.

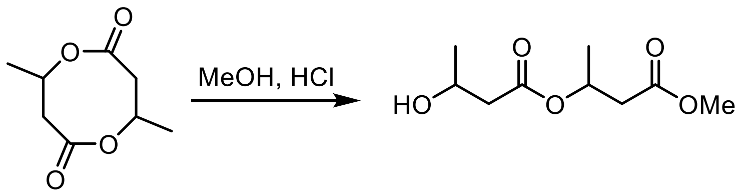

Supplementary Figure 31. Ring-opening reaction of DL with acidified methanol.

Supplementary Table 1. Results of *rac*-DL polymerization by La[N(SiMe$_3$)$_2$]$_3$ (**1**) and bisphenolate yttrium complexes (**2a**, **2b**, and **3**) at room temperature [a]

Run	Catalyst (cat)	Initiator (I)	[*rac*-DL] /[cat]/[I]	Time (h)	Conv. [b] (%)	M_n [c] (kg mol^{-1})	$Đ$ [c] (M_w/M_n)	P_m [d]	[*mm*] [d] (%)
1	**1**	BnOH	20/1/3	8	98	2.43	1.09	0.70	59
1	**1**	BnOH	20/1/2	4	100	n.d.	n.d.	0.74	63
2	**2a**	BnOH	20/1/1	48	17	n.d.	n.d.	n.d.	n.d.
3	**2b**	BnOH	20/1/1	48	44	n.d.	n.d.	n.d.	n.d.
4	**3**	BnOH	20/1/1	32	76	2.70	1.08	0.76	66

[a] Conditions: *rac*-DL = 0.138 g (0.8 mmol), [*rac*-DL] = 1.0 M, DCM as the solvent, $V_{solvent}$ = 0.8 mL, the catalyst and initiator amount varied according to the [*rac*-DL]/[cat]/[I] ratio. [b] Monomer conversions measured by ^{1}H NMR spectra of the quenched solution in benzoic acid/chloroform. [c] Number-average molecular weights (M_n) and dispersity indices ($Đ = M_w/M_n$) determined by GPC carried out at 40 ºC and a flow rate of 0.8 mL min^{-1}, with chloroform as the eluent on a Viscotek GPCmax VE 2001 instrument equipped with one PLgel 5 μm guard and three PLgel 5 μm mixed-C columns (Polymer Laboratories; linear range of MW = 200–2,000,000). The instrument was calibrated with 10 PMMA standards, and chromatograms were processed with Malvern OmniSEC software (version 4.7). [d] P_m is the probability of *meso* linkages between HB units, and *mm* is isotactic triad made up of two adjacent *meso* diads, determined by ^{13}C{^{1}H} NMR spectroscopy.

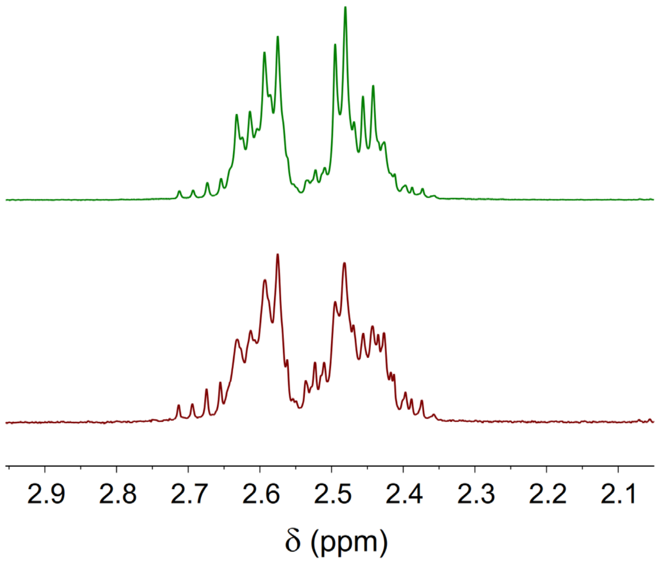

Supplementary Figure 32. Overlay of ^{1}H NMR spectra ($CDCl_3$) of P3HB in the methylene region produced by **1**/2BnOH (top) and **1**/3BnOH (bottom).

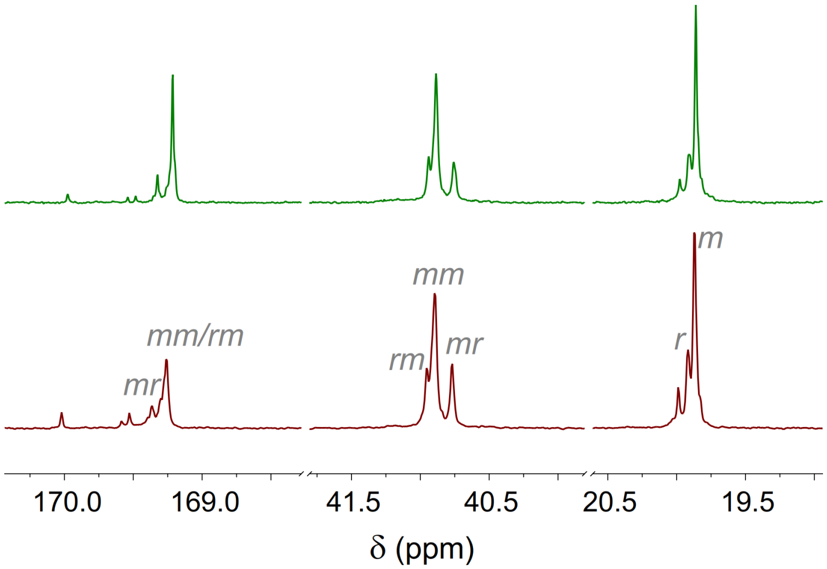

Supplementary Figure 33. Overlay of ^{13}C NMR spectra ($CDCl_3$) of P3HB in the carbonyl, methylene, and methyl regions produced by **1**/2BnOH (top) and **1**/3BnOH (bottom).

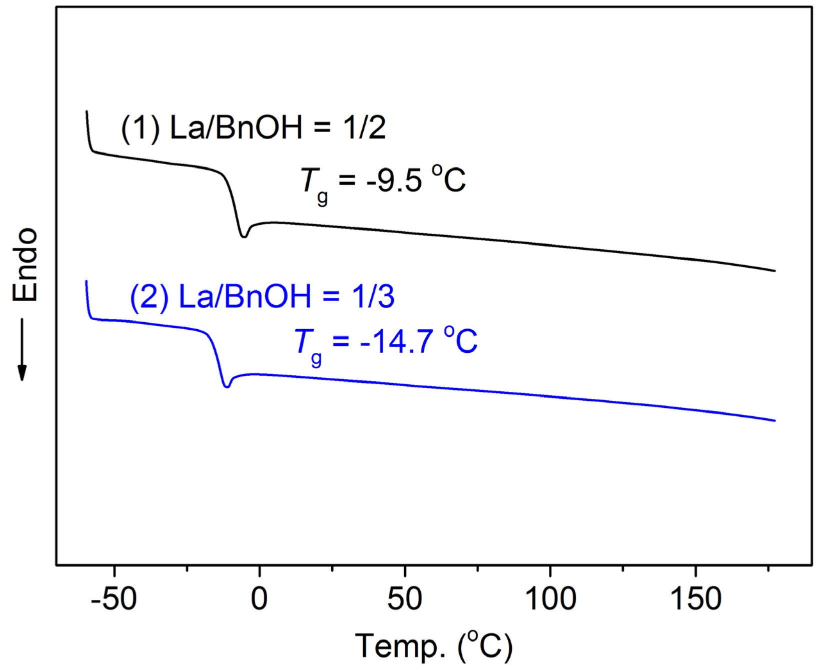

Supplementary Figure 34. DSC curves of P3HB by (1) **1**/2BnOH and (2) **1**/3BnOH.

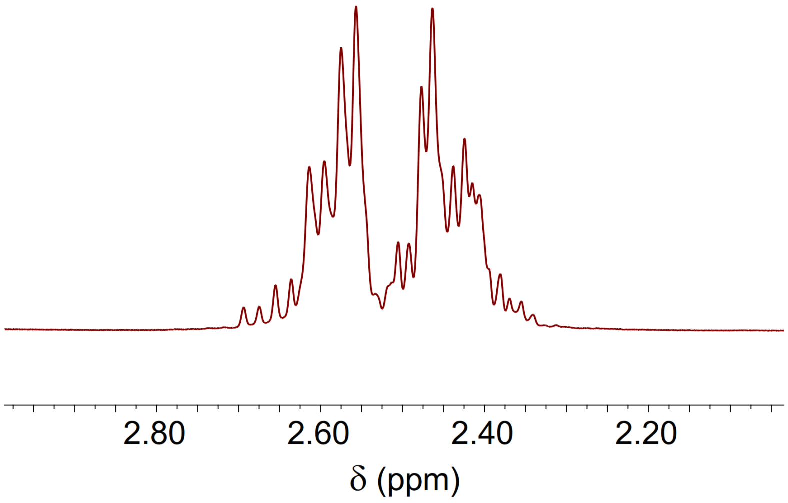

Supplementary Figure 35. ^{1}H NMR spectrum ($CDCl_3$) of P3HB in the methylene region produced by catalyst **3**.

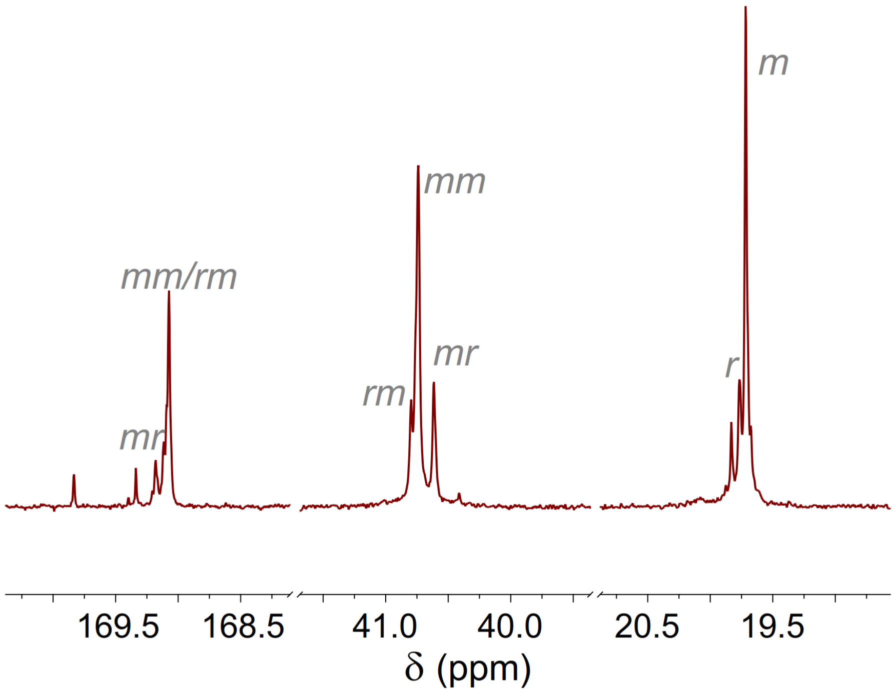

Supplementary Figure 36. ^{13}C NMR spectrum ($CDCl_3$) of P3HB in the carbonyl, methylene, and methyl regions produced by catalyst **3**.

Supplementary Note 1: The crystallinity of the resulting P3HB was calculated using the equation X_c (%) = $(\Delta H_f/\Delta H_f^0) \times 100$, where ΔH_f and ΔH_f^0 is the heat of fusion (J g^{-1}) of the synthesized P3HB and the 100% crystalline P3HB (146 J g^{-1}),[14] respectively. Assignments of P3HB tacticities or stereo-microstructures were made through analysis of polymer samples by ^1H and ^{13}C NMR, following the established literature assignments and procedures.[15-18]

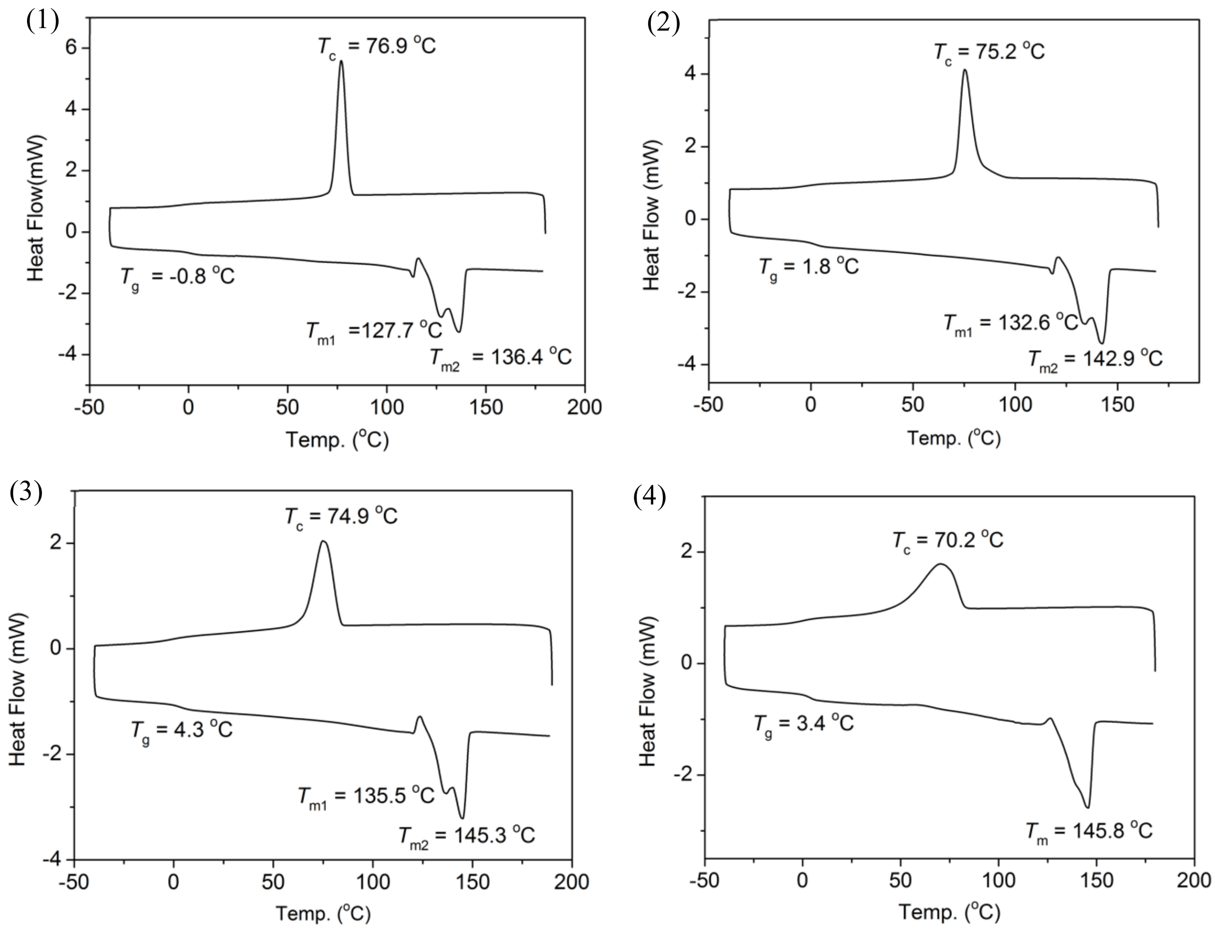

Supplementary Figure 37. DSC curves of P3HB produced by **4a**/BnOH with [*rac*-DL]/[**4a**] of: (1) 20/1 (ΔH = 57.5 J g^{-1}); (2) 50/1 (ΔH = 52.0 J g^{-1}); (3) 100/1 (ΔH = 47.0 J g^{-1}); and (4) 200/1 (ΔH = 40.9 J g^{-1}). The cooling and second heating rate was 5 °C min^{-1}.

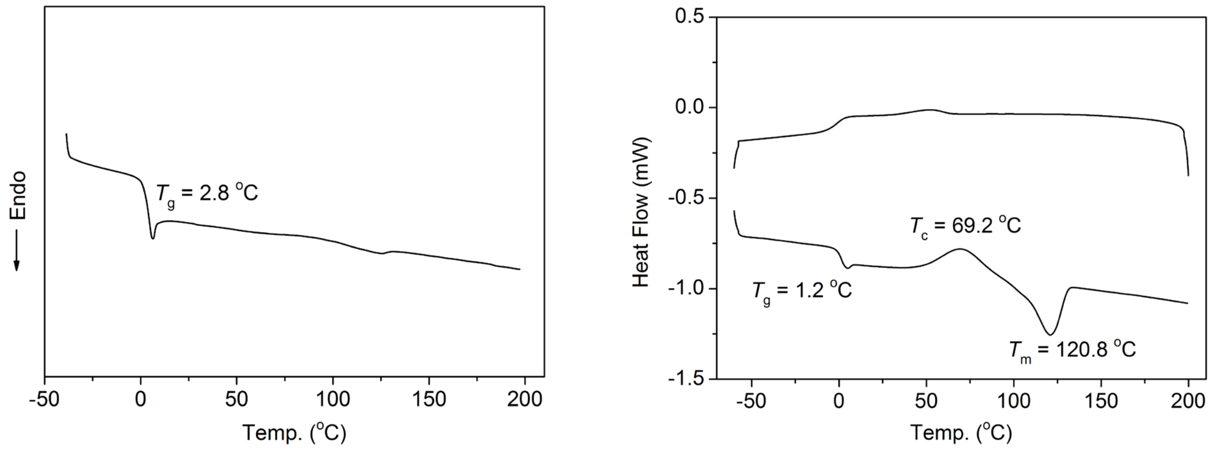

Supplementary Figure 38. DSC curves of P3HB produced by [*rac*-DL]/[**4e**] = 100/1 with different cooling and second heating rates: at 10 °C min^{-1} (left column); 2 °C min^{-1} (right column).

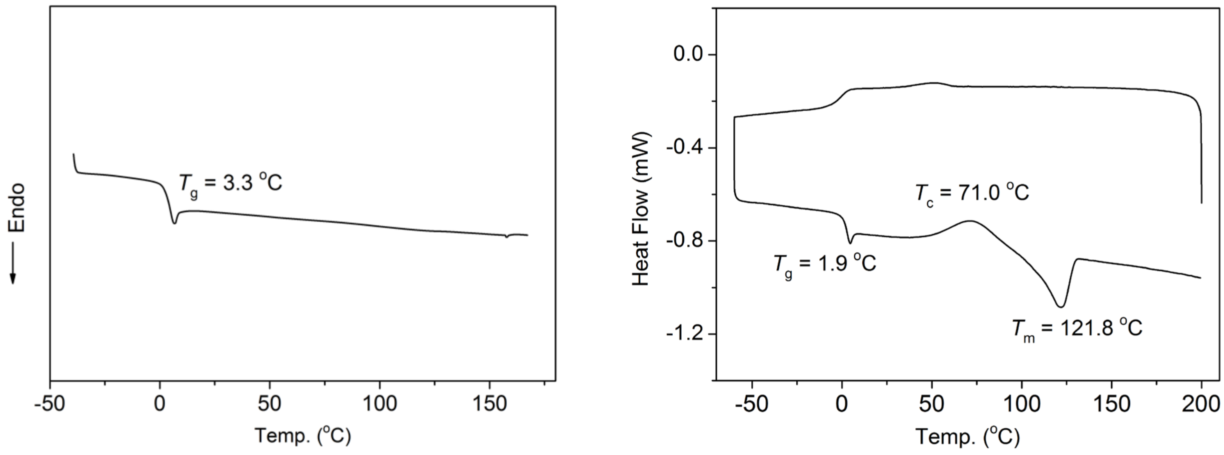

Supplementary Figure 39. DSC curves of P3HB produced by [*rac*-DL]/[**4e**] = 200/1 with different cooling and second heating rates: at 10 °C min^{-1} (left column); at 2 °C min^{-1} (right column).

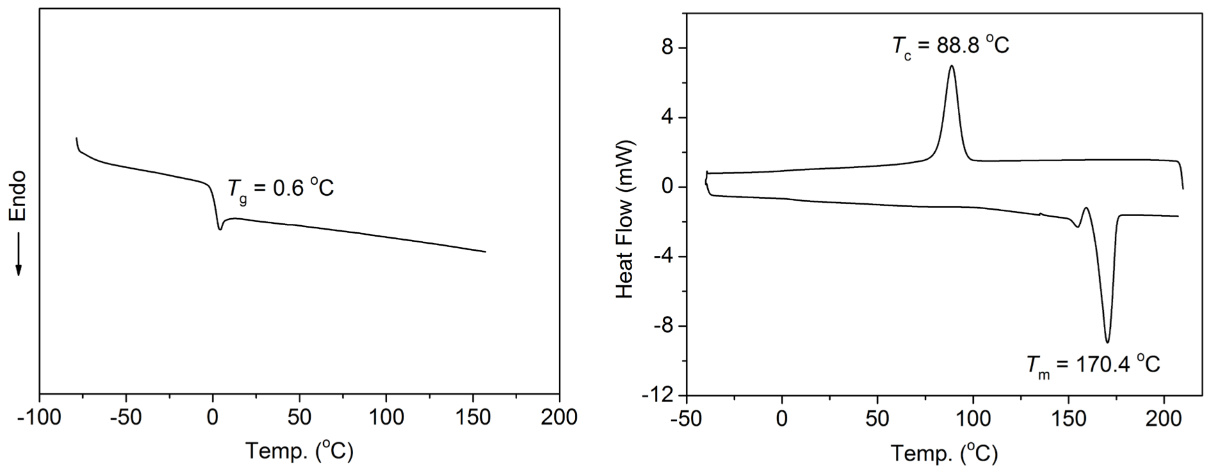

Supplementary Figure 40. DSC curves of amorphous, atactic P3HB produced via ROP of *β*-BL by **4d** (left) and perfectly isotactic, highly crystalline P3HB produced by **4d** with [*rac*-DL]/[**4d**] = 800/1 (ΔH_f = 78.1 J g^{-1}). The cooling and second heating rate was 10 °C min^{-1}.

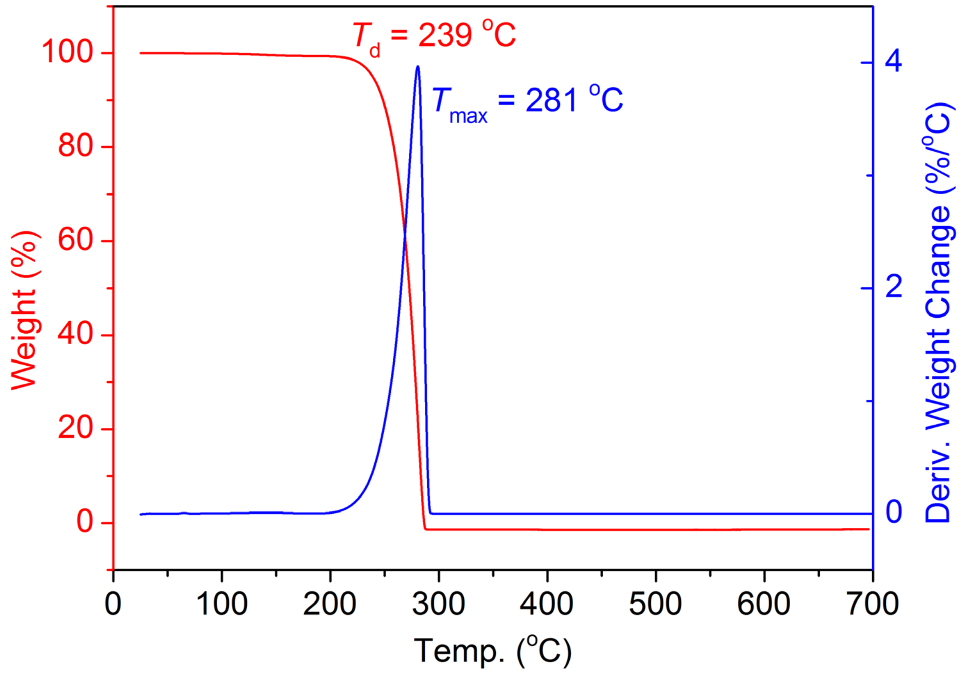

Supplementary Figure 41. TGA and DTG curves of P3HB produced by [*rac*-DL]/[**4d**] = 200/1 (M_n = 3.74 × 10^4 g mol^{-1}, *Đ* = 1.07, [*mm*] > 99%).

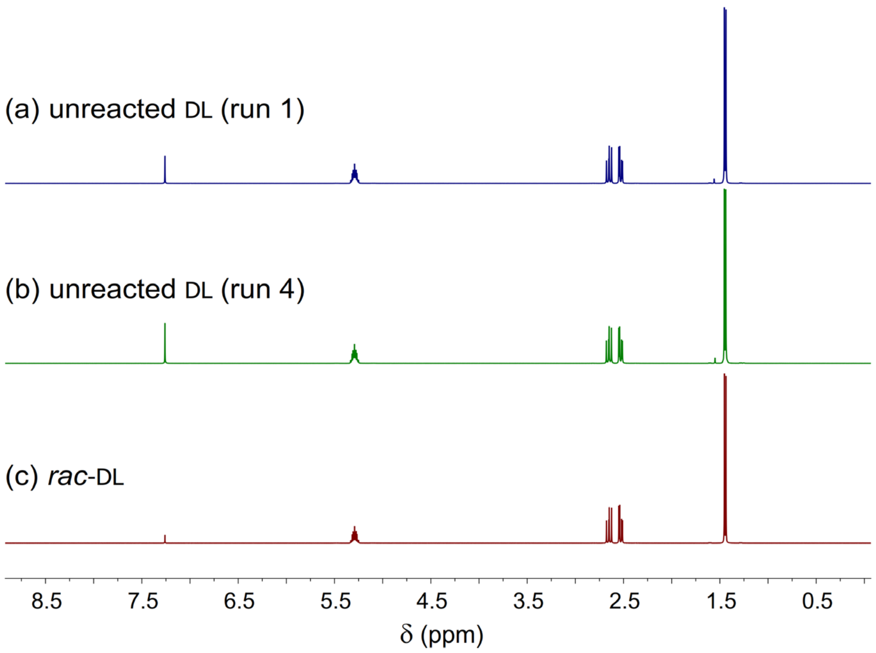

Supplementary Figure 42. Overlay of ^{1}H NMR spectra of (c) *rac*-DL and unreacted DL by (a) (*R,R*)**-4d** (run 1, Table 2), (b) (*S,S*)**-4d** (run 4, Table 2).

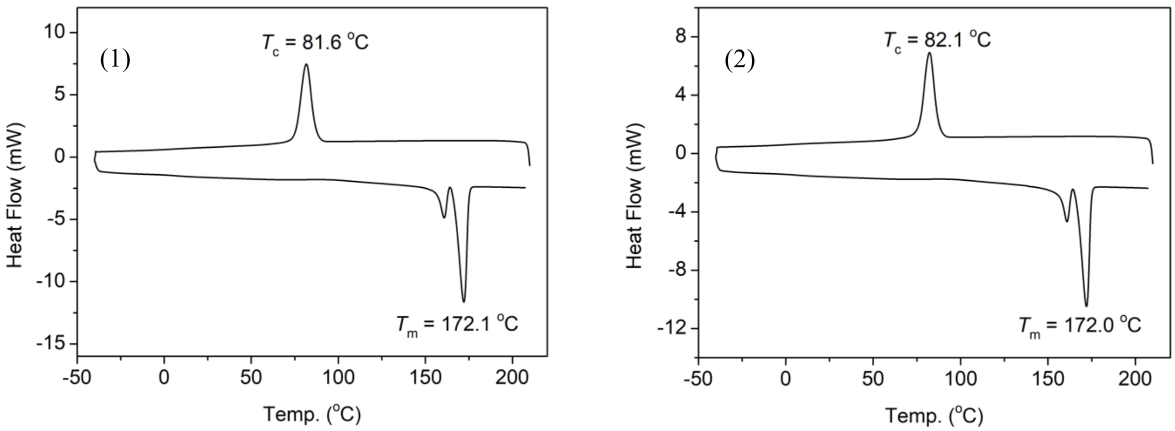

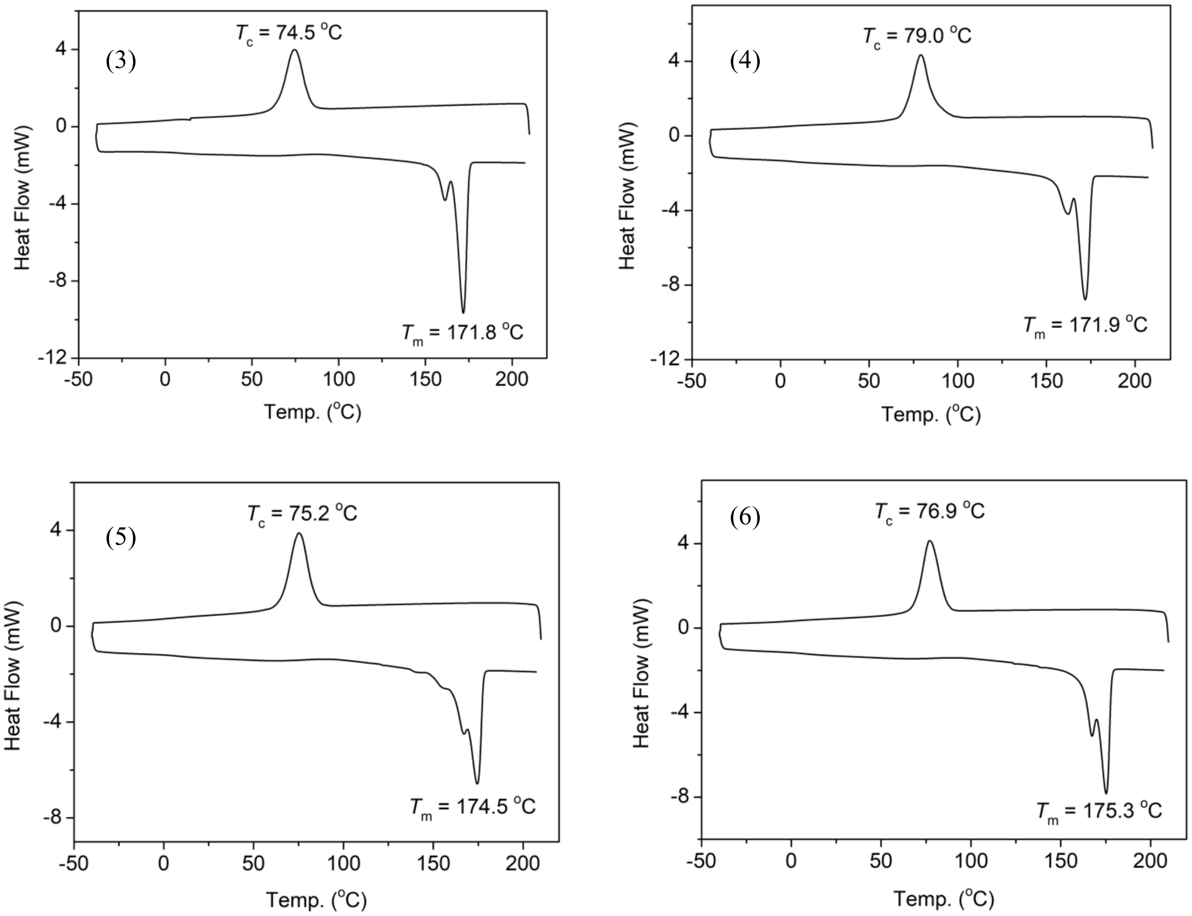

Supplementary Figure 43. DSC curves of enantiomeric P3HB by (*R,R*)-**4d** and (*S,S*)-**4d**. (1) [*rac*-DL]/[(*R,R*)-**4d**] = 400/1 (ΔH_f = 84.8 J g^{-1}). (2) [*rac*-DL]/[(*S,S*)-**4d**] = 400/1 (ΔH_f = 78.9 J g^{-1}). (3) [*rac*-DL]/[(*R,R*)-**4d**] = 800/1 (ΔH_f = 87.7 J g^{-1}). (4) [*rac*-DL]/[(*S,S*)-**4d**] = 800/1 (ΔH_f = 87.4 J g^{-1}). (5) [*rac*-DL]/[(*R,R*)-**4d**] = 1600/1 (ΔH_f = 88.2 J g^{-1}). (6) [*rac*-DL]/[(*S,S*)-**4d**] = 1600/1 (ΔH_f = 82.5 J g^{-1}). Crystallization temperature (T_c) and melting-transition temperature (T_m) taken from the cooling and second heating scans, respectively.

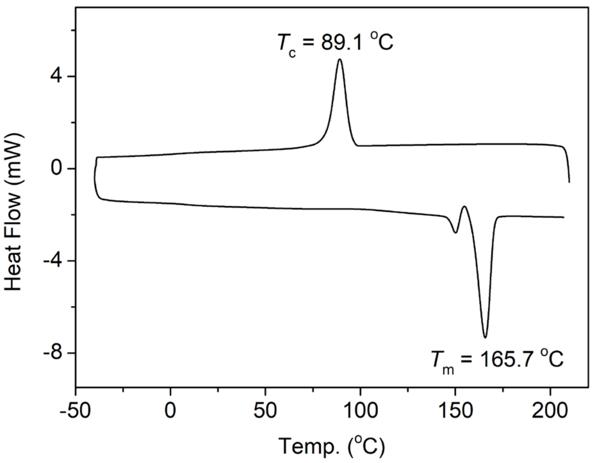

Supplementary Figure 44. DSC curve of the 1:1 mixture of P(*R*-3HB) and P(*S*-3HB) produced by (*S,S*)-**4d** and (*R,R*)-**4d (**mixture of runs 1 and 4 in Table 2, ΔH_f = 91.2 J g^{-1}).

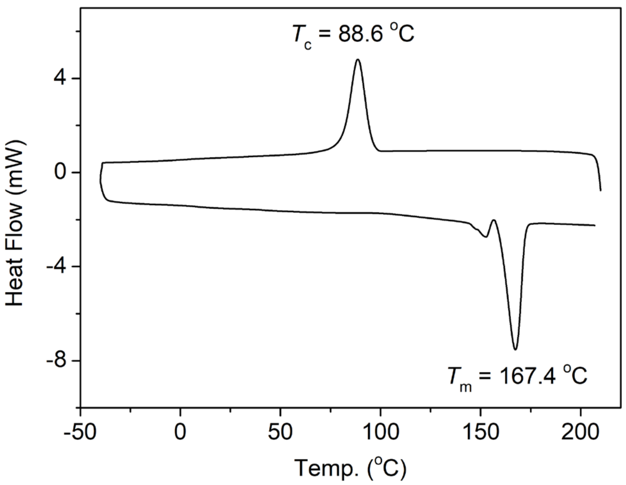

Supplementary Figure 45. DSC curve of the 1:1 mixture of P(*R*-3HB) and P(*S*-3HB) produced by (*S,S*)-**4d** and (*R,R*)-**4d (**mixture of runs 2 and 5 in Table 2, ΔH_f = 87.2 J g^{-1}).

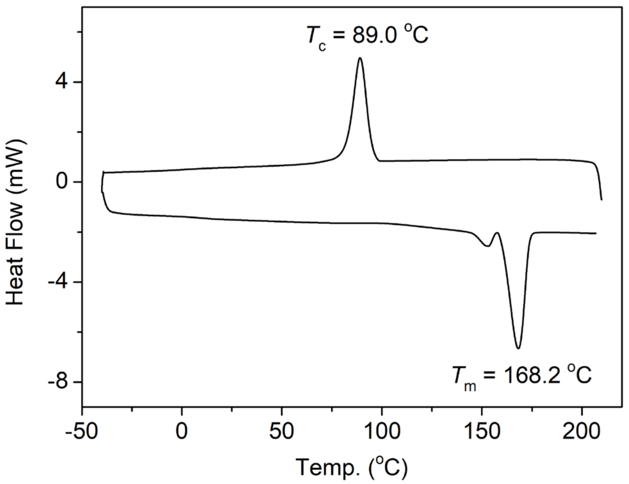

Supplementary Figure 46. DSC curve of the 1:1 mixture of P(*R*-3HB) and P(*S*-3HB) produced by (*S,S*)-**4d** and (*R,R*)-**4d** (mixture of runs 3 and 6 in Table 2, ΔH_f = 92.5 J g^{-1}).

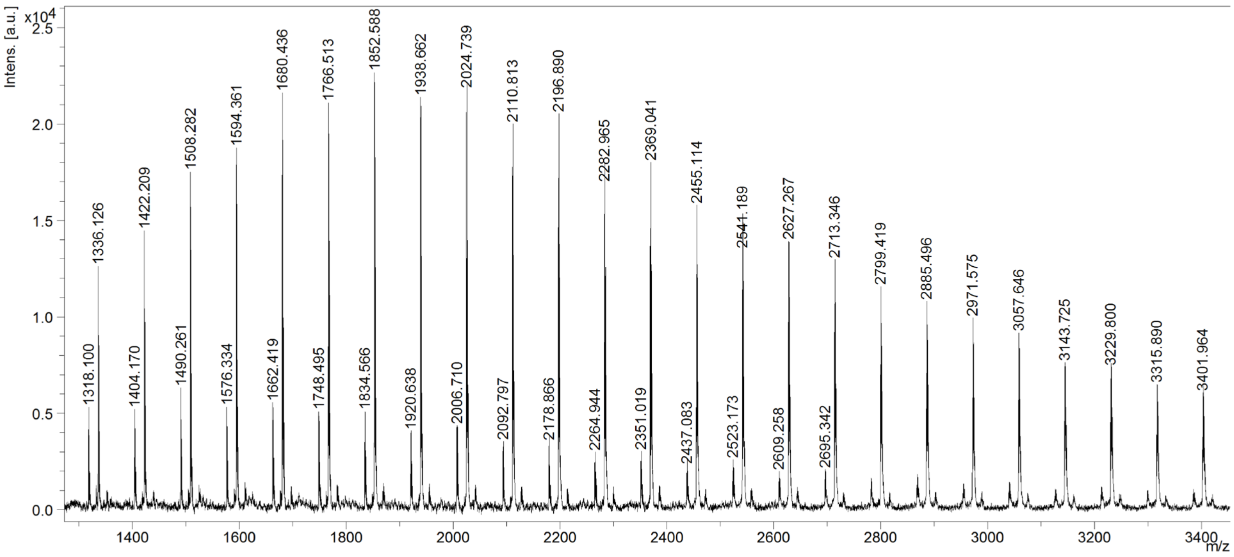

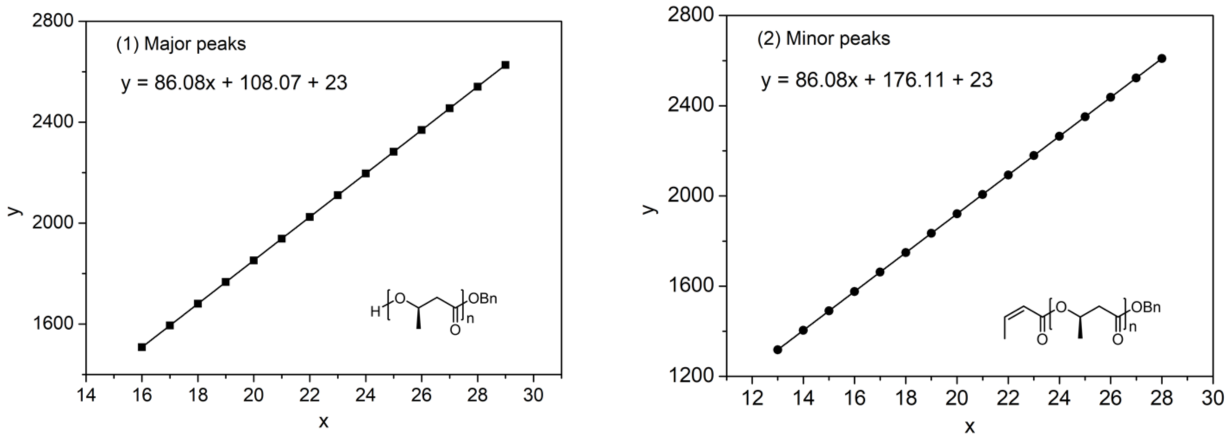

Supplementary Figure 47. MALDI-TOF spectrum of P3HB produced with *rac*-DL/*rac*-**4d**/BnOH (20/1/1, 20 min) and plots of *m*/*z* values (y) *vs* the number of *rac*-DL repeat units (x).

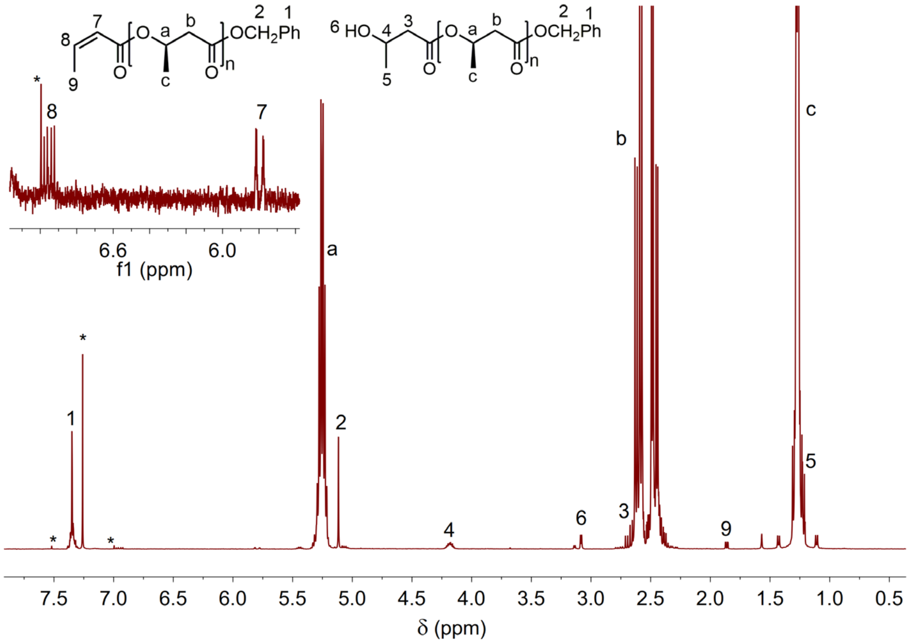

Supplementary Figure 48. ^{1}H NMR spectrum of P3HB produced with *rac*-DL/*rac*-**4d**/BnOH (20/1/1, 20 min; *$CHCl_3$).

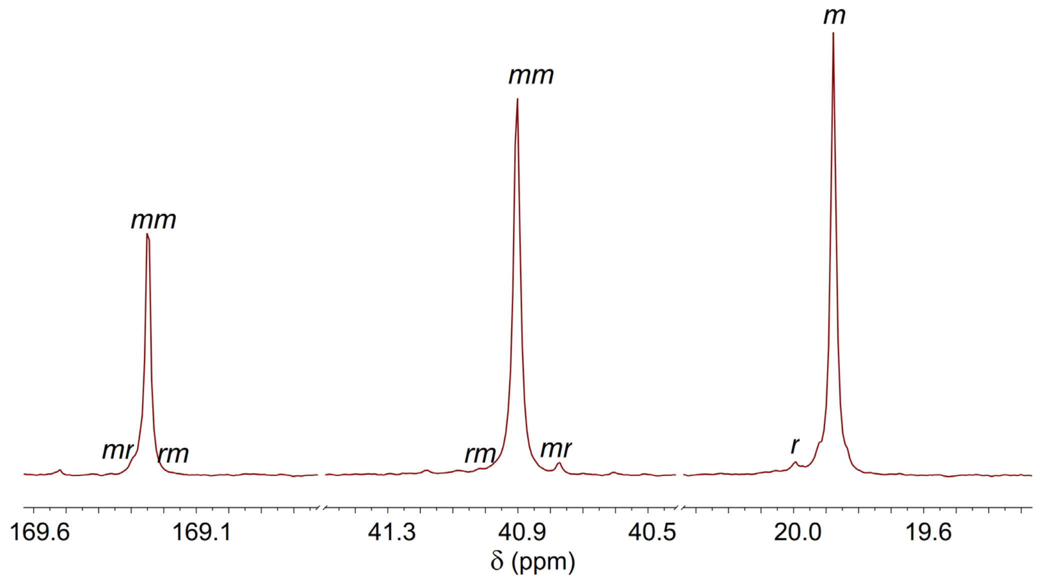

Supplementary Figure 49. ^{13}C NMR spectra ($CDCl_3$) in the carbonyl, methylene, and methyl regions of P3HB produced by [*rac*-DL]/[*rac*-**4d**] = 20/1 (polymerization time: 20 min).

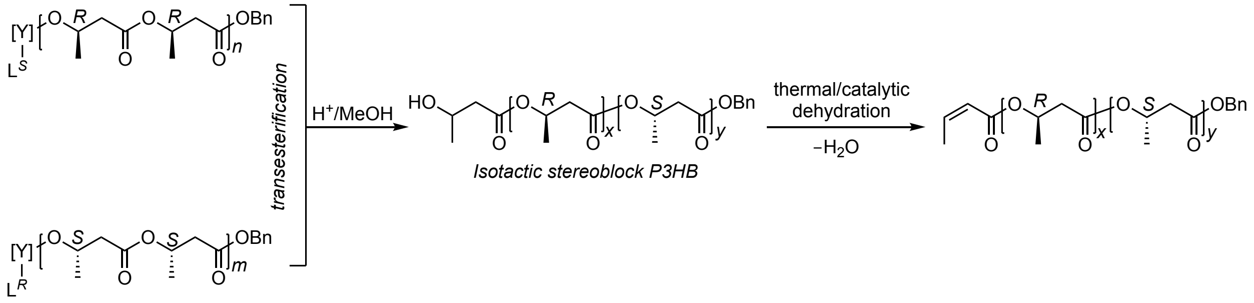

Supplementary Figure 50. Proposed transesterification side reaction occurring after the full monomer conversion has been achieved and the corresponding stereoblock P3HB structures.

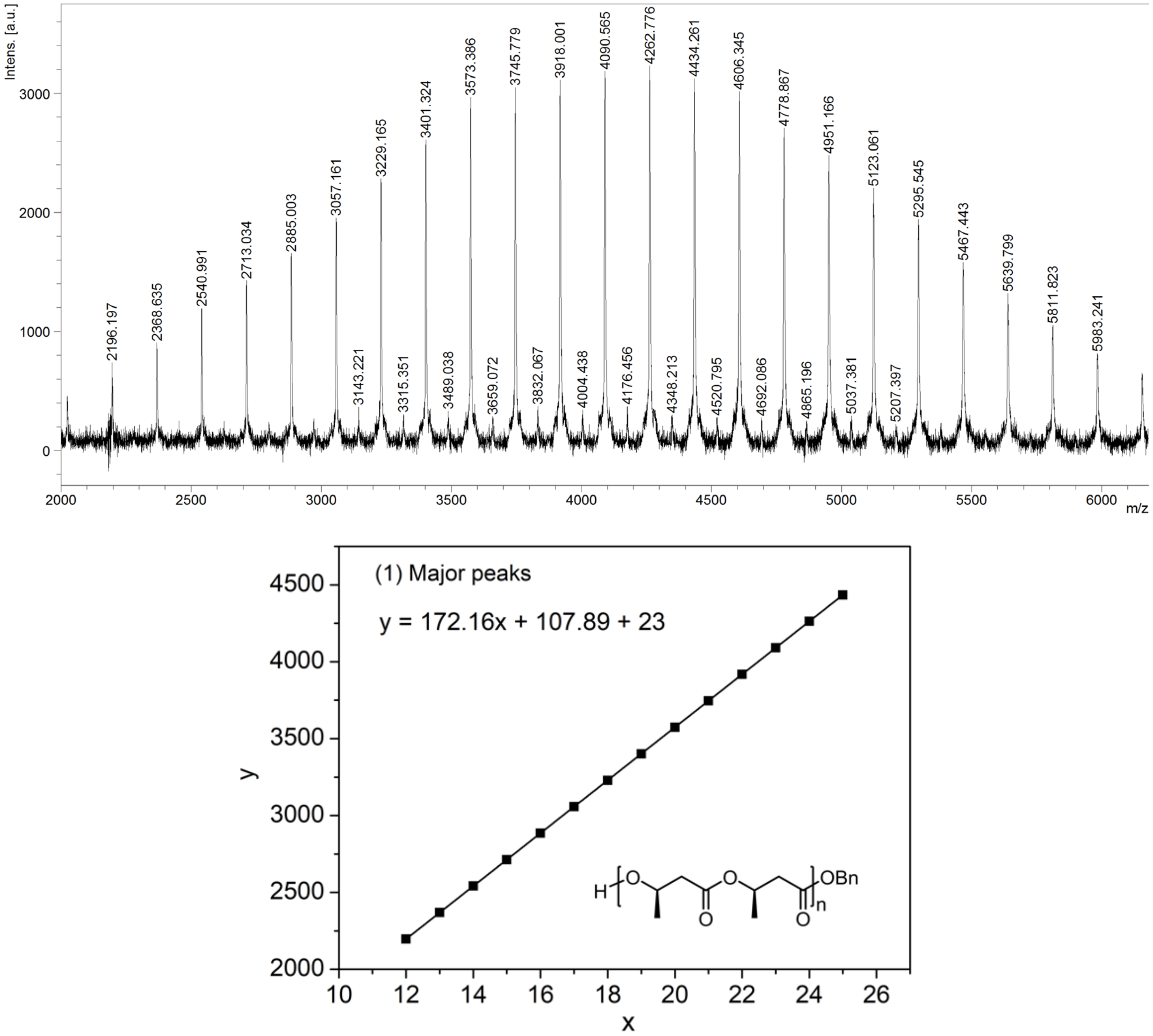

Supplementary Figure 51. MALDI-TOF spectrum of P3HB produced with *rac*-DL/*rac*-**4d**/BnOH (20/1/1, 30 s) and plots of *m*/*z* values (y) *vs* the number of *rac*-DL repeat units (x).

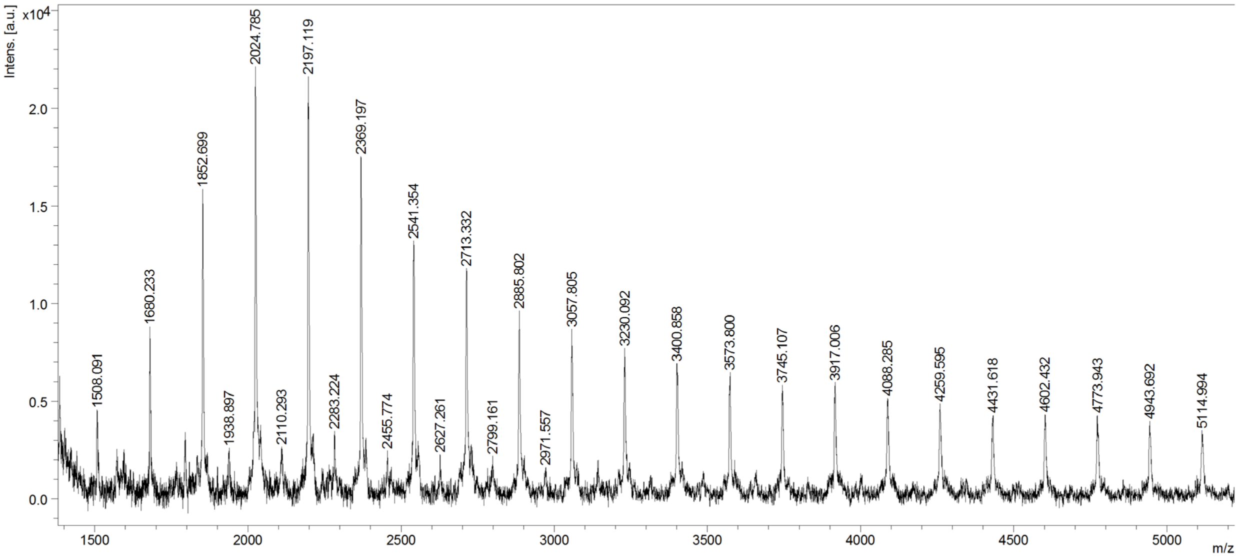

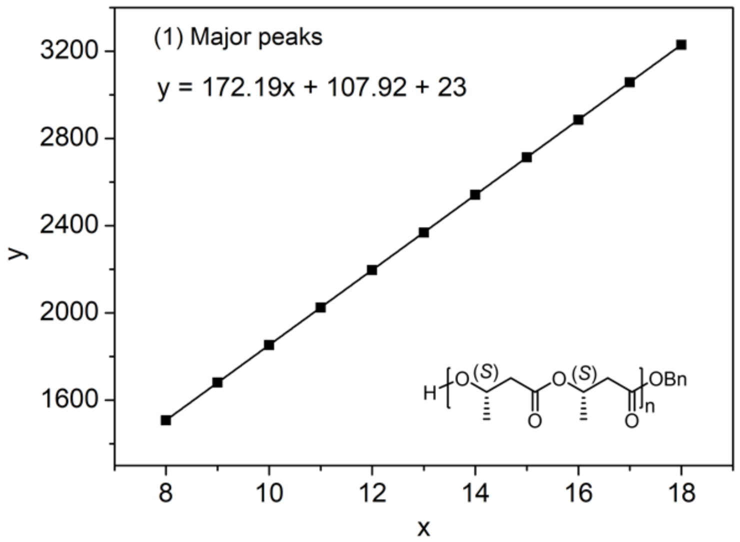

Supplementary Figure 52. MALDI-TOF spectrum of P3HB produced with *rac*-DL/(*R,R*)-**4d**/BnOH (400/1/1) and plot of *m*/*z* values (y) *vs* the number of DL repeat units (x).

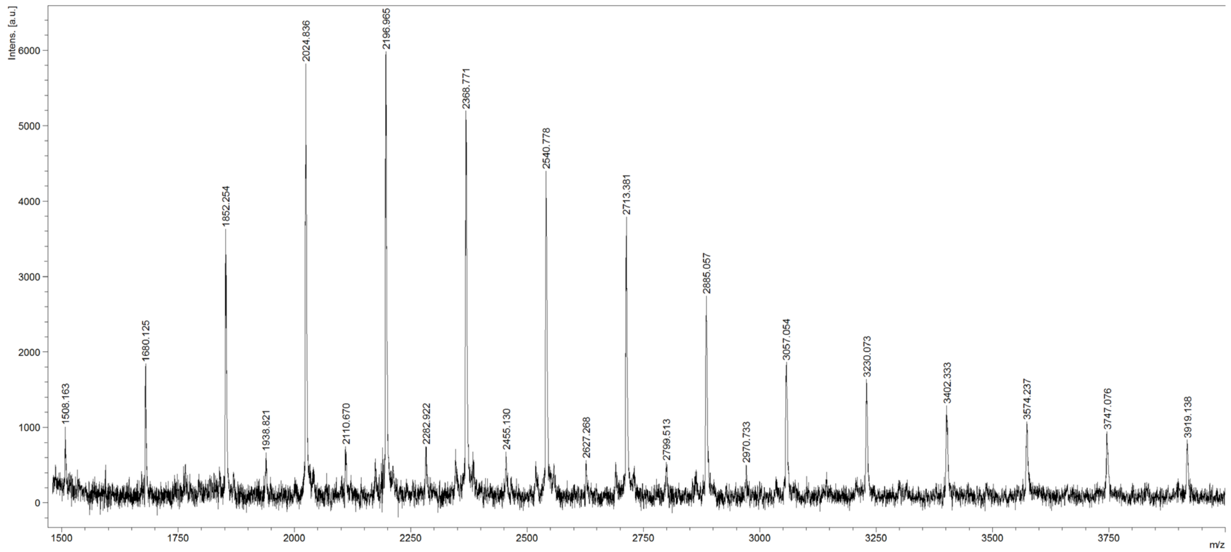

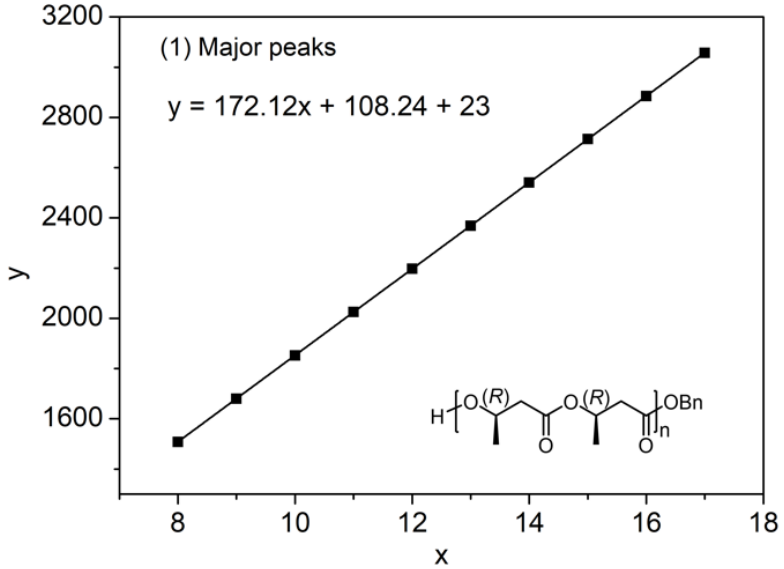

Supplementary Figure 53. MALDI-TOF spectrum of P3HB produced with *rac*-DL/(*S,S*)-**4d**/BnOH (400/1/1) and plot of *m*/*z* values (y) *vs* the number of DL repeat units (x).

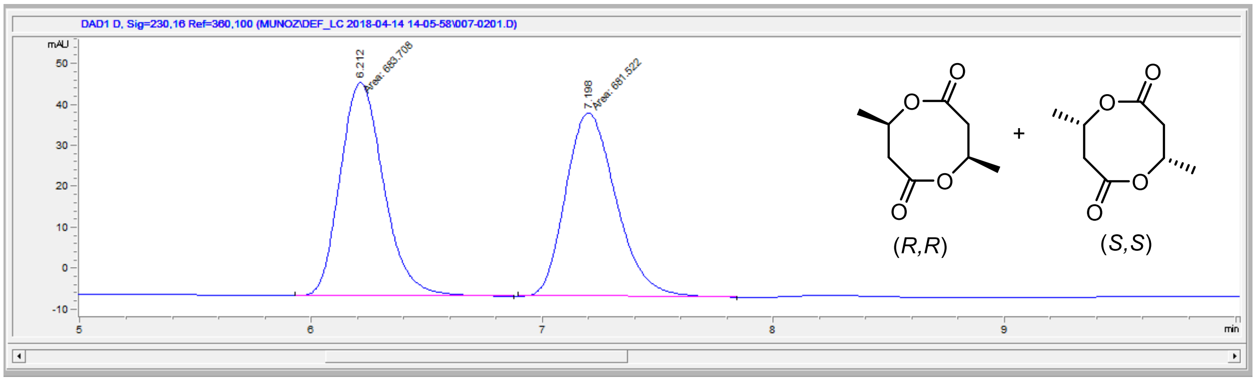

#	Time	Area	Height	Width	Area%	Symmetry
1	6.212	683.7	52.2	0.2184	50.080	0.847
2	7.198	681.5	44.9	0.2532	49.920	0.764

Supplementary Figure 54. HPLC chromatogram of *rac*-DL separated by chiral column into enantiomers in 1:1 ratio.

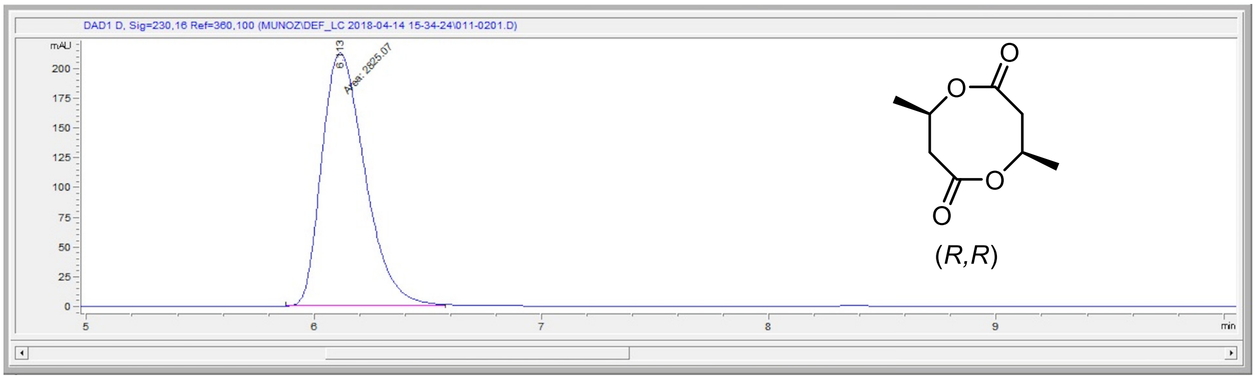

#	Time	Area	Height	Width	Area%	Symmetry
1	6.113	2825.1	213.4	0.2206	100.000	0.666

Supplementary Figure 55. HPLC chromatogram of the unreacted monomer from the polymerization of *rac*-DL (400 equiv) by (*R,R*)-**4d** (run 1, Table 2).

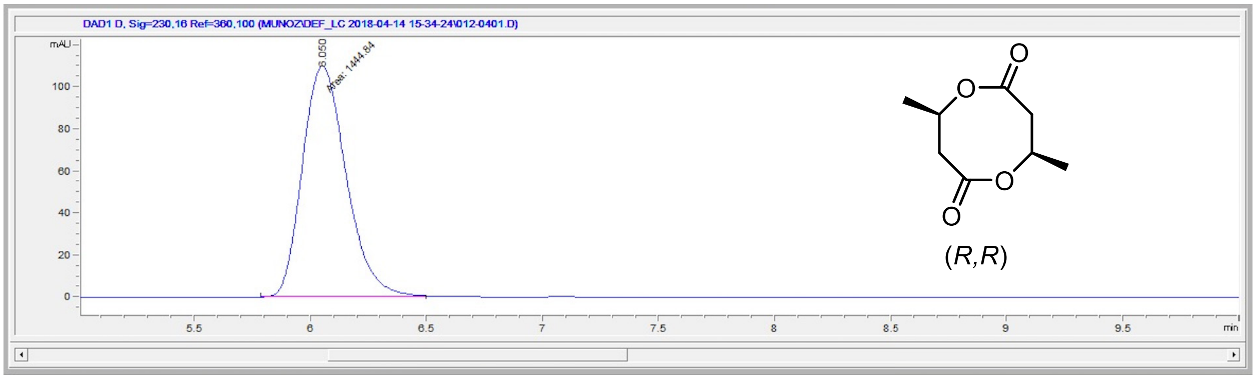

#	Time	Area	Height	Width	Area%	Symmetry
1	6.05	1444.8	110.2	0.2186	100.000	0.781

Supplementary Figure 56. HPLC chromatogram of the unreacted monomer from the polymerization of *rac*-DL (800 equiv) by (*R,R*)-**4d** (run 2, Table 2).

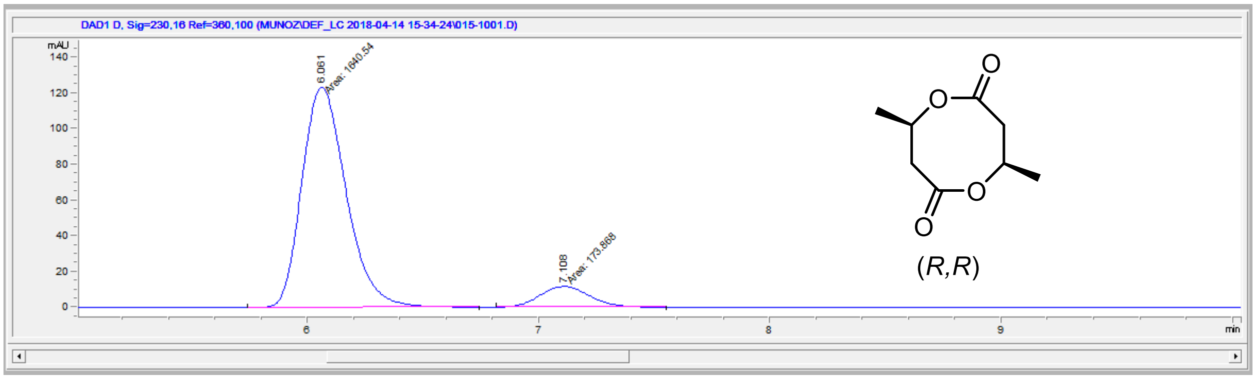

#	Time	Area	Height	Width	Area%	Symmetry
1	6.061	1640.5	123.4	0.2216	90.417	0.823
2	7.108	173.9	11.6	0.2494	9.583	0.934

Supplementary Figure 57. HPLC chromatogram of the unreacted monomer from the polymerization of *rac*-DL (1600 equiv) by (*R,R*)-**4d** (run 3, Table 2).

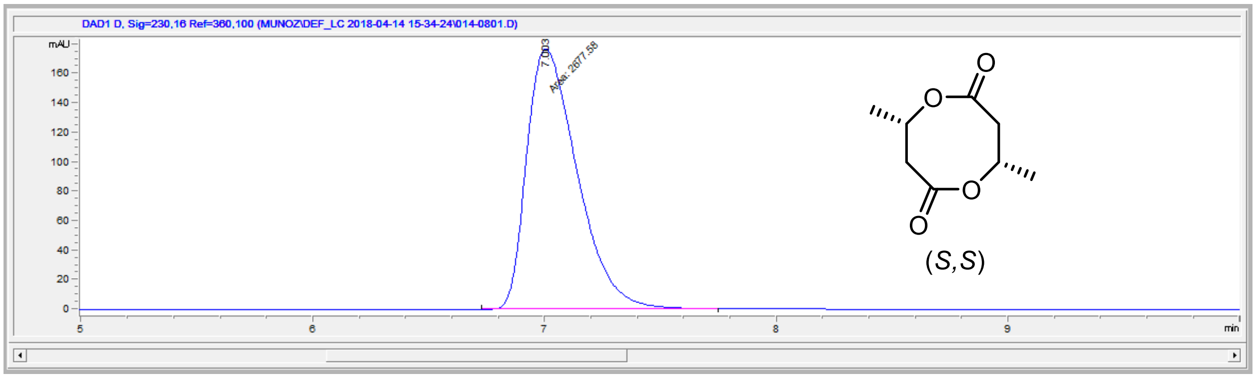

#	Time	Area	Height	Width	Area%	Symmetry
1	7.003	2677.6	176.7	0.2526	100.000	0.57

Supplementary Figure 58. HPLC chromatogram of the unreacted monomer from the polymerization of *rac*-DL (400 equiv) by (*S*,*S*)-**4d** (run 4, Table 2).

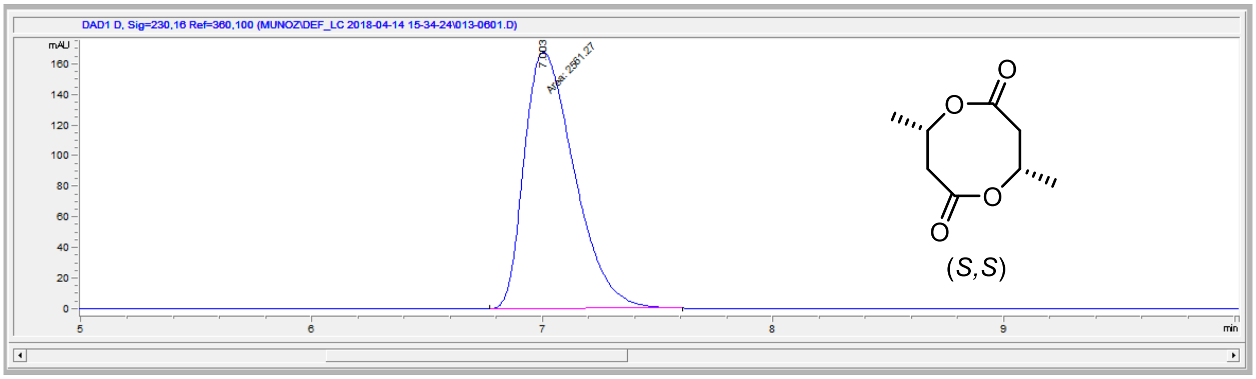

#	Time	Area	Height	Width	Area%	Symmetry
1	7.003	2561.3	168.6	0.2531	100.000	0.591

Supplementary Figure 59. HPLC chromatogram of the unreacted monomer from the polymerization of *rac*-DL (800 equiv) by (*S*,*S*)-**4d** (run 5, Table 2).

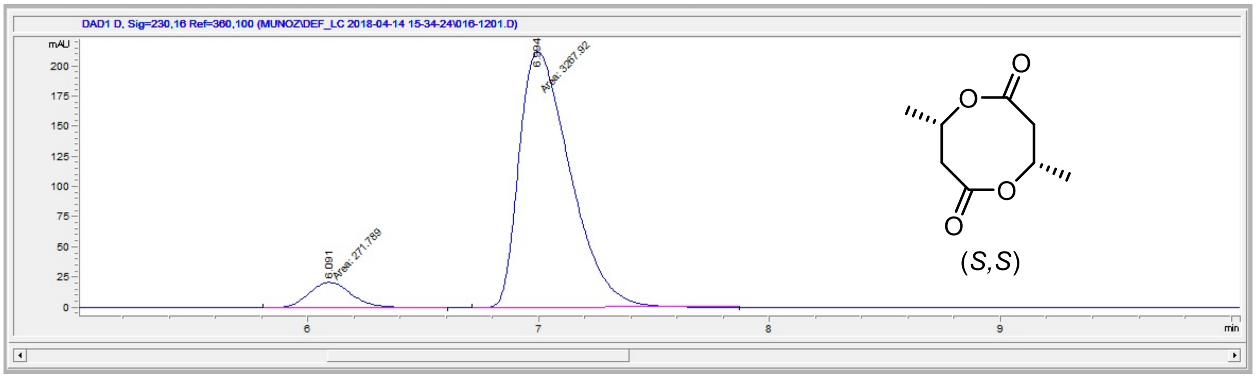

#	Time	Area	Height	Width	Area%	Symmetry
1	6.091	271.8	21.1	0.2147	7.678	0.84
2	6.994	3267.9	212.6	0.2561	92.322	0.551

Supplementary Figure 60. HPLC chromatogram of the unreacted monomer from the polymerization of *rac*-DL (1600 equiv) by (*S,S*)-**4d** (run 6, Table 2).

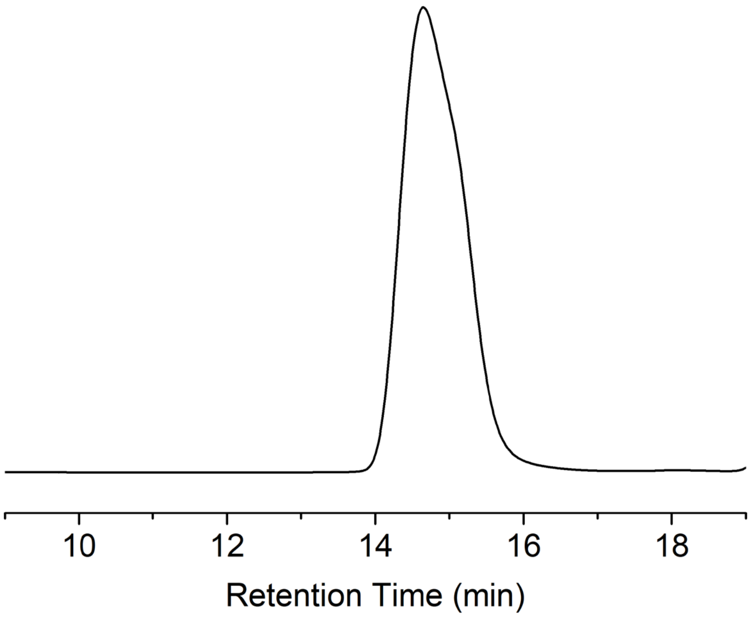

Supplementary Figure 61. GPC trace of P3HB by [*rac*-DL]/[*rac*-**4d**] = 100/1 (M_n = 20.1 kg mol^{-1}, $Đ$ = 1.07).

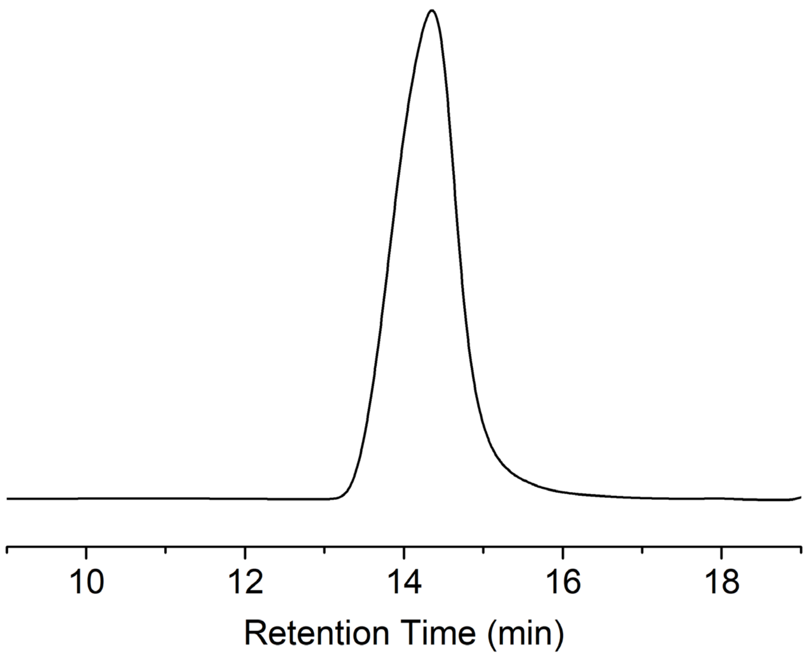

Supplementary Figure 62. GPC trace of P3HB by [*rac*-DL]/[*rac*-**4d**] = 200/1 (M_n = 37.4 kg mol^{-1}, $Đ$ = 1.07).

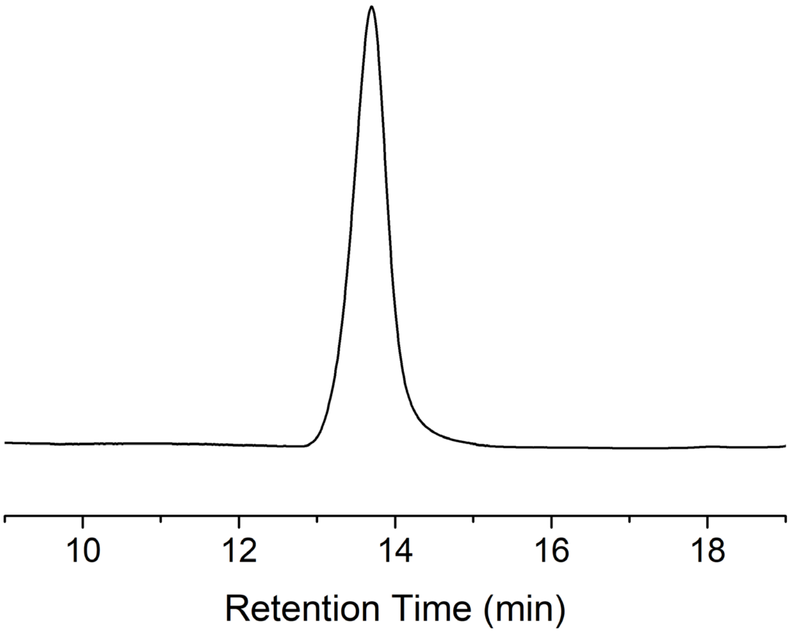

Supplementary Figure 63. GPC trace of P3HB by [*rac*-DL]/[*rac*-**4d**] = 400/1 (M_n = 64.3 kg mol^{-1}, $Đ$ = 1.02).

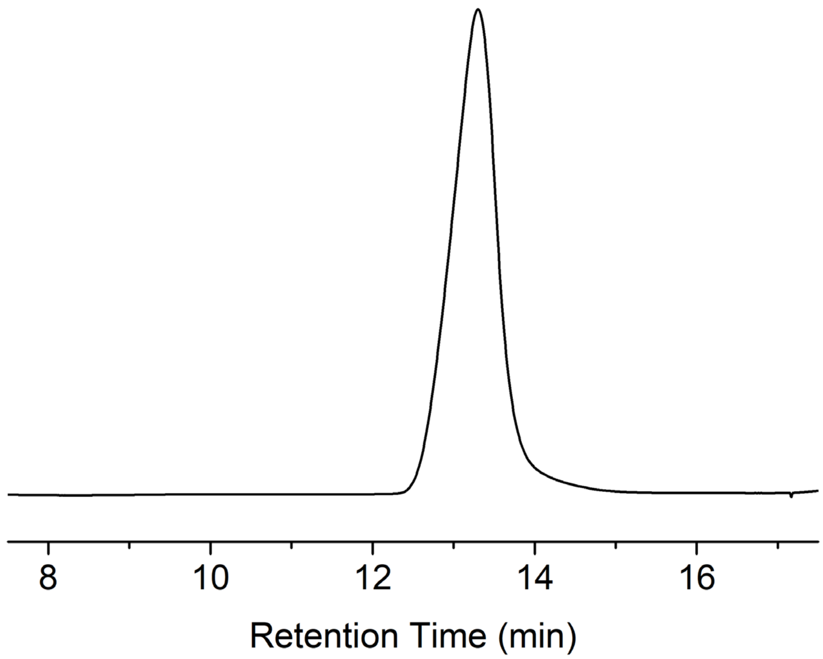

Supplementary Figure 64. GPC trace of P3HB by [*rac*-DL]/[*rac*-**4d**] = 800/1 (M_n = 119 kg mol^{-1}, $Đ$ = 1.03).

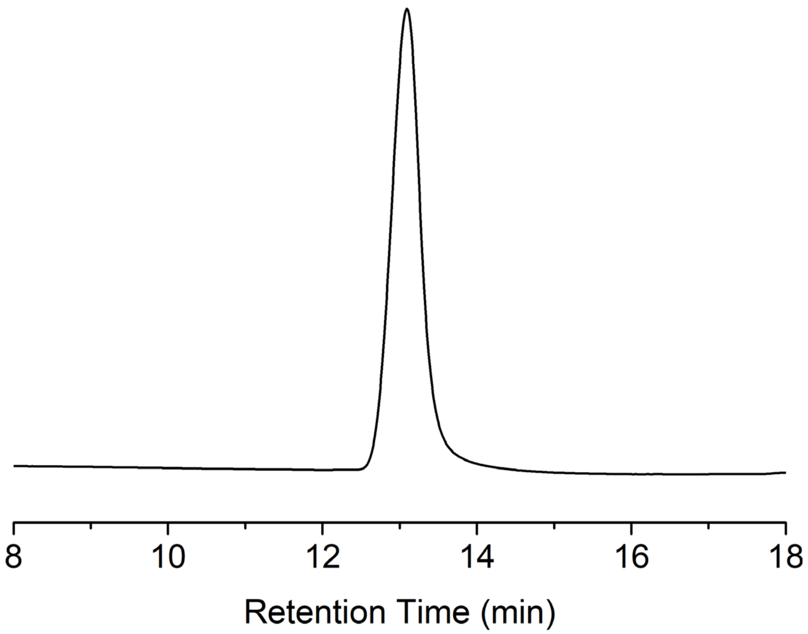

Supplementary Figure 65. GPC trace of P3HB by [*rac*-DL]/[*rac*-**4d**] = 1200/1 (M_n = 133 kg mol^{-1}, $Đ$ = 1.01).

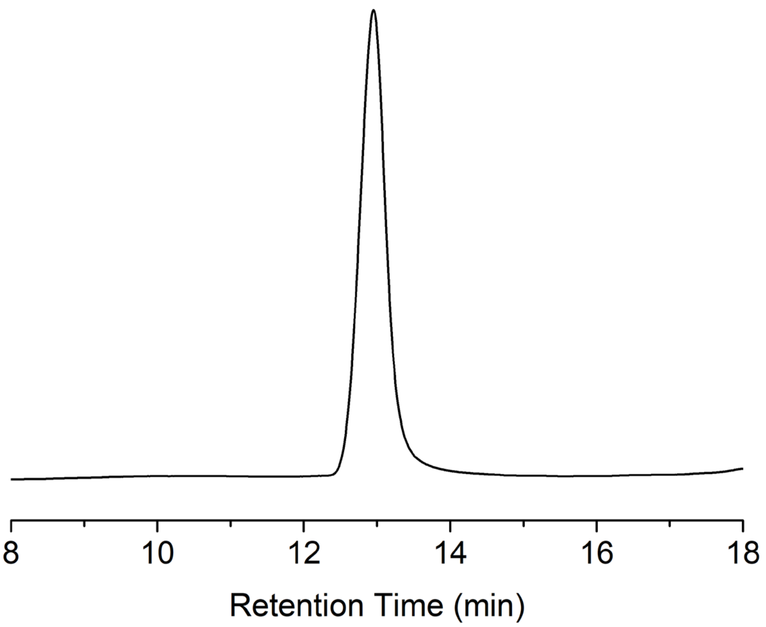

Supplementary Figure 66. GPC trace of P3HB by [*rac*-DL]/[*rac*-**4d**] = 1200/1 ([*rac*-DL] = 2.0 mol L^{-1}, M_n = 154 kg mol^{-1}, $Đ$ = 1.01).

Supplementary References

1. Anwander, R.; Runte, O.; Eppinger, J.; Gerstberger, G.; Herdtweck, E.; Spiegler, M. Synthesis and structural characterisation of rare-earth bis(dimethylsilyl)amides and their surface organometallic chemistry on mesoporous MCM-41. *J, Chem. Soc. Dalton Trans.* 847−858 (1998).
2. Eppinger, J.; Herdtweck, E.; Anwander, R. Synthesis and characterization of alkali metal bis(dimethylsilyl) amides: infinite all-planar laddering in the unsolvated sodium derivative. *Polyhedron* **17**, 1195−1201 (1998).
3. Amgoune, A.; Thomas, C. M.; Roisnel, T.; Carpentier, J.-F. Ring-opening polymerization of lactide with group 3 metal complexes supported by dianionic alkoxy-amino-bisphenolate ligands: combining high activity, productivity, and selectivity. *Chem. Eur. J.* **12**, 169−179 (2006).
4. Seebach, D.; Hoffmann, T.; Kühnle, F. N. M.; Kinkel, J. N.; Schulte, M. Preparation, structure, and properties of all possible cyclic dimers (diolides) of 3-hydroxybutanoic acid. *Helvetica Chimica Acta* **78**, 1525−1540 (1995).
5. White, J. D.; Johnson, A. T. Synthesis of the Lichen Metabolite (+)-bourgeanic acid and conformational analysis of its dilactone. *J. Org. Chem.* **55**, 5938−5940 (1990).
6. Casiraghi, G.; Casnati, G.; Puglia, G.; Sartori, G.; Terenghi, G. Selective reactions between phenols and formaldehyde. A novel route to salicylaldehydes. *J. Chem. Soc., Perkin Trans. 1* 1862−1865 (1980).

7. DiCiccio, A. M.; Longo, J. M.; Rodríguez-Calero, G. G.; Coates, G. W. Development of highly active and regioselective catalysts for the copolymerization of epoxides with cyclic anhydrides: An unanticipated effect of electronic variation. *J. Am. Chem. Soc.* **138**, 7107−7113 (2016).

8. Kochnev, A. I.; Oleynik, I. I.; Oleynik, I. V.; Ivanchev, S. S.; Tolstikov, G. A. Synthesis of salicylaldehydes bearing bulky substituents in the positions 3 and 5. *Russ. Chem. Bull. Int. Ed.* **56**, 1125−1129 (2007).

9. Larrow, J. F.; Jacobsen, E. N.; Gao, Y.; Hong, Y.; Nie, X.; Zepp, C. M. A Practical method for the large-scale preparation of [*N,N'*-Bis(3,5-di-tert-butyl salicylidene)-1,2-cyclohexanediaminato2(-)] manganese (III) chloride, a highly enantioselective epoxidation catalyst. *J. Org. Chem.* **59**, 1939−1942 (1994).

10. Char, J.; Kulyk, O. G.; Brulé, E.; de Montigny, F.; Guérineau, V.; Roisnel, T.; Tschan, M. J. L.; Thomas, C. M. Microstructurally controlled polymers of *rac*-lactide by lithium complexes. *C. R. Chimie* **19**, 167−172 (2016).

11. Sanz, M.; Cuenca, T.; Galakhov, M.; Grassi, A.; Bott, R. K. J.; Hughes, D. L.; Lancaster, S. J.; Bochmann, M. Monocyclopentadienyl bis(phenoxo-imino)zirconium complexes as precatalyst for olefin polymerization. Stereospecific methylation of an imino group with formation of a zirconium–amido bond. *Organometallics* **23**, 5324−5331 (2004).

12. Liu, Q.; Meermann, C.; Görlitzer, H. W.; Runte, O.; Herdtweck, E.; Sirsch, P.; Törnroos, K. W.; Anwander, R. Cationic rare-earth metal SALEN complexes. *Dalton Trans.* 6170−6178 (2008).

13. Lin, M.-H.; RajanBabu, T. V. Ligand-assisted rate acceleration in transacylation by a yttrium-salen complex. Demonstration of a conceptually new strategy for metal-catalyzed kinetic resolution of alcohols. *Org. Lett.* **4**, 1607−1610 (2002).

14. Barham, P. J.; Keller, A.; Otun, E. L.; Holmes, P. A. Crystallization and morphology of a bacterial thermoplastic: poly-3-hydroxybutyrate. *J. Mater. Sci.* **19**, 2781−2794 (1984).

15. Ajellal, N.; Bouyahyi, M.; Amgoune, A.; Thomas, C. M.; Bondon, A.; Pillin, I.; Grohens, Y.; Carpentier, J.-F. Syndiotactic-enriched poly(3-hydroxybutyrate)s via stereoselective ring-opening polymerization of racemic *β*-butyrolactone with discrete yttrium catalysts. *Macromolecules* **42**, 987−993 (2009).

16. Hocking, P. J.; Marchessault, R. H. Microstructure of poly[(*R,S*)-*β*-hydroxybutyrate] by ^{13}C NMR. *Macromolecules* **28**, 6401−6409 (1995).

17. Kemnitzer, J. E.; McCarthy, S. P.; Gross, R. A. Preparation of predominantly syndiotactic poly(*β*-hydroxybutyrate) by the tributyltin methoxide catalyzed ring-opening polymerization of racemic *β*-butyrolactone. *Macromolecules* **26**, 1221−1229 (1993).

18. Bloembergen, S.; Holden, D. A. Bluhm, T. L.; Hamer, G. K.; Marchessault, R. H. Stereoregularity in synthetic *β*-hydroxybutyrate and *β*-hydroxyvalerate homopolyesters. *Macromolecules* **22**, 1656−1663 (1989).
